# Comparison with Dietary Groups of Various Macronutrient Ratios on Body Weight and Cardiovascular Risk Factors in Adults: A Systematic Review and Network Meta-Analysis

**DOI:** 10.3390/nu17162683

**Published:** 2025-08-19

**Authors:** Yiling Lou, Hengchang Wang, Linlin Wang, Shen Huang, Yulin Xie, Fujian Song, Zuxun Lu, Furong Wang, Qingqing Jiang, Shiyi Cao

**Affiliations:** 1 School of Public Health, Tongji Medical College, Huazhong University of Science and Technology, No. 13 Hangkong Road, Wuhan 430030, China; d202281742@hust.edu.cn (Y.L.); m202378133@hust.edu.cn (H.W.); m202375666@hust.edu.cn (L.W.); huangshen721@163.com (S.H.); m202275533@hust.edu.cn (Y.X.); zuxunlu@hust.edu.cn (Z.L.); 2 Norwich Research Park, Norwich Medical School, University of East Anglia, Norwich NR4 7TJ, UK; fujian.song@uea.ac.uk; 3 School of Nursing, Tongji Medical College, Huazhong University of Science and Technology, No. 13 Hangkong Road, Wuhan 430030, China; wangfurong.china@163.com (F.W.); 15084450940@163.com (Q.J.); 4 Department of Neurology, Tongji Hospital, Tongji Medical College, Huazhong University of Science and Technology, Wuhan 430030, China

**Keywords:** dietary carbohydrates, dietary fats, proteins, body weight, heart disease risk factors, network meta-analysis

## Abstract

**Background:** This network meta-analysis aimed to assess the relative efficacy of macronutrient dietary groups with varying carbohydrate, fat, and protein ratios on weight control and cardiovascular risk factors improvement in adults. **Methods:** We searched PubMed, the Cochrane Central Register of Controlled Trials (CENTRAL), Embase, Web of Science Core Collection, and ClinicalTrials.gov from inception to 30 November 2024, as well as reference lists of related systematic reviews. Eligible randomized controlled trials (RCTs) were included. Literature screening, data extraction, and risk of bias assessment were conducted independently by two reviewers. The changes in body weight, blood glucose, systolic blood pressure, diastolic blood pressure, high density lipoprotein (HDL) cholesterol, low density lipoprotein (LDL) cholesterol, triglycerides, and total cholesterol were the study outcomes. Utilizing a Bayesian framework, a series of random-effects network meta-analyses were conducted to estimate mean difference (MD) with 95% credible interval (CrI) and determine the relative effectiveness of the macronutrient dietary groups. The quality of evidence for each pair of dietary groups was assessed based on the online tool called confidence in network meta-analysis (CINeMA). **Results:** This study initially identified 14,988 studies and ultimately included 66 eligible RCTs involving 4301 participants in the analysis. The very low carbohydrate–low protein (VLCLP, MD −4.10 kg, 95% CrI −6.70 to −1.54), the moderate carbohydrate–high protein (MCHP, MD −1.51 kg, 95% CrI −2.90 to −0.20), the very low carbohydrate–high protein (VLCHP, MD −1.35 kg, 95% CrI −2.52 to −0.26) dietary groups might lead to weight loss compared with the moderate fat–low protein (MFLP) dietary group. Among the dietary groups relative to the MFLP dietary group, the moderate carbohydrate–low protein (MCLP, MD 0.09 mmol/L, 95% CrI 0.02 to 0.16) and VLCHP (MD 0.16 mmol/L, 95% CrI 0.08 to 0.24) dietary groups were less effective in lowering HDL cholesterol, and the VLCHP (MD 0.50 mmol/L, 95% CrI 0.26 to 0.75) dietary group was less effective in lowering LDL cholesterol. In terms of triglyceride reduction, the MCLP (MD −0.33 mmol/L, 95% CrI −0.44 to −0.22), VLCHP (MD −0.31 mmol/L, 95% CrI −0.42 to −0.18), VLCLP (MD −0.14 mmol/L, 95% CrI −0.25 to −0.02), and moderate fat–high protein (MFHP, MD −0.13 mmol/L, 95% CrI −0.21 to −0.06) dietary groups were more efficacious than the MFLP dietary group, while any pair of dietary group interventions showed minimal to no difference in the effects on blood glucose, blood pressure, and total cholesterol. **Conclusions:** High or moderate certainty evidence reveals that the VLCLP dietary group is the most appropriate for weight loss, while the MCLP dietary group is best for reducing triglycerides. For control of blood glucose, blood pressure, and cholesterol levels, there is little to no difference between macronutrient dietary groups. Additionally, future studies in normal-weight populations are needed to verify the applicability of our findings.

## 1. Introduction

Obesity and cardiovascular disease have become pervasive challenges in the realm of global public health with the changes in lifestyles and dietary habits [1,2]. In response, governments have recommended the use of diverse macronutrient dietary patterns (e.g., low-carbohydrate diet, low-fat diet, and high-protein diet) aimed at weight management and cardiovascular risk factors improvement. The World Health Organization (WHO) and some national dietary guidelines update the Dietary Reference Intakes (DRIs) of macronutrients every few years to advocate macronutrient dietary patterns [3,4,5,6]. Many macronutrient dietary patterns recommended by previous guidelines and most studies have primarily focused on DRIs for individual nutrients. However, focusing solely on the DRI of a single macronutrient may be unrealistic, because the human body typically ingests a variety of foods within a single meal in daily life to provide a rich blend of nutrients in different categories and proportions [7]. Moreover, the potential effects of interactions between different nutrient combinations on human health cannot be ignored. Research on macronutrient combinations is not only limited in quantity but also often constrained by poor participant adherence and unreliable evidence due to specific demographic characteristics of participants and wide variations in study durations [8,9]. Crucially, existing reviews mainly focus on recent popular diets [10,11], such as the Mediterranean diet, the ketogenic diet, and the Atkins diet, but fail to determine suitable dietary groups based on macronutrient ratios for weight management and cardiovascular risk factors improvement, thus potentially limiting their content comprehensiveness and practical value. Currently, it is important to determine the effects of diverse macronutrient dietary groups based on carbohydrate, fat, and protein ratios for weight management and cardiovascular risk factor improvement.

Despite previous studies having shown the positive effects of intake of carbohydrates, fats, and proteins for preventing obesity and chronic diseases [12,13], there is still a debate regarding the impact of dietary macronutrients on human health. Research suggests that obese individuals should choose a low-carbohydrate diet, as it can effectively reduce weight and control blood pressure in the short term to improve health [14], while a meta-analysis reported that a low-carbohydrate diet could be extremely damaging to long-term health [9]. In terms of weight control, the low-carbohydrate diet is often considered more effective than the low-fat diet [15,16], but one study has concluded that the two diets have no significant difference [17]. High-protein diet can improve blood glucose levels and insulin sensitivity [18,19], but it may also detrimentally affect heart, cardiovascular, and renal health [20]. It is imperative to acquire quantitative estimates comparing the impacts of various dietary groups consisting of different macronutrient ratios on diverse health needs. Network meta-analysis methods can compare interventions that are not directly comparable using both direct (head-to-head active interventions) and indirect evidence (intervention vs. inactive controls) to produce more precise quantitative estimates [21]. Therefore, this study performed a systematic network meta-analysis based on randomized controlled trials (RCTs) to explore the effects of macronutrient dietary groups with varying carbohydrate, fat, and protein ratios on controlling body weight and cardiovascular risk factors such as blood glucose levels, systolic and diastolic blood pressure, high-density lipoprotein (HDL) cholesterol, low-density lipoprotein (LDL) cholesterol, triglycerides, and total cholesterol. The analysis specifically tested the hypothesis that various macronutrient dietary groups have different effects on weight management and cardiovascular risk factor improvement, with the ultimate goal of identifying optimal dietary interventions for health indicators improvement and disease risk reduction.

## 2. Materials and Methods

The study protocol is registered with PROSPERO (CRD42024525613), and this review has been reported in line with Preferred Reporting Items for Systematic Reviews and Meta-Analyses (PRISMA) Guidelines [22].

### 2.1. Data Sources and Searches

We searched the following databases for the English-language literature: PubMed, the Cochrane Central Register of Controlled Trials (CENTRAL), Embase, and the Web of Science Core Collection from inception to 30 November 2024. Our search strategy involved keywords for diets, body weight, blood glucose, blood pressure, blood lipids outcomes, and RCTs. The search strategy details can be scrutinized in Appendices S1 and S2. Eligible trials at ClinicalTrials.gov and reference lists of related systematic reviews were also reviewed to identify additional studies.

### 2.2. Literature Screening and Study Selection

The English-language RCTs included in this study were required to meet the following rigorous criteria: (1) the study must compare the effects of at least two dietary groups with different macronutrient ratios of carbohydrates, fats, and proteins on cardiovascular outcomes in adults aged 18 years and older without major diseases, such as cancer; (2) the macronutrient composition of the dietary groups must be explicitly reported as stated in the original text, with the groups developed strictly in accordance with these macronutrient compositions; (3) the duration of the intervention must be a minimum of one week; (4) the study must provide pre-intervention and post-intervention values or post-intervention changes in the macronutrient dietary group intervention for one or more outcome measures, including body weight, blood glucose levels, systolic and diastolic blood pressure, HDL cholesterol, LDL cholesterol, triglycerides, and total cholesterol. Our analysis was focused on the last available follow-up of each study. The eligibility of each record was independently assessed by two reviewers (YL and HW), and any discrepancies were resolved through discussion or with the assistance of a third senior reviewer (SC).

We excluded (1) cross-over tests with wash-out period shorter than one week; (2) trials in patients with a history of cancer or major diseases, children, and adolescents; (3) studies not reporting primary outcomes.

**Figure 1:**
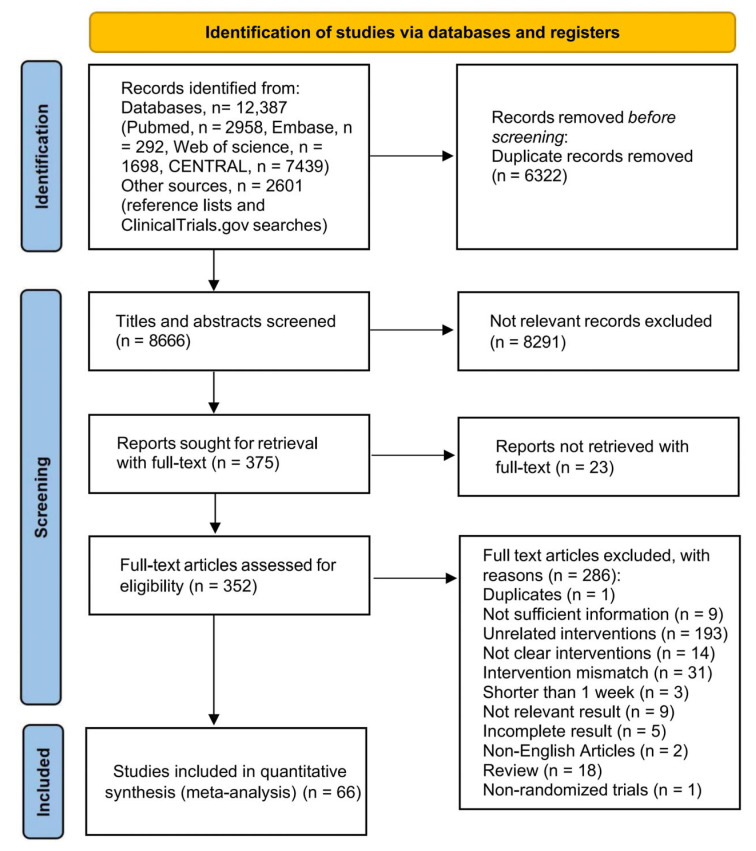
Flow diagram of literature selection. Study identification and selection presented according to the PRISMA 2020 flow diagram.

The PRISMA flow diagram of study selection is presented in Figure 1. Among four databases, a total of 12,387 unique studies were identified through electronic searches, with an additional 2601 records found through reference list screening and Clinical Trials.gov. We excluded duplicate articles, irrelevant content, articles for which full texts could not be retrieved, and those not meeting the inclusion criteria. Details on the studies excluded during the full-text assessment, along with reasons for exclusion, can be found in Supplementary Table S1. Finally, the RCTs that met the inclusion criteria were included in this study.

### 2.3. Data Extraction and Quality Assessment

Two reviewers (YL and HW) performed a search of studies from online databases and imported retrieval results to EndnoteX9 (Clarivate, Philadelphia, PA, USA) for removal of duplicates. Data were extracted using a standardized, pre-tested data extraction form and accompanying instructions, with information collected on study characteristics (year of publication, country, intervention duration), population (age, sex, sample size, body mass index), interventions (macronutrient ratios of carbohydrates, fats, and proteins in dietary groups), and outcomes (pre- and post-intervention measurements or changes after macronutrient dietary interventions in body weight, blood pressure, blood glucose, HDL, LDL, total cholesterol and triglycerides). The reviewers independently evaluated the risk of bias for each randomized controlled trial using the Cochrane risk of bias tool [23]. An overall risk of bias was classified for every study based on the individual risk of bias items [24,25]. The overall risk of bias was categorized as high, moderate, or low in this network meta-analysis [26].

### 2.4. Dietary Groups Classification

As determined by the different dietary interventions used in the identified studies, we divided the macronutrient dietary groups into several dietary categories based on different energy contribution ratios of carbohydrates, fats, and proteins in relation to total energy intake (Table 1): moderate fat–low protein (MFLP); moderate fat–high protein (MFHP); moderate carbohydrate–low protein (MCLP); moderate carbohydrate–high protein (MCHP); very low fat–low protein (VLFLP); very low fat–high protein (VLFHP); very low carbohydrate–low protein (VLCLP); very low carbohydrate–high protein (VLCHP) [5,27,28,29,30]. Considering that most usual diets in daily life are quite similar to the MFLP dietary group [31,32], we used it as the main control group for analysis. Our definition of dietary groups is consistent with the draft we registered with PROSPERO.

### 2.5. Data Synthesis and Analysis

We used the mean change and standard deviation (SD) of outcome indicators reported in the included literature for our analyses. When data was presented as pre- and post-intervention measures by the authors, the average and standard deviations for change were determined according to the guidelines of the Cochrane Handbook [23]. When standard deviations were not provided, they were estimated from standard errors, *p* values, confidence intervals, or graphs [33]. If either SD or standard error were not reported in the trials, the average SDs used in the corresponding analyses were obtained from other trials included in the study [33].

We performed statistical analyses for dietary groups based on eight nodes (MFLP, MFHP, MCLP, MCHP, VLFLP, VLFHP, VLCLP, and VLCHP). The pooled direct estimates of the available direct comparisons were obtained by using Bayesian random effects models [34]. A network plot was generated for each outcome to examine the network structure and identify potential direct and indirect comparisons. The Markov chain Monte Carlo simulations were all set up with a burn-in of 1000 iterations and a total of 10,000 iterations. A thinning interval of 10 was applied, which collected one sample every 10 iterations [35]. The network global inconsistency and local inconsistency between direct and indirect evidence were evaluated by the node-splitting method [36]. For all outcomes, the analysis generated a mean difference (MD) with a 95% credible interval (CrI) as the pooled estimate. Ranking list heat maps were utilized to visually compare the impact effect across the eight dietary groups. The SUCRA (surface under the curve cumulative ranking probabilities) and rankogram plots were chosen to show the ranking of dietary groups.

Two sensitivity analyses were conducted by restricting studies to trials involving individuals who were overweight and/or obese and those with a low risk of bias. The potential heterogeneity of intervention effects and the strength of our conclusions were assessed through subgroup network meta-analysis based on intervention duration (≤6 months/>6 months). Additionally, network meta-regression analyses were conducted to evaluate the potential effects of influencing factors including caloric restriction, physical activity, the country of study conduct, funding source, publication year, intervention period, and study sample size, on each outcome.

The funnel plots and Egger tests were used to assess publication bias [37]. The certainty of the evidence was assessed using the CINeMA (Confidence in Network Meta-Analysis) web application, which allows grading of confidence in the results as high, moderate, low, and very low [38]. Based on the GRADE (Grading of Recommendations Assessment, Development and Evaluation) framework and the contribution matrix of network meta-analysis, CINeMA integrates network meta-analysis as a whole, considering six aspects including within-study bias, across-study bias, indirectness, imprecision, heterogeneity, and incoherence, to grade the quality of evidence [39]. We assessed intransitivity for the CINeMA framework by comparing potential effect modifiers, such as baseline age and BMI, between studies that provided direct and indirect evidence for each comparison. After importing the extracted data into the CINeMA, the generated confidence intervals and prediction intervals were used to evaluate the heterogeneity of the included RCTs [40].

Network meta-analysis was performed using the “network” package in Stata (release 14, Stata-Corp LLC, College Station, TX, USA) [41] and packages multinma and BUGSnet in R (version 4.3.2, R Foundation for Statistical Computing, Vienna, Austria) [42].

## 3. Results

### 3.1. Characteristics of the Included RCTs

This network meta-analysis ultimately included 66 eligible RCTs, and the general characteristics of these studies are listed in Supplementary Table S2. The 66 RCTs covered 4301 participants, and 3745 participants completed the trial. Among the RCTs identified, 56 trials (84.8%) addressed weight outcomes, 46 trials (69.7%) addressed blood glucose outcomes, 29 trials (43.9%) addressed blood pressure outcomes, and 47 trials (71.2%) addressed blood lipid outcomes. The included studies were published from 1977 to 2024, with 59 trials (89.4%) having a parallel design and 7 trials (10.6%) having a cross-over design. The follow-up duration ranged from 1 to 96 weeks; the intervention periods consisted of 45 trials (68.2%) with a duration of ≤6 months; and 21 trials (31.8%) > 6 months. Of the 66 eligible trials, 2 trials (3%) included normal-weight adults (BMI 18.5–24.9 kg/m^2^), 62 trials (93.9%) included adults with overweight and obesity (BMI ≥ 25 kg/m^2^) [1], and 2 trials (3%) not specifying BMI of subjects. In terms of methodological quality assessment, 43 trials (65.2%) were judged to have a low overall risk of bias, and 21 trials (31.8%) were at moderate risk, leaving only 2 trials (3%) that were at high risk (Supplementary Table S3, Figures S1 and S2).

### 3.2. Network Meta-Analysis Results

**Figure 2:**
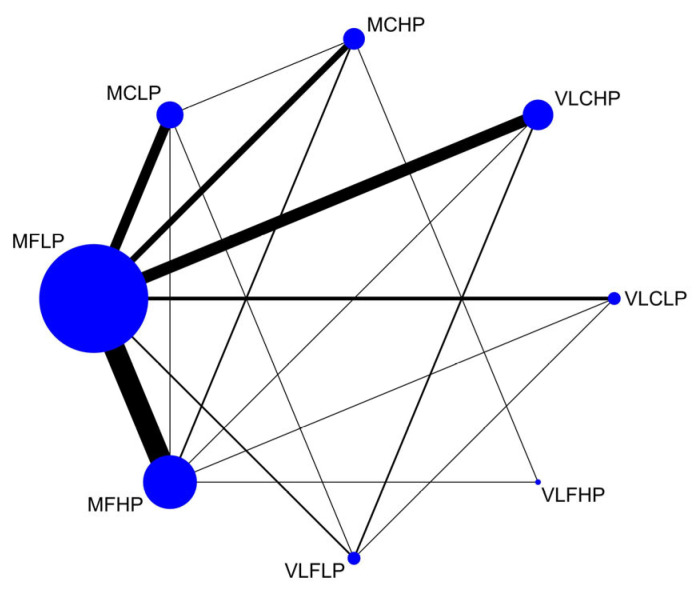
Network plot of all included trials for dietary group. The size of the nodes is proportional to the total number of participants assigned to each trial, lines between nodes show a direct comparison, and the thickness of the lines is proportional to the number of trials assessed for each direct comparison. Abbreviations: MFLP, moderate fat–low protein; MFHP, moderate fat–high protein; MCLP, moderate carbohydrate–low protein; MCHP, moderate carbohydrate–high protein; VLFLP, very low fat–low protein; VLFHP, very low fat–high protein; VLCLP, very low carbohydrate–low protein; VLCHP, very low carbohydrate–high protein.

Figure 2 provides the network plot for direct comparison of dietary groups with different levels of carbohydrate, fat, and protein composition, and the most common comparisons were between MFLP and MFHP dietary groups. Supplementary Figures S3–S10 present all network plots for each outcome. Supplementary Figure S11 presents the ranking of different macronutrient dietary groups across all outcome indicators. Supplementary Table S4 and Figures S12–S27 present findings of the global and local inconsistency tests for each outcome, with significant direct and indirect evidence of consistency for all outcomes, except the HDL cholesterol outcomes. Supplementary Figures S28–S35 show the results of funnel plots and Egger tests for the different outcomes, which show that only the inclusion of the diastolic blood pressure and HDL cholesterol outcomes had a high publication bias, and the inclusion of the remaining outcomes had a low publication bias. Supplementary Table S5 demonstrates that all networks adhere to the principle of transitivity, thereby enhancing the validity of indirect comparisons. Supplementary Tables S6–S13 present grading evidence for all outcomes of the network meta-analysis using CINeMA, with the number of included RCTs, nature of evidence, confidence level, and downgrading reason. Much of the evidence was judged as low certainty, and most of the studies were downgraded due to imprecision (wide credible intervals) in intervention effect estimates.

### 3.3. Effects of Macronutrient Dietary Groups on Outcome Indicators

#### 3.3.1. Weight Change

**Figure 3:**
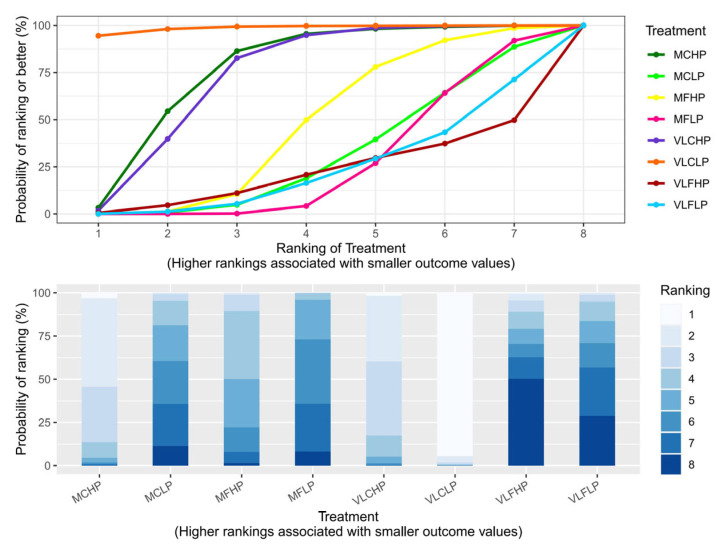
SUCRA and rankogram charts of weight change. The upper graph represents a SUCRA chart, indicating that a higher curve in the dietary group corresponds to a more effective weight loss outcome; The lower graph displays a rankogram chart, which quantifies the probability of each dietary group achieving each possible rank (1 to 8). Lighter colours indicate higher rankings. For example, the VLCLP diet group had the largest area of lightest colour, indicating the highest probability of ranking first for weight loss efficacy. Abbreviations: MFLP, moderate fat–low protein; MFHP, moderate fat–high protein; MCLP, moderate carbohydrate–low protein; MCHP, moderate carbohydrate–high protein; VLFLP, very low fat–low protein; VLFHP, very low fat–high protein; VLCLP, very low carbohydrate–low protein; VLCHP, very low carbohydrate–high protein; SUCRA, surface under the curve cumulative ranking probabilities.

**Figure 4:**
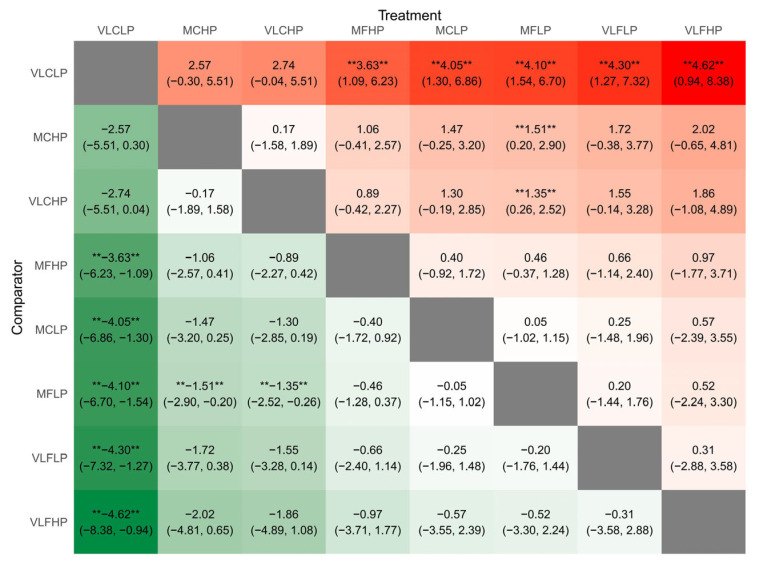
Ranking list heat map of weight change. “Treatment” represents the intervention and “Comparator” represents the control. The values indicate the mean difference (MD) and 95% confidence interval between the macronutrient dietary groups. The symbol (**) indicates that there is a statistically significant difference between “Treatment” and “Comparator” at the 95% confidence interval level. Abbreviations: MFLP, moderate fat–low protein; MFHP, moderate fat–high protein; MCLP, moderate carbohydrate–low protein; MCHP, moderate carbohydrate–high protein; VLFLP, very low fat–low protein; VLFHP, very low fat–high protein; VLCLP, very low carbohydrate–low protein; VLCHP, very low carbohydrate–high protein.

The SUCRA and rankogram charts showed the VLCLP dietary group was the highest ranked for losing weight (Figure 3, Supplementary Figure S36). Figure 4 presents the relative effectiveness comparison between all interventions, displaying the MD in weight loss effects with 95% CrI for each intervention group versus the control group. When compared with the MFLP dietary group, the VLCLP (MD −4.10 kg, 95% CrI −6.70 to −1.54), MCHP (MD −1.51 kg, 95% CrI −2.90 to −0.20), and VLCHP (MD −1.35 kg, 95% CrI −2.52 to −0.26) dietary groups might lead to weight loss based on high certainty evidence, while the MFHP, MCLP, MFLP, VLFLP, and VLFHP dietary groups had little or no beneficial effect on weight loss based on low certainty evidence.

#### 3.3.2. Blood Glucose

In terms of reducing blood glucose, the MCLP and VLCLP dietary groups were both the highest ranked for reducing blood glucose in the SUCRA and rankogram charts (Supplementary Figures S37 and S38). This indicates the MCLP and VLCLP dietary groups were the highest efficacy in reducing blood glucose. Nevertheless, when compared with the MFLP dietary group, there was no statistically significant difference in the blood glucose reduction effects among the remainder dietary groups based on low or very low certainty evidence (Supplementary Figure S39).

#### 3.3.3. Blood Pressure

Supplementary Figures S40–S44 show that the VLCHP dietary group ranked highest in lowering systolic blood pressure, with the MFHP dietary group ranking highest for diastolic blood pressure. However, there was no statistically significant difference in the blood pressure reduction effects among the seven dietary groups based on low or very low certainty evidence (Supplementary Figures S42 and S45).

#### 3.3.4. Blood Lipids

The SUCRA and rankogram charts showed the MFHP and VLFLP dietary groups were the highest ranked for lowering HDL, as well as in lowering LDL and total cholesterol. (Supplementary Figures S46–S51). When compared with the MFLP dietary group, the MCLP (MD 0.09 mmol/L, 95% CrI 0.02 to 0.16) and VLCHP (MD 0.16 mmol/L, 95% CrI 0.08 to 0.24) dietary groups were less effective in lowering HDL cholesterol, while the MFHP, VLFLP, VLFHP, MCHP, and VLCLP dietary groups had little or no beneficial effect in lowering HDL cholesterol based on low or very low evidence (Supplementary Figure S52). Among the dietary groups with low or very low evidence relative to the MFLP dietary group, the VLCHP (MD 0.50 mmol/L, 95% CrI 0.26 to 0.75) dietary group was less effective in lowering LDL cholesterol, while the MFHP, VLFLP, MCLP, MCHP, VLCLP, and VLFHP dietary groups had little or no beneficial effect in lowering LDL cholesterol based on low or very low evidence (Supplementary Figure S53). Based on low or very low certainty evidence, there was no statistically significant difference in lowering total cholesterol effects among the remainder of dietary groups compared with the MFLP dietary group (Supplementary Figure S54).

The MCLP dietary group was the highest ranked for lowering triglycerides (Supplementary Figures S55 and S56). As shown in Supplementary Figure S57, when compared with the MFLP dietary group, the MCLP, (MD −0.33 mmol/L, 95% CrI −0.44 to −0.22), VLCHP (MD −0.31 mmol/L, 95% CrI −0.42 to −0.18), VLCLP (MD −0.14 mmol/L, 95% CrI −0.25 to −0.02), and MFHP dietary groups (MD −0.13 mmol/L, 95% CrI −0.21 to −0.06) might lead to triglyceride reduction based on moderated or high certainty evidence, while the VLFLP, MCHP, and VLFHP dietary groups had little or no beneficial effect on triglyceride reduction based on low-certainty evidence.

### 3.4. Network Meta-Regression and Sensitivity Analysis Results

Our network meta-regression did not find any substantial difference when determining the potential effects of influence factors (caloric restriction, physical activity, country in which the study was conducted, funding source, publication year, intervention period, and study sample size). The results for each outcome illustrated that none of the regression factors had statistically significant effects (Supplementary Table S14). The results of subgroup analyses showed that the duration of the intervention, whether more than 6 months or less than 6 months, resulted in similar outcomes to the main results (Supplementary Figures S58–S73).

The results of the sensitivity analyses for weight, blood glucose, blood pressure and blood lipids by restricting the analyses to studies with a low risk of bias (Supplementary Figures S74–S81) and studies conducted on participants who were overweight and/or obese (Supplementary Figures S82–S85) were all similar to our primary outcome analysis. In addition, sensitivity analyses for blood lipid outcomes were not conducted in the trial limited to overweight and/or obese participants due to insufficient data. The results show that these influencing factors have no significant effect on the outcome and that our main outcome analysis is robust.

## 4. Discussion

Our network meta-analysis quantifies the relative effectiveness of eight macronutrient dietary groups for controlling body weight and cardiovascular risk factors. Moderate- or high-certainty evidence showed that three macronutrient dietary groups (VLCLP, MCHP, and VLCHP) were significantly associated with larger reductions in body weight (Range −1.35 to −4.10 kg) and four macronutrient dietary groups (MCLP, VLCHP, VLCLP, and MFHP) were significantly associated with larger reductions in triglyceride (range: −0.13 to −0.33 mmol/L) than the MFLP dietary group. However, based on low- or very-low certainty evidence, there is no significant difference among the macronutrient dietary groups in terms of lowering blood glucose and blood pressure. In addition, significant differences in cholesterol reduction were observed only in a few macronutrient dietary groups.

At present, the recommended macronutrient intake levels in the dietary guidelines of various countries are most closely aligned with those of the MFLP dietary group, which is also considered as one of the preferred options for preventing chronic diseases such as obesity and cardiovascular diseases [43]. Interestingly, this review found that the MFLP dietary group was not the most effective in controlling weight or blood pressure. The reason for selecting a dietary intervention similar in nutrient composition to the MFLP group as the primary recommendation is that other dietary groups that are designed for specific populations or individual nutritional needs may not be universally effective for all populations, whereas the MFLP group likely provides a balanced and adequate nutrient profile for a general population [44]. Moreover, the same dietary group exhibited varying effects on different health outcomes. For instance, despite the VLCLP dietary group being beneficial for weight loss, it showed limited impact on lowering blood lipids. This suggested that individuals with high blood lipids may not benefit from choosing the VLCLP dietary group as the optimal weight loss diet. Our results clearly compared the effects of each macronutrient dietary group for weight, blood glucose, blood pressure, and blood lipids. These findings and data presentations are extremely important for populations seeking to lose weight or reduce cardiovascular risk factors using a healthy macronutrient dietary group.

Our review has several advantages. Firstly, our research aims to assist individuals in effectively managing their weight and preventing cardiovascular diseases, ultimately reducing the societal burden associated with these conditions. Secondly, to our knowledge, our study is the first network meta-analysis to compare the relative efficacy of dietary groups with different energy contribution ratios of carbohydrates, fats, and proteins on weight and cardiovascular risk factors. By combining direct and indirect comparisons, our analysis could provide a comprehensive estimate on weight and cardiovascular risk factors in relation to macronutrient dietary groups, thereby identifying the optimal ratio of the three macronutrients that could manage weight and improve cardiovascular risk factors. Thirdly, we strictly adhered to standard guidelines to implement our research, including systematic literature search, reasonable analysis process, and scientific evidence assessment. A publicly available study protocol was also published on PROSPERO in advance, which ensured the reliability of our results. Finally, our review used the sophisticated CINeMA application to elucidate and evaluate the reliability of the evidence [39] and provided tables of results that emphasize the certainty of the evidence.

Our analysis also has some limitations. Firstly, the CINeMA results indicated the presence of heterogeneity among a minority of included studies, which may be attributed to inconsistent definitions of macronutrient dietary groups among different studies. Currently, there is no uniform definition of macronutrient dietary groups with varying carbohydrate, fat, and protein ratios across countries [27,45]. Although we tried our best to scientifically define our dietary groups based on authoritative dietary guidelines and the relative literature, heterogeneity and within-study bias between the partially included studies remain, and the pervasiveness of the findings may still be limited. Secondly, partial comparisons provide only low-certainty evidence due to imprecision and inconsistency between direct and indirect evidence, as well as potential risks of reporting bias and within-study bias. The few numbers of studies with direct comparisons of some macronutrient dietary groups may be an important reason for imprecision and inconsistency between direct and indirect evidence. In order to mitigate the impact of insufficient direct comparisons on the study, we also searched reference lists and ClinicalTrials.gov in addition to the four major databases. However, more RCTs are still needed to explore the association between macronutrient dietary groups and weight or cardiovascular risk factors. Thirdly, participants included in this review were not informed about either the planning or the conduct of the procedures of the study, which reduced their intervention in the trial. However, this may result in subjects not fully comprehending the purpose of the experiment and thus reducing their compliance with the experimental protocol and increasing the likelihood of selection bias, which potentially affects the relevance, feasibility, and acceptability of the research findings [46]. Fourthly, although this study ranks seven dietary groups by their blood pressure and glucose-lowering efficacy, we acknowledge the lack of significant effects on these outcomes across the dietary groups. Future research may need to explore more dietary groups with varying macronutrient ratios to identify the optimal diet for lowering blood pressure and blood glucose. Lastly, the majority of study data included in this analysis were derived from obese individuals. Of the 66 eligible RCTs, with only 2 out of 66 eligible RCTs (3%) including adults with normal weight. Although we performed sensitivity analyses, subgroup analyses, and network meta-regression that showed our results as robust, this still may lead to limited applicability of the study results to the public.

The WHO and some national dietary guidelines are usually updated every few years, and these guidelines are generally considered the most authoritative dietary recommendations. The macronutrient dietary groups in our study are also divided based on these guidelines and the relative literature. These dietary recommendations are typically developed to meet the general health needs of the public, with an emphasis on providing balanced nutrition, while our research provides pertinent dietary recommendations for different health needs groups, which is one of the innovative aspects of our research. In recent years, researchers have worked to study the effects of various recommended dietary regimens on weight and chronic disease. Lukas et al. did a network meta-analysis of nine dietary approaches on glycaemic control in patients with type 2 diabetes mellitus [47]; Ge et al. [10] did a network meta-analysis of fourteen popular dietary programmes on body weight and cardiovascular risk factors; and Giorgio et al. [11] did a network meta-analysis of seven popular dietary programmes on cardiovascular disease morbidity and mortality. These reviews focus on recent popular dietary programmes, such as Mediterranean, Ornish, and Paleolithic diets. While our review is centred on dietary groups of varying carbohydrate, fat, and protein ratios, these findings may help adjust the intake proportions of the macronutrients based on an individual’s health condition to achieve optimal nutrient balance and more accurately meet the body’s nutritional needs.

In 2020, Chawla’s team did a meta-analysis comparing the effects of a low-fat diet versus a low-carbohydrate diet on body weight and lipid grades [15], and Joshua’s team did a meta-analysis comparing the effects of a low-carbohydrate diet versus a very low-carbohydrate diet on body weight and diabetes-related markers [48]. They found that there was a significant discrepancy in weight loss between low-fat and low-carbohydrate diets, as well as between low-carbohydrate and very-low-carbohydrate diets, which is consistent with our findings. The consistency could be attributed to the similarity between our study and the intervention group definitions in these studies, further indicating the scientific validity of the definition of the macronutrient dietary groups in our study. What is more, our study has revealed intriguing findings: both high-protein diets and low-carbohydrate diets exhibit positive impacts on weight loss [49,50]. Nevertheless, the effectiveness of the VLCHP dietary group on weight loss is inferior to that of the VLCLP dietary group. This discrepancy may be attributed to macronutrient interactions, warranting further research for subsequent investigation.

## 5. Conclusions

In conclusion, different macronutrient dietary groups are needed to address the diversified health demands of populations. High- or moderate-certainty evidence shows that people aiming for weight loss may first consider the VLCLP dietary group. Moderate-certainty evidence shows that those aiming for triglyceride reduction might prioritize the MCLP dietary group. For control of blood glucose, blood pressure, and cholesterol levels, there is little to no difference between macronutrient dietary group interventions based on different macronutrient ratios. Notably, generalizability is limited as most included studies focused on overweight or obese participants; further research in normal weight populations is needed to confirm broader applicability.

## Figures and Tables

**Table 1:** Dietary groups based on dietary intake of various fats, proteins, and carbohydrates.

	Dietary Group	Carbohydrates, % Kcal	Protein, % Kcal	Fat, % Kcal
MFLP	Moderate fat–low protein diet	30 < Eng	30 > Eng	10 < Eng ≤ 30
MFHP	Moderate fat–high protein diet	30 < Eng	30 ≤ Eng	10 < Eng ≤ 30
MCLP	Moderate carbohydrate–low protein diet	10 < Eng ≤ 30	30 > Eng	30 < Eng
MCHP	Moderate carbohydrate–high protein diet	10 < Eng ≤ 30	30 ≤ Eng	30 < Eng
VLFLP	Very low fat–low protein diet	30 < Eng	30 > Eng	10 ≥ Eng
VLFHP	Very low fat–high protein diet	30 < Eng	30 ≤ Eng	10 ≥ Eng
VLCLP	Very low carbohydrate–low protein diet	10 ≥ Eng	30 > Eng	30 < Eng
VLCHP	Very low carbohydrate–high protein diet	10 ≥ Eng	30 ≤ Eng	30 < Eng

Eng: the energy provided by carbohydrates, fats, and proteins in different dietary groups.

## Data Availability

The manuscript’s guarantor (Shiyi Cao) affirms that the manuscript is an honest, accurate, and transparent account of the study being reported; that no important aspects of the study have been omitted; and that any discrepancies from the study as originally planned (and, if relevant, registered) have been explained. The guarantor (Shiyi Cao) is willing to examine all requests for the full dataset after a period of two years from the date of this publication. The corresponding author should be contacted at caoshiyi@hust.edu.cn.

## Supplementary Materials

The following supporting information can be downloaded at: https://www.mdpi.com/article/10.3390/nu17162683/s1. The Supplementary Materials include Appendices S1 and S2, Supplementary Tables S1–S14, and Supplementary Figures S1–S85. References [51,52,53,54,55,56,57,58,59,60,61,62,63,64,65,66,67,68,69,70,71,72,73,74,75,76,77,78,79,80,81,82,83,84,85,86,87,88,89,90,91,92,93,94,95,96,97,98,99,100,101,102,103,104,105,106,107,108,109,110,111,112,113] are cited in Supplementary Materials.
