# Supplementary material for: Comparison with Dietary Groups of Various Macronutrient Ratios on Body Weight and Cardiovascular Risk Factors in Adults: A Systematic Review and Network Meta-Analysis
Source: Nutrients. 2025 Aug 19;17(16):2683. doi: 10.3390/nu17162683 (PMC12389190; doi:10.3390/nu17162683)
Supplement: Supplementary file 1 [file nutrients-17-02683-s001.zip › Supplementary Materials .pdf]

Comparison with dietary groups of various macronutrient ratios on body weight and cardiovascular risk factors in adults: a systematic review and network meta-analysis

content

Appendix S1: Search strategy. 1
Appendix S2:Search update (November, 2024) 8
Table S1: List of trials excluded from the analyses. 8
Table S2: Characteristics of included studies. 9
Table S3: Quality of trials included in the network meta-analysis of dietary groups. 13
Figure S1-2: Risk of bias summary for the 66 included trials. 16
Figure S3-S10: Network plots for each outcome. 17
Figure S11 Rank- heat plot of SUCRA for eight macronutrient dietary groups with all outcome indicators 21
Table S4: Inconsistency test of network meta-analysis 22
Figure S12-S19: Global inconsistency test chart for each outcome. 23
Figure S20-S27: Local inconsistency test chart for each outcome. 31
Figure S28-S35: The funnel plots and egger's test values for each outcome. 39
Table S5: Transitivity (Indirectness) Assessment 43
Table S6: CINeMA for the outcome "weight change" in dietary groups NMA. 44
Table S7: CINeMA for the outcome "blood glucose" in dietary groups NMA. 45
Table S8: CINeMA for the outcome "systolic blood pressure" in dietary groups NMA. 46
Table S9: CINeMA for the outcome "diastolic blood pressure" in dietary groups NMA. 47
Table S10: CINeMA for the outcome "HDL cholesterol" in dietary groups NMA. 48
Table S11: CINeMA for the outcome "LDL cholesterol" in dietary groups NMA. 49
Table S12: CINeMA for the outcome "triglyceride" in dietary groups NMA. 50
Table S13: CINeMA for the outcome "total cholesterol" in dietary groups NMA. 51
Figure S36: Intervention ranking chart for weight change. 52
Figure S37-39: Network meta-analysis of blood glucose. 53
Figure S40-42: Network meta-analysis of systolic blood pressure 55
Figure S43-45: Network meta-analysis of diastolic blood pressure. 57
Figure S46-57: Network meta-analysis of Blood lipids. 59
Table S14: Values of network meta regression coefficients for different covariates in different outcomes. 65
Figure S58-65: Ranking plots of each outcome in subgroups. 66
Figure S66-73: Ranking list heat maps of each outcome in subgroups. 70
Figure S74-81: Sensitivity analyses limiting the study to low risk of bias. 74
Figure S82-85: Sensitivity analyses restricting the study to overweight and/or obese participants. 78

Appendix S1: Search strategy.

1.Weight change

PUBMED

1. Body Weight[Mesh] OR (Body Weights OR Weight, Body OR Weights, Body)
2. Diet, Carbohydrate-Restricted[Mesh] OR (Diet, Carbohydrate Restricted OR Diet, Low Carbohydrate OR Carbohydrate Diet, Low OR Carbohydrate Diets, Low OR Diets, Low Carbohydrate OR Low Carbohydrate Diets OR Carbohydrate-Restricted Diet OR Carbohydrate Restricted Diet OR Carbohydrate-Restricted Diets OR Diets, Carbohydrate-Restricted OR Low-Carbohydrate Diet OR Diet, Low-Carbohydrate OR Diets, Low-Carbohydrate OR Low Carbohydrate Diet OR Low-Carbohydrate Diets)
3. Diet, Fat-Restricted[Mesh]) OR (Diet, Fat Restricted OR Fat-Restricted Diet OR Diets, Fat-Restricted OR Fat Restricted Diet OR Fat-Restricted Diets OR Diet, Low-Fat OR Diet, Low Fat OR Diets, Low-Fat OR Low-Fat Diet OR Low-Fat Diets OR Diet, Fat-Free OR Diet, Fat Free OR Diets, Fat-Free OR Fat-Free Diet OR Fat-Free Diets)
4. 1 AND 2 AND 3
5. Diet, High-Protein[Mesh] OR (Diet, High Protein OR Diets, High-Protein OR High-Protein Diets OR High-Protein Diet OR High Protein Diet)
6. 1 AND 2 AND 5
7. 1 AND 3 AND 5
8. 4 OR 6 OR 7
9. (randomized controlled trial [Publication Type]) OR (controlled clinical trial [Publication Type]) OR ("clinical trials as topic" [Mesh]) OR (randomized [Title/Abstract]) OR (randomly [Title/Abstract]) OR (random [Title/Abstract]) OR (trial [Title/Abstract])
10. ("1960/1/1"[Date - Publication] : "2023/9"[Date - Publication])
11. 8 AND 9 AND 10
12. ("animals" [Mesh]) NOT ("humans" [Mesh])
13. 11 NOT 12

EMBASE

1. (Body Weight or Body Weights or Weight, Body or Weights, Body).af.
2. (Diet, Carbohydrate-Restricted* or low carbohydrate or low carbohydrate diet or low carbohydrate or carbohydrate restricted).af.
3. (Diet, Fat-Restricted* or diet, low fat or low fat diet or low fat or fat restricted).af.
4. 1 and 2 and 3
5. (diet, high-protein or diet, high protein or diets, high-protein or high-protein diets or high-protein diet or high protein diet).af.
6. 1 and 2 and 5
7. A and 3 and 5
8. 4 or 6 or 7
9. English.lg
10. 8 and 9
11. Add filters for: Publication Year: 1947-2023, subject: clinical trial, publication type: Article

Web of science search: selecting Web of Science Core Collection databases

1. Body Weight (Topic) or Body Weights (Topic) or Weight, Body (Topic) or Weights, Body (Topic)
2. Diet, Carbohydrate-Restricted (Topic) or Diet, Carbohydrate Restricted (Topic) or Diet, Low Carbohydrate (Topic) or Carbohydrate Diet, Low (Topic) or Carbohydrate Diets, Low (Topic) or Diets, Low Carbohydrate (Topic) or Low Carbohydrate Diets (Topic) or Carbohydrate-Restricted Diet (Topic) or Carbohydrate Restricted Diet (Topic) or Carbohydrate-Restricted Diets (Topic) or Diets, Carbohydrate-Restricted (Topic) or Low-Carbohydrate Diet (Topic) or Diet, Low-Carbohydrate (Topic) or Diets, Low-Carbohydrate (Topic) or Low Carbohydrate Diet (Topic) or Low-Carbohydrate Diets (Topic)
3. Diet, Fat-Restricted (Topic) or Diet, Fat Restricted (Topic) or Fat-Restricted Diet (Topic) or Diets, Fat-Restricted (Topic) or Fat Restricted Diet (Topic) or Fat-Restricted Diets (Topic) or Diet, Low-Fat (Topic) or Diet, Low Fat (Topic) or Diets, Low-Fat (Topic) or Low-Fat Diet (Topic) or Low-Fat Diets (Topic) or Diet, Fat-Free (Topic) or Diet, Fat Free (Topic) or Diets, Fat-Free (Topic) or Fat-Free Diet (Topic) or Fat-Free Diets (Topic)
4. 1 and 2 and 3
5. diet, high-protein (Topic) or diet, high protein (Topic) or diets, high-protein (Topic) or high-protein diets (Topic) or high-protein diet (Topic) or high protein diet (Topic)
6. 1 and 2 and 5
7. 1 and 3 and 5
8. 4 or 6 or 7
9. Add filter: Language: English, Document type: Article
10. randomized controlled trial (All Fields) or pragmatic clinical trial (All Fields)
11. 1970-01-01/2023-09-30 (Publication Date)
12. 8 and 9 and 10 and 11

Cochrane Central Register of Controlled Trials (CENTRAL)

1. MeSH descriptor: [Body Weight] explode all trees
2. Body Weight or Body Weights or Weight, Body or Weights, Body
3. 1 or 2
4. MeSH descriptor: [Diet, Carbohydrate-Restricted] explode all trees
5. Diet, Carbohydrate-Restricted or Diet, Carbohydrate Restricted or Diet, Low Carbohydrate or Carbohydrate Diet, Low or Carbohydrate Diets, Low or Diets, Low Carbohydrate or Low Carbohydrate Diets or Carbohydrate-Restricted Diet or Carbohydrate Restricted Diet or Carbohydrate-Restricted Diets or Diets, Carbohydrate-Restricted or Low-Carbohydrate Diet or Diet, Low-Carbohydrate or Diets, Low-Carbohydrate or Low Carbohydrate Diet or Low-Carbohydrate Diets
6. 4 or 5
7. MeSH descriptor: [Diet, Fat-Restricted] explode all trees
8. Diet, Fat-Restricted or Diet, Fat Restricted or Fat-Restricted Diet or Diets, Fat-Restricted or Fat Restricted Diet or Fat-Restricted Diets or Diet, Low-Fat or Diet, Low Fat or Diets, Low-Fat or Low-Fat Diet or Low-Fat Diets or Diet, Fat-Free or Diet, Fat Free or Diets, Fat-Free or Fat-Free Diet or Fat-Free Diets

9. 7 or 8
10. 3 and 6 and 9
11. MeSH descriptor: [Diet, High-Protein] explode all trees
12. diet,high-protein or diet,high protein or diets,high-protein or high-protein diets or high-protein diet or high protein diet
13. 11 or 12
14. 3 and 6 and 13
15. 3 and 9 and 13
16. 10 or 14 or 15

2.Blood glucose and blood pressure

PUBMED

1. Blood Pressure[Mesh] OR (Pressure, Blood OR Diastolic Pressure OR Pressure, Diastolic OR Pulse Pressure OR Pressure, Pulse OR Systolic Pressure OR Pressure, Systolic OR Pressures, Systolic)
2. Glucose"[Mesh] OR (Blood Sugar OR Sugar, Blood OR Glucose, Blood)
3. 1 OR 2
4. Diet, Carbohydrate-Restricted[Mesh] OR (Diet, Carbohydrate Restricted OR Diet, Low Carbohydrate OR Carbohydrate Diet, Low OR Carbohydrate Diets, Low OR Diets, Low Carbohydrate OR Low Carbohydrate Diets OR Carbohydrate-Restricted Diet OR Carbohydrate Restricted Diet OR Carbohydrate-Restricted Diets OR Diets, Carbohydrate-Restricted OR Low-Carbohydrate Diet OR Diet, Low-Carbohydrate OR Diets, Low-Carbohydrate OR Low Carbohydrate Diet OR Low-Carbohydrate Diets)
5. Diet, Fat-Restricted[Mesh]) OR (Diet, Fat Restricted OR Fat-Restricted Diet OR Diets, Fat-Restricted OR Fat Restricted Diet OR Fat-Restricted Diets OR Diet, Low-Fat OR Diet, Low Fat OR Diets, Low-Fat OR Low-Fat Diet OR Low-Fat Diets OR Diet, Fat-Free OR Diet, Fat Free OR Diets, Fat-Free OR Fat-Free Diet OR Fat-Free Diets)
6. 3 AND 4 AND 5
7. Diet, High-Protein[Mesh] OR (Diet, High Protein OR Diets, High-Protein OR High-Protein Diets OR High-Protein Diet OR High Protein Diet)
8. 3 AND 4 AND 7
9. 3 AND 5 AND 7
10. 6 OR 8 OR 9
11. (randomized controlled trial [Publication Type]) OR (controlled clinical trial [Publication Type]) OR ("clinical trials as topic" [Mesh]) OR (randomized [Title/Abstract]) OR (randomly [Title/Abstract]) OR (random [Title/Abstract]) OR (trial [Title/Abstract])
12. ("1960/1/1"[Date - Publication] : "2023/9"[Date - Publication])
13. 10 AND 11 AND 12
14. ("animals" [Mesh]) NOT ("humans" [Mesh])
15. 13 NOT 14

EMBASE

1. (Blood Pressure or Pressure, Blood or Diastolic Pressure or Pressure, Diastolic or Pulse Pressure or Pressure, Pulse or Systolic Pressure or Pressure, Systolic or Pressures, Systolic).af.
2. (Blood Glucose or Blood Sugar or Sugar, Blood or Glucose, Blood).af.
3. 1 or 3
4. (Diet, Carbohydrate-Restricted or low carbohydrate or low carbohydrate diet or low carbohydrate or carbohydrate restructed).af.
5. (Diet, Fat-Restricted or diet, low fat or low fat diet or low fat or fat restricted).af.
6. 3 and 4 and 5
7. (diet,high-protein or diet,high protein or diets,high-protein or high-protein diets or high-protein diet or high protein diet).af.
8. 3 and 4 and 7
9. 3 and 5 and 7
10. 6 or 8 or 9
11. English.lg
12. 8 and 9
13. Add filters for: Publication Year: 1947-2023, subject: clinical trial, publication type: Article

Web of science search: selecting Web of Science Core Collection databases

1. Blood Pressure (Topic) or Pressure, Blood (Topic) or Diastolic Pressure (Topic) or Pressure, Diastolic (Topic) or Pulse Pressure (Topic) or Pressure, Pulse (Topic) or Systolic Pressure (Topic) or Pressure, Systolic (Topic) or Pressures, Systolic (Topic)
2. Blood Glucose (Topic) or Blood Sugar (Topic) or Sugar, Blood (Topic) or Glucose, Blood (Topic)
3. 1 or 2
4. Diet, Carbohydrate-Restricted (Topic) or Diet, Carbohydrate Restricted (Topic) or Diet, Low Carbohydrate (Topic) or Carbohydrate Diet, Low (Topic) or Carbohydrate Diets, Low (Topic) or Diets, Low Carbohydrate (Topic) or Low Carbohydrate Diets (Topic) or Carbohydrate-Restricted Diet (Topic) or Carbohydrate Restricted Diet (Topic) or Carbohydrate-Restricted Diets (Topic) or Diets, Carbohydrate-Restricted (Topic) or Low-Carbohydrate Diet (Topic) or Diet, Low-Carbohydrate (Topic) or Diets, Low-Carbohydrate (Topic) or Low Carbohydrate Diet (Topic) or Low-Carbohydrate Diets (Topic)
5. Diet, Fat-Restricted (Topic) or Diet, Fat Restricted (Topic) or Fat-Restricted Diet (Topic) or Diets, Fat-Restricted (Topic) or Fat Restricted Diet (Topic) or Fat-Restricted Diets (Topic) or Diet, Low-Fat (Topic) or Diet, Low Fat (Topic) or Diets, Low-Fat (Topic) or Low-Fat Diet (Topic) or Low-Fat Diets (Topic) or Diet, Fat-Free (Topic) or Diet, Fat Free (Topic) or Diets, Fat-Free (Topic) or Fat-Free Diet (Topic) or Fat-Free Diets (Topic)
6. 3 and 4 and 5
7. diet,high-protein (Topic) or diet,high protein (Topic) or diets,high-protein (Topic) or high-protein diets (Topic) or high-protein diet (Topic) or high protein diet (Topic)
8. 3 and 4 and 7
9. 3 and 5 and 7
10. 6 or 8 or 9
11. Add filter: Language: English, Document type: Article
12. randomized controlled trial (All Fields) or pragmatic clinical trial (All Fields)
13. 1970-01-01/2023-09-30 (Publication Date)

14. 10 and 11 and 12 and 13

Cochrane Central Register of Controlled Trials (CENTRAL)

1. MeSH descriptor: [Blood Pressure] explode all trees
2. Blood Pressure or Pressure, Blood or Diastolic Pressure or Pressure, Diastolic or Pulse Pressure or Pressure, Pulse or Systolic Pressure or Pressure, Systolic or Pressures, Systolic
3. 1 or 2
4. MeSH descriptor: [Blood Glucose] explode all trees
5. Blood Glucose or Blood Sugar or Sugar, Blood or Glucose, Blood
6. 4 or 5
7. 3 or 6
8. MeSH descriptor: [Diet, Carbohydrate-Restricted] explode all trees
9. (Diet, Carbohydrate-Restricted or Diet, Carbohydrate Restricted or Diet, Low Carbohydrate or Carbohydrate Diet, Low or Carbohydrate Diets, Low or Diets, Low Carbohydrate or Low Carbohydrate Diets or Carbohydrate-Restricted Diet or Carbohydrate Restricted Diet or Carbohydrate-Restricted Diets or Diets, Carbohydrate-Restricted or Low-Carbohydrate Diet or Diet, Low-Carbohydrate or Diets, Low-Carbohydrate or Low Carbohydrate Diet or Low-Carbohydrate Diets)
10. 8 or 9
11. MeSH descriptor: [Diet, Fat-Restricted] explode all trees
12. (Diet, Fat-Restricted or Diet, Fat Restricted or Fat-Restricted Diet or Diets, Fat-Restricted or Fat Restricted Diet or Fat-Restricted Diets or Diet, Low-Fat or Diet, Low Fat or Diets, Low-Fat or Low-Fat Diet or Low-Fat Diets or Diet, Fat-Free or Diet, Fat Free or Diets, Fat-Free or Fat-Free Diet or Fat-Free Diets)
13. 11 or 12
14. 7 and 10 and 13
15. MeSH descriptor: [Diet, High-Protein] explode all trees
16. (diet,high-protein or diet,high protein or diets,high-protein or high-protein diets or high-protein diet or high protein diet)
17. 15 or 16
18. 7 and 10 and 17
19. 7 and 13 and 17
20. 14 or 18 or 19

3.Blood lipids

PUBMED

1. Cholesterol[Mesh] OR (Epicholesterol OR Triglycerides [Mesh] OR Triacylglycerols OR Triacylglycerol OR Triglyceride OR blood lipids)
2. Diet, Carbohydrate-Restricted[Mesh] OR (Diet, Carbohydrate Restricted OR Diet, Low Carbohydrate OR Carbohydrate Diet, Low OR Carbohydrate Diets, Low OR Diets, Low Carbohydrate OR Low Carbohydrate Diets OR Carbohydrate-Restricted Diet OR Carbohydrate Restricted Diet OR Carbohydrate-Restricted Diets OR Diets, Carbohydrate-Restricted OR Low-Carbohydrate Diet OR Diet, Low-Carbohydrate OR Diets, Low-Carbohydrate OR Low Carbohydrate Diet OR Low-Carbohydrate Diets)

3. Diet, Fat-Restricted[Mesh]) OR (Diet, Fat Restricted OR Fat-Restricted Diet OR Diets, Fat-Restricted OR Fat Restricted Diet OR Fat-Restricted Diets OR Diet, Low-Fat OR Diet, Low Fat OR Diets, Low-Fat OR Low-Fat Diet OR Low-Fat Diets OR Diet, Fat-Free OR Diet, Fat Free OR Diets, Fat-Free OR Fat-Free Diet OR Fat-Free Diets)
4. 1 AND 2 AND 3
5. Diet, High-Protein[Mesh] OR (Diet, High Protein OR Diets, High-Protein OR High-Protein Diets OR High-Protein Diet OR High Protein Diet)
6. 1 AND 2 AND 5
7. A AND 3 AND 5
8. 4 OR 6 OR 7
9. (randomized controlled trial [Publication Type]) OR (controlled clinical trial [Publication Type]) OR ("clinical trials as topic" [Mesh]) OR (randomized [Title/Abstract]) OR (randomly [Title/Abstract]) OR (random [Title/Abstract]) OR (trial [Title/Abstract])
10. ("1960/1/1"[Date - Publication] : "2023/9"[Date - Publication])
11. 8 AND 9 AND 10
12. ("animals" [Mesh]) NOT ("humans" [Mesh])
13. 11 NOT 12

EMBASE
1. (cholesterol or Epicholesterol or Triglycerides or Triacylglycerols or Triacylglycerol or Triglyceride or Blood lipids).af.
2. (Diet, Carbohydrate-Restricted* or low carbohydrate or low carbohydrate diet or low carbohydrate or carbohydrate restructed).af.
3. (Diet, Fat-Restricted* or diet, low fat or low fat diet or low fat or fat restricted).af.
4. 1 and 2 and 3
5. (diet,high-protein or diet,high protein or diets,high-protein or high-protein diets or high-protein diet or high protein diet).af.
6. 1 and 2 and 5
7. A and 3 and 5
8. 4 or 6 or 7
9. English.lg
10. 8 and 9
11. Add filters for: Publication Year: 1947-2023, subject: clinical trial, publication type: Article

Web of science search: selecting Web of Science Core Collection databases
1. cholesterol (Topic) or Epicholesterol (Topic) or Triglycerides (Topic) or Triacylglycerols (Topic) or Triacylglycerol (Topic) or Triglyceride (Topic) or Blood lipids (Topic)
2. Diet, Carbohydrate-Restricted (Topic) or Diet, Carbohydrate Restricted (Topic) or Diet, Low Carbohydrate (Topic) or Carbohydrate Diet, Low (Topic) or Carbohydrate Diets, Low (Topic) or Diets, Low Carbohydrate (Topic) or Low Carbohydrate Diets (Topic) or Carbohydrate-Restricted Diet (Topic) or Carbohydrate Restricted Diet (Topic) or Carbohydrate-Restricted Diets (Topic) or Diets, Carbohydrate-Restricted (Topic) or Low-Carbohydrate Diet (Topic) or Diet, Low-Carbohydrate (Topic) or Diets, Low-Carbohydrate (Topic) or Low Carbohydrate Diet (Topic) or Low-Carbohydrate Diets (Topic)

3. Diet, Fat-Restricted (Topic) or Diet, Fat Restricted (Topic) or Fat-Restricted Diet (Topic) or Diets, Fat-Restricted (Topic) or Fat Restricted Diet (Topic) or Fat-Restricted Diets (Topic) or Diet, Low-Fat (Topic) or Diet, Low Fat (Topic) or Diets, Low-Fat (Topic) or Low-Fat Diet (Topic) or Low-Fat Diets (Topic) or Diet, Fat-Free (Topic) or Diet, Fat Free (Topic) or Diets, Fat-Free (Topic) or Fat-Free Diet (Topic) or Fat-Free Diets (Topic)
4. 1 and 2 and 3
5. diet,high-protein (Topic) or diet,high protein (Topic) or diets,high-protein (Topic) or high-protein diets (Topic) or high-protein diet (Topic) or high protein diet (Topic)
6. 1 and 2 and 6
7. 1 and 3 and 6
8. 5 or 7 or 8
9. Add filter: Language: English, Document type: Article
10. randomized controlled trial (All Fields) or pragmatic clinical trial (All Fields)
11. 1970-01-01/2023-09-30 (Publication Date)
12. 8 and 9 and 10 and 11

Cochrane Central Register of Controlled Trials (CENTRAL)
1. MeSH descriptor: [Cholesterol] explode all trees
2. (cholesterol) OR (Epicholesterol)
3. 1 or 2
4. MeSH descriptor: [Diet, Carbohydrate-Restricted] explode all trees
5. Diet, Carbohydrate-Restricted or Diet, Carbohydrate Restricted or Diet, Low Carbohydrate or Carbohydrate Diet, Low or Carbohydrate Diets, Low or Diets, Low Carbohydrate or Low Carbohydrate Diets or Carbohydrate-Restricted Diet or Carbohydrate Restricted Diet or Carbohydrate-Restricted Diets or Diets, Carbohydrate-Restricted or Low-Carbohydrate Diet or Diet, Low-Carbohydrate or Diets, Low-Carbohydrate or Low Carbohydrate Diet or Low-Carbohydrate Diets
6. 4 or 5
7. MeSH descriptor: [Diet, Fat-Restricted] explode all trees
8. Diet, Fat-Restricted or Diet, Fat Restricted or Fat-Restricted Diet or Diets, Fat-Restricted or Fat Restricted Diet or Fat-Restricted Diets or Diet, Low-Fat or Diet, Low Fat or Diets, Low-Fat or Low-Fat Diet or Low-Fat Diets or Diet, Fat-Free or Diet, Fat Free or Diets, Fat-Free or Fat-Free Diet or Fat-Free Diets
9. 7 or 8
10. 3 and 6 and 9
11. MeSH descriptor: [Diet, High-Protein] explode all trees
12. diet,high-protein or diet,high protein or diets,high-protein or high-protein diets or high-protein diet or high protein diet
13. 7 or 8
14. 3 and 6 and 9
15. 3 and 9 and 13
16. 10 or 14 or 15

Appendix S2:Search update (November, 2024)

The same search strategy used in the original search was used, along with the same limitation strategy used in the first update, but with the new date range being from October 1, 2023 to November 30, 2024.

Table S1: List of trials excluded from the analyses.

1.Duplicates (n= 1trial) [1]
2.Not sufficient information (n=9 trials) [2-10]
3.Not relevant intervention (n=193 trials) [11-203]
4.Not clear interventions (n=14 trials) [204-217]
5.Mismatch of interventions (n=31 trials) [218-248]
6.Shorter than 1 weeks (n=3 trials) [249-251]
7.Not relevant result (n=9 trials) [252-260]
8.Incomplete result (n=5 trials) [261-265]
9.Non-English Articles (n=2 trials) [266-267]
10.Review (n=18 trials) [268-285]
11. Non randomized trials (n=1 trial) [286]

Table S2: Characteristics of included studies.

Study	Population description	Age	country	Network comparisons	Non-dietary components of intervention	During time (weeks)	Funding
William, 2004 [1]	overweight, hyperlipidemic volunteers with a BMI of 30-60 kg/m^2 (n=120)	18 to 65	USA	very Low carbohydrate-low protein diet VS. moderate fat-low protein diet	physical activity	24	non-industry funding
Camilla, 2023 [2]	T2DM participants with mean BMI of 33 kg/m^2 (n=165)	57 ± 9	Denmark	moderate carbohydrate-low protein diet VS. moderate fat-low protein diet	No	36	industry-funding
Jeannie, 2018 [3]	T2DM adults with mean BMI of 34.6 kg/m^2 (n=115)	35 to 68	Australia	moderate carbohydrate-low protein diet VS. moderate fat-low protein diet	—	96	non-industry funding
Huilan, 2022 [4]	obese/overweight adults with IGR, BMI of 24-35 kg/m^2 (n=90)	30 to 65	China	moderate carbohydrate-high protein diet VS. moderate fat-low protein diet	calorie restriction; physical activity	10	non-industry funding
Guldbrand, 2012 [5]	adults with mean BMI of 33.8 kg/m^2 (n=61)	—	Sweden	moderate carbohydrate-high protein diet VS. moderate fat-low protein diet	calorie restriction	96	non-industry funding
Lim, 2009 [6]	adults with mean BMI of 32 kg/m^2 (n=113)	20 to 65	Australia	very low carbohydrate-high protein diet VS. very low fat-low protein diet VS. moderate fat-low protein diet	calorie restriction	60	non-industry funding
Jeannie, 2015 [7]	obese adults with mean BMI of 34.6 kg/m^2 (n=115)	58 ± 7	Australia	moderate carbohydrate-low protein diet VS. moderate fat-low protein diet	calorie restriction; physical activity	52	non-industry funding
Thomas, 2014 [8]	adults with mean BMI of 33.6 kg/m^2 (n=43)	49.2 ± 1.1	Australia	very low carbohydrate-high protein diet VS. moderate fat-low protein diet	calorie restriction	52	not reported
Grant, 2009 [9]	obese adults with mean BMI of 33.6 kg/m^2 (n=69)	—	Australia	very low carbohydrate-high protein diet VS. moderate fat-low protein diet	calorie restriction	48	industry-funding
Brinkworth, 2016 [10]	obese adults with mean BMI of 34.6 kg/m^2 (n=115)	—	Australia	moderate carbohydrate-low protein diet VS. moderate fat-low protein diet	calorie restriction; physical activity	52	non-industry funding
Jeannie, 2014 [11]	obese adults with mean BMI of 34.4 kg/m^2 (n=115)	58 ± 7	Australia	moderate carbohydrate-low protein diet VS. moderate fat-low protein diet	calorie restriction; physical activity	24	non-industry funding
Wycherley, 2010 [12]	adults with mean BMI of 33.7 kg/m^2 (n=49)	50 ± 1.1	Australia	very low carbohydrate-high protein diet VS. moderate fat-low protein diet	calorie restriction	52	industry-funding
Una, 2009 [13]	adults with mean BMI of 33.6 kg/m^2 (n=24)	39 ± 10	U.K.	moderate carbohydrate-low protein diet VS. moderate fat-low protein diet	calorie restriction	8	non-industry funding
Jeannie, 2008 [14]	obese adults with mean BMI of 33.9 kg/m^2 (n=88)	18 to 65	Australia	very low carbohydrate-high protein diet VS. moderate fat-low protein diet	calorie restriction	24	industry-funding
Guldbrand, 2014 [15]	adults with Type 2 diabetes mellitus with mean BMI of 32.7 kg/m^2 (n=61)	61.2 ± 9.5	Sweden	moderate carbohydrate-high protein diet VS. moderate fat-low protein diet	calorie restriction	96	non-industry funding
Stijn, 2012 [16]	adults with 37 kg/m^2 (n=132)	50 ± 12	Netherlands	moderate carbohydrate-low protein diet VS. moderate fat-low protein diet	—	36	non-industry funding
Grant, 2010 [17]	obese adults with mean	24 to 64	Australia	very low carbohydrate-high protein	calorie	54	industry-funding

	BMI of 33.6 kg/m^2 (n=68)			diet VS. moderate fat-low protein diet	restriction		
Colene, 2008 [18]	adults with 34.5 kg/m^2 (n=28)	57 ± 9.8	USA	moderate carbohydrate-low protein diet VS. moderate fat-low protein diet	—	8	not reported
Alain, 1996 [19]	obese adults with a BMI ＞30 kg/m^2 (n=43)	41 ± 9	USA	moderate carbohydrate-high protein diet VS. moderate fat-low protein diet	calorie restriction; physical activity	6	not reported
Stephen, 2009 [20]	adults with atherogenic dyslipidemia with a BMI ＞25 kg/m^2 (n=40)	18 to 55	USA	moderate carbohydrate-low protein diet VS. moderate fat-low protein diet	calorie restriction	12	non-industry funding
Noor, 2020 [21]	obese adults with type 2 diabetes with mean BMI of 34.5 kg/m^2 (n=84)	35 to 68	Australia	moderate carbohydrate-low protein diet VS. moderate fat-low protein diet	calorie restriction; physical activity	16	non-industry funding
Charles, 2010 [22]	adults with a BMI ≥30 kg/m^2 (n=16)	—	USA	moderate carbohydrate-low protein diet VS. moderate fat-low protein diet	calorie restriction	20	not reported
Manny, 2006 [23]	adults with mean BMI of 33 kg/m^2 (n=83)	48 ± 8	Australia	very low carbohydrate-high protein diet VS. very low fat-low protein diet VS. moderate fat-low protein diet	calorie restriction	8	non-industry funding
Jeannie Tay, 2015[24]	T2D adults with mean BMI of 34.6 kg/m^2 (n=115)	35 to 68	Australia	moderate carbohydrate-low protein diet VS. moderate fat-low protein diet	calorie restriction; physical activity	52	non-industry funding
Megan, 2014[25]	obese adults with a BMI of 29-44.6 kg/m^2 (n=55)	21 to 62	USA	very low carbohydrate-high protein diet VS. moderate fat-low protein diet	—	12	non-industry funding
Jia, 2022[26]	obese women with a BMI of 35.4 kg/m^2 (n=121)	41.2 ± 10.2	New Zealand	moderate carbohydrate-high protein diet VS. very low-fat-low-protein diet VS. moderate fat-high protein diet	—	8	non-industry funding
Mcauley, 2004[27]	obese women with a BMI ＞ 27 kg/m^2 (n=96)	—	New Zealand	moderate fat-high protein diet VS. moderate fat-low protein diet	physical activity	24	non-industry funding
Jenkins, 2014[28]	obese adults with a BMI ＞ 27 kg/m^2 (n=39)	55.3 ± 1.8	Canada	moderate carbohydrate-high protein diet VS. moderate fat-low protein diet	—	24	industry-funding
Morenga, 2010[29]	adults with a BMI ≥27 kg/m^2 (n=83)	18 to 65	New Zealand	moderate fat-high protein diet VS. moderate fat-low protein diet	—	10	industry-funding
Grant D, 2009[30]	obese adults with mean BMI of 33.6 kg/m^2 (n=60)	49.2 ± 1.2	Australia	very low carbohydrate-high protein diet VS. moderate fat-low protein diet	calorie restriction; physical activity	8	industry-funding
Brinkworth, 2009[31]	adults with mean BMI of 33.7 kg/m^2 (n=106)	24 to 64	Australia	very low carbohydrate-high protein diet VS. moderate fat-low protein diet	calorie restriction	52	industry-funding
Angela, 2007[32]	obese adults with mean BMI of 33.6 kg/m^2 (n=93)	24 to 64	Australia	very low carbohydrate-high protein diet VS. moderate fat-low protein diet	calorie restriction	8	non-industry funding
Thomas, 2010[33]	adults with type 2 diabetes with mean BMI of 35.4 kg/m^2 (n=83)	56.1 ± 7.5	Australia	moderate fat-high protein diet VS. moderate fat-low protein diet	calorie restriction; physical activity	16	industry-funding
Natalie, 2005[34]	adults with mean BMI of 33.8 kg/m^2 (n=57)	—	Australia	moderate fat-high protein diet VS. moderate carbohydrate-low protein diet	calorie restriction; physical activity	16	non-industry funding

Thomas, 2013[35]	men with type 2 diabetes with mean BMI of 33.6 kg/m^2 (n=56)	45.5 ± 8.7	Australia	moderate fat-high protein diet VS. moderate fat-low protein diet	calorie restriction	12	non-industry funding
Manny, 2005[36]	adults with mean BMI of 32 kg/m^2 (n=133)	20 to 65	Australia	moderate fat-high protein diet VS. moderate fat-low protein diet	calorie restriction; physical activity	12	non-industry funding
Victoria, 2020[37]	adults with mean BMI of 32.8 kg/m^2 (n=67)	55.6 ± 8.37	Spain	moderate fat-high protein diet VS. moderate fat-low protein diet	—	12	non-industry funding
Carol, 2004[38]	healthy adults with mean BMI of 29.1 kg/m^2 (n=17)	—	USA	moderate fat-high protein diet VS. moderate fat-low protein diet	calorie restriction	6	non-industry funding
Krebs, 2012[39]	adults with a BMI ＞ 27 kg/m^2 (n=419)	30 to 75	New Zealand	moderate fat-high protein diet VS. moderate fat-low protein diet	calorie restriction; physical activity	96	non-industry funding
Sargrad, 2005[40]	patients with type 2 diabetes with mean BMI of 36 kg/m^2 (n=12)	47 ± 1.9	USA	moderate fat-high protein diet VS. moderate fat-low protein diet	—	8	non-industry funding
Kleiner, 2006[41]	adults with a BMI ≥ 25 kg/m^2 (n=16)	34.6 ± 3.6	USA	moderate fat-high protein diet VS. moderate fat-low protein diet	calorie restriction	8	non-industry funding
Riccardo, 2013[42]	adults with a BMI ＞ 40 kg/m^2 (n=88)	19 to 65	Italy	moderate fat-high protein diet VS. moderate fat-low protein diet	calorie restriction	48	not reported
Griffin, 2013[43]	adults with a BMI ＞ 27.5 kg/m^2 (n=71)	18 to 25	Australia	moderate fat-high protein diet VS. moderate fat-low protein diet	calorie restriction; physical activity	48	non-industry funding
Nerylee, 2016[44]	T2DM adults with mean BMI of 34.3 kg/m^2 (n=61)	55 ± 8	Australia	moderate fat-high protein diet VS. moderate fat-low protein diet	calorie restriction; physical activity	12	industry-funding
Wycherley, 2012[45]	men with a BMI ＞ 33 kg/m^2 (n=68)	50.8 ± 9.3	Australia	moderate fat-high protein diet VS. moderate fat-low protein diet	calorie restriction	52	non-industry funding
Gannon, 2004[46]	adults with a BMI of 27 to 36 kg/m^2 (n=8)	51 to 82	USA	moderate carbohydrate-high protein diet VS. moderate fat-low protein diet	—	5	non-industry funding
Nour, 2023[47]	health women with a BMI ≥ 25 kg/m^2 (n=40)	20 to 50	Jordan	very low carbohydrate-low protein diet VS. moderate fat-high protein diet	physical activity	8	not reported
Brinkworth, 2004[48]	obese adults with type 2 diabetes with a BMI of 27 to 40 kg/m^2 (n=66)	62.7 ± 1.8	Australia	moderate fat-low protein diet VS. moderate fat-high protein diet	calorie restriction	12	industry-funding
Calleja, 2012[49]	adults with a BMI of 28 to 35 kg/m^2 (n=40)	18 to 70	España	moderate fat-high protein diet VS. moderate fat-low protein diet	—	48	not reported
Evangelista, 2021[50]	adults with overweight or obese (n=76)	57.7 ± 9.7	USA	moderate fat-high protein diet VS. moderate fat-low protein diet	calorie restriction; physical activity	12	non-industry funding
Goss, 2020[51]	obese adults with a BMI of 30 to 40 kg/m^2 (n=40)	60 to 75	USA	Very low carbohydrate-low protein diet VS. moderate fat-low protein diet	—	8	non-industry funding
Gallego, 2017[52]	adults with a BMI of 27.5 to 45 kg/m^2 (n=91)	18 to 80	Spain	moderate fat-low protein diet VS. moderate fat-high protein diet	—	12	non-industry funding
Nielsen, 2005[53]	obese adults with type 2 diabetes with a BMI ≥30 kg/m^2 (n=31)	—	sweden	moderate carbohydrate-high protein diet VS. moderate fat-low protein diet	calorie restriction	24	non-industry funding
Pedersen, 2013[54]	obese adults with type 2 diabetes with a BMI ≥27 kg/m^2 (n=45)	18 to 75	Australia	moderate fat-high protein diet VS. moderate fat-low protein diet	calorie restriction	48	not reported

Segal, 2004[55]	obese adults with a BMI of 28 to 40 kg/m^2 (n=4)	52.3 ± 3.8	UAS	very low carbohydrate-high protein diet VS. moderate fat-high protein diet	calorie restriction	12	industry-funding
Brinkworth, 2003[56]	obese adults with mean BMI of 34 kg/m^2 (n=43)	20 to 65	Australia	moderate fat-low protein diet VS. moderate fat-high protein diet	calorie restriction	68	industry-funding
Nadine, 2022[57]	adults with mean BMI of 22.1 kg/m^2 (n=24)	25.8 ± 3.7	Germany	very low fat-low protein diet VS. moderate carbohydrate-low protein diet	calorie restriction	3	not reported
Kelly, 2004[58]	health adults with a BMI ＞ 25 kg/m^2 (n=31)	24 to 61	Canada	moderate fat-high protein diet VS. moderate carbohydrate-high protein diet	calorie restriction	10	non-industry funding
Jeff, 2004[59]	Men with mean BMI of 29.6 kg/m^2 (n=13)	34 ± 8.6	USA	moderate fat-low protein diet VS. very low-carbohydrate-high protein diet	calorie restriction	4	non-industry funding
Lisa, 2011 [60]	obese women with a BMI ＞ 27 kg/m^2 (n=83)	18 to 65	New Zealand	moderate fat-high protein diet VS. moderate fat-low protein diet	calorie restriction; physical activity	8	industry-funding
Lewis, 1977 [61]	obese men (n=10)	18 to 28	USA	very low carbohydrate-low protein diet VS. very low fat-low protein diet	calorie restriction	2	non-industry funding
Alex, 2022 [62]	men with a BMI ＞ 21.5 kg/m^2 (n=7)	18 to 45	USA	very low carbohydrate-low protein diet VS. moderate fat-low protein diet	—	6	non-industry funding
Itziar, 2009 [63]	obese men with mean BMI of 34 kg/m^2 (n=19)	36 ± 6	Spain	moderate fat-low protein diet VS. moderate fat-high protein diet	physical activity	8	not reported
Lisa, 2022 [64]	adults with a BMI ≥ 27 kg/m^2 (n=70)	18 to 50	USA	very low carbohydrate-low protein diet VS. moderate fat-low protein diet	physical activity	1	non-industry funding
Piatti, 1994 [65]	obese women with a BMI ＞ 38.2 kg/m^2 (n=25)	40 ± 3	Italy	moderate fat-high protein diet VS. moderate fat-low protein diet	—	3	not reported
Cara, 2022 [66]	Adults with mean BMI of 32.4 kg/m^2 (n=164)	18 to 65	USA	moderate carbohydrate-low protein diet VS. moderate fat-low protein diet	calorie restriction	20	non-industry funding

MFLP, moderate fat-low protein diet. MCHP, moderate carbohydrate-high protein diet. MCLP, moderate carbohydrate-low protein diet. MFHP, moderate fat-high protein diet. VLCHP, very low carbohydrate-high protein diet. VLCLP, very low carbohydrate-low protein diet. VLFHP, very low fat-low protein diet. VLFLP, very low fat-low protein diet. IGR, impaired glucose regulation. BMI, body-mass index

Table S3: Quality of trials included in the network meta-analysis of dietary groups.

Study (author, year)	Random Sequence Generation	Allocation concealment	Blinding of participants and personnel	Blinding of outcome assessment	Incomplete outcome data	Selective outcome reporting	Other sources of bias	Overall quality*
Alain, 1996	U	U	U	U	L	L	L	Moderate risk
Alex, 2022	H	U	U	L	L	L	L	Moderate risk
Angela, 2007	U	U	U	U	L	L	L	Moderate risk
Brinkworth, 2003	U	U	U	U	L	L	L	Moderate risk
Brinkworth, 2004	L	U	U	U	L	L	L	Low risk
Brinkworth, 2009	L	U	U	U	L	L	L	Low risk
Brinkworth, 2016	L	U	U	U	L	L	L	Low risk
Calleja, 2012	L	U	U	U	L	L	L	Low risk
Camilla, 2023	L	U	L	U	L	L	L	Low risk
Cara, 2022	U	U	U	U	L	L	L	Moderate risk
Carol, 2004	L	U	U	U	L	L	L	Low risk
Charles, 2010	L	U	U	U	L	L	L	Low risk
Colene, 2008	L	U	U	U	L	L	L	Low risk
Evangelista, 2021	L	U	U	U	L	L	L	Low risk
Gallego, 2017	L	U	U	U	L	L	L	Low risk
Gannon, 2004	H	U	U	H	L	L	L	High risk
Goss, 2020	L	U	L	U	L	L	L	Low risk
Grant D, 2009	L	U	U	U	L	L	L	Low risk
Grant, 2009	L	U	U	U	L	L	L	Low risk
Grant, 2010	L	U	U	U	L	L	L	Low risk
Griffin, 2013	U	U	U	L	L	L	L	Low risk
Guldbrand, 2012	L	U	U	U	L	L	L	Low risk
Guldbrand, 2014	L	U	U	U	L	L	L	Low risk
Huilan, 2022	U	U	U	U	L	L	L	Moderate risk
Itziar, 2009	H	U	U	U	L	L	L	Moderate risk
Jeannie Tay, 2015	U	U	U	U	L	L	U	Moderate risk
Jeannie, 2008	L	U	U	U	L	L	L	Low risk
Jeannie, 2014	L	L	U	U	L	L	U	Low risk

Supplementary Materials

Jeannie, 2015	L	L	U	U	L	L	L	Low risk
Jeannie, 2018	L	L	U	L	L	L	L	Low risk
Jeff, 2004	H	U	U	H	L	L	U	High risk
Jenkins, 2014	U	U	U	L	L	L	L	Low risk
Jia, 2022	L	U	U	U	L	L	L	Low risk
Kelly, 2004	U	U	U	U	L	L	U	Moderate risk
Kleiner, 2006	U	U	U	U	L	L	U	Moderate risk
Krebs, 2012	L	U	U	L	L	L	U	Low risk
Lewis, 1977	H	U	U	U	L	L	U	Moderate risk
Lim, 2009	U	U	U	U	L	L	U	Moderate risk
Lisa, 2022	U	U	U	U	L	L	U	Moderate risk
Manny, 2005	L	U	U	U	L	L	L	Low risk
Manny, 2006	L	U	U	U	L	L	L	Low risk
Mcauley, 2004	L	U	U	U	L	L	L	Low risk
Megan, 2014	L	U	U	U	L	L	L	Low risk
Morenga, 2010	L	L	U	L	L	L	L	Low risk
Morenga, 2010	L	L	U	U	L	L	L	Low risk
Nadine, 2022	H	U	U	U	L	L	L	Moderate risk
Natalie, 2005	L	U	U	U	L	L	L	Low risk
Nerylee, 2016	U	U	U	U	L	L	U	Moderate risk
Nielsen, 2005	L	U	U	U	L	L	L	Low risk
Noor, 2020	L	L	U	U	L	L	L	Low risk
Nour, 2023	L	U	U	U	L	L	L	Low risk
Pedersen, 2013	U	U	U	U	L	L	L	Moderate risk
Piatti, 1994	L	U	U	U	L	L	L	Low risk
Riccardo, 2013	L	U	U	U	L	L	L	Low risk
Sargrad, 2005	L	U	U	U	L	L	L	Low risk
Segal, 2004	H	U	U	U	L	L	L	Moderate risk
Stephen, 2009	U	U	U	U	L	L	U	Moderate risk
Stijn, 2012	L	U	U	U	L	L	L	Low risk
Thomas, 2010	L	U	U	U	L	L	L	Low risk
Thomas, 2013	U	U	U	U	L	L	L	Moderate risk

Thomas, 2014	L	U	U	U	L	L	L	Low risk
Una, 2009	L	U	U	U	L	L	L	Low risk
Victoria, 2020	U	U	U	U	L	L	U	Moderate risk
William, 2004	L	U	U	U	L	L	L	Low risk
Wycherley, 2010	L	U	U	U	L	L	L	Low risk
Wycherley, 2012	U	U	U	U	L	L	L	Moderate risk

U: unclear risk of bias; L: low risk of bias; H: high risk of bias.
*For overall quality, low risk: not item with high risk; moderate risk: only one item with high risk or no more than three items with unclear risk; high risk: two or more items with high risk.

Figure S1-2: Risk of bias summary for the 66 included trials.

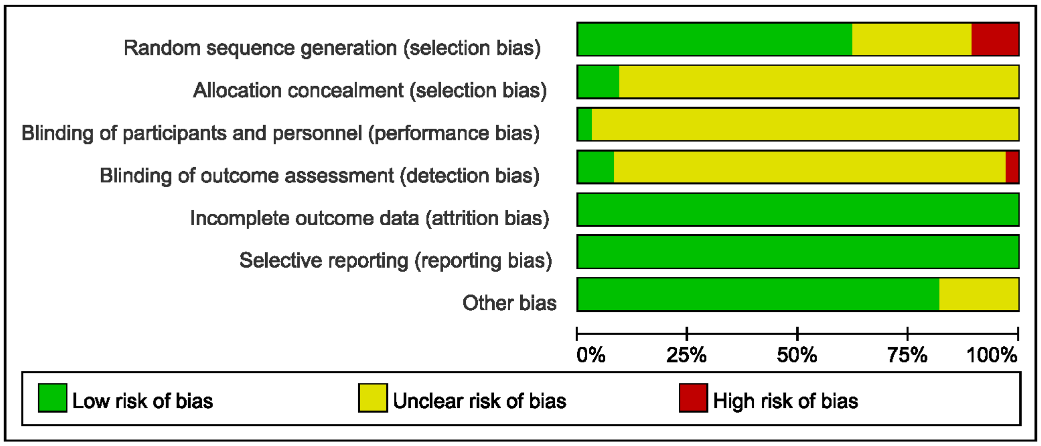

Fig.S1 Risk of bias graph for the 66 included trials

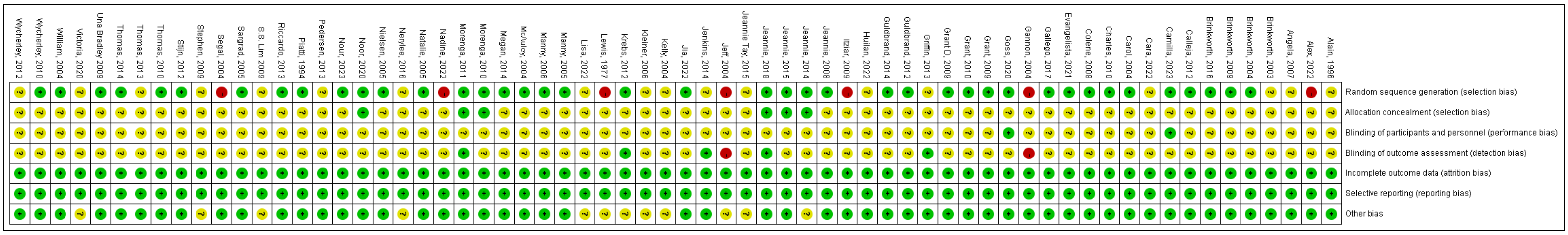

Figure S2 Risk of bias summary graph for the 66 included trials

Figure S3-S10: Network plots for each outcome.

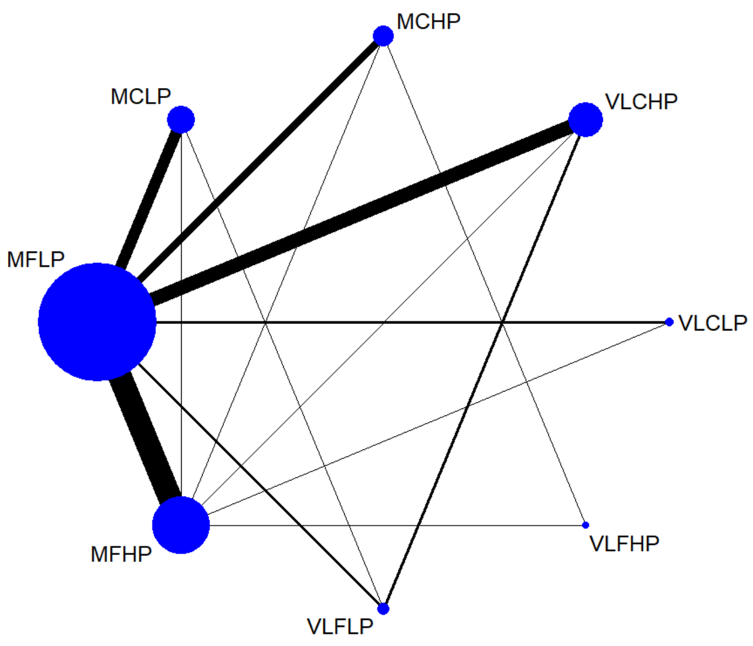

Fig.S3 Network plot of named dietary groups for weight change.

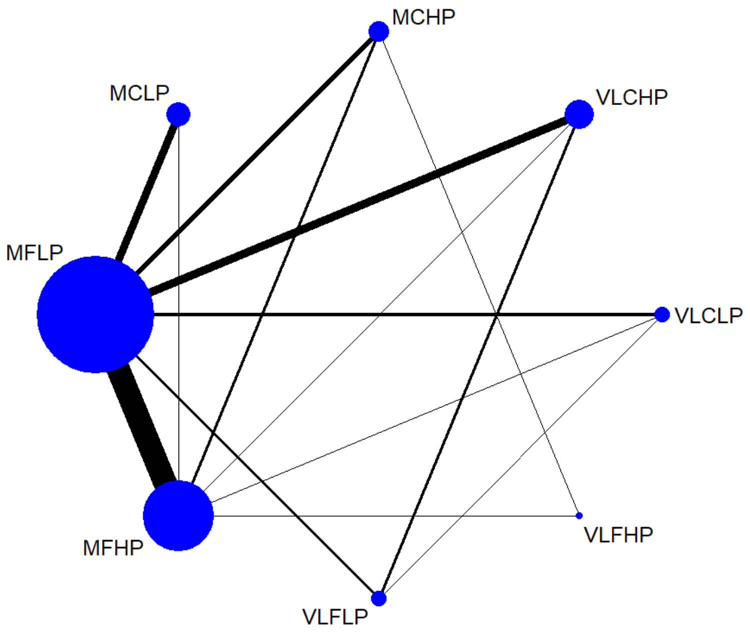

Fig.S4 Network plot of named dietary groups for blood glucose.

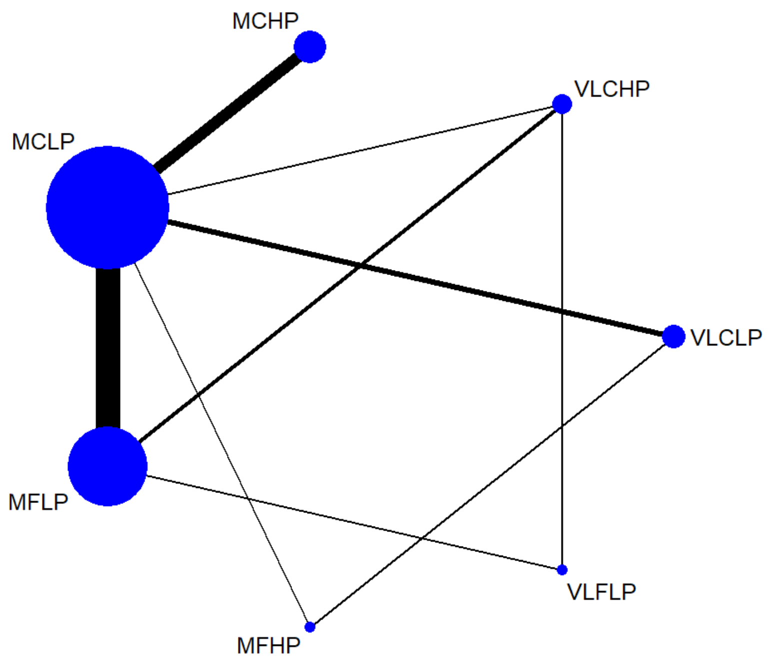

Fig.S5 Network plot of named dietary groups for systolic blood pressure.

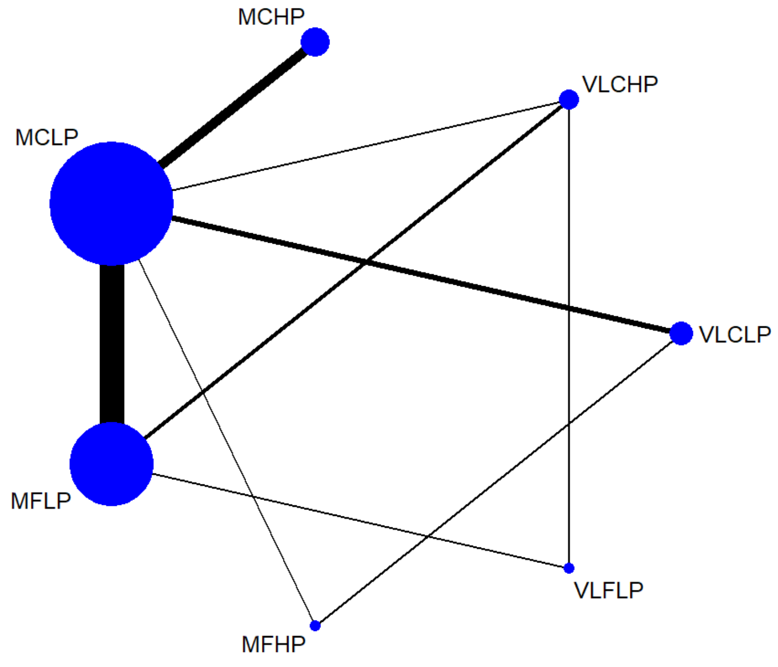

Fig.S6 Network plot of named dietary groups for diastolic blood pressure.

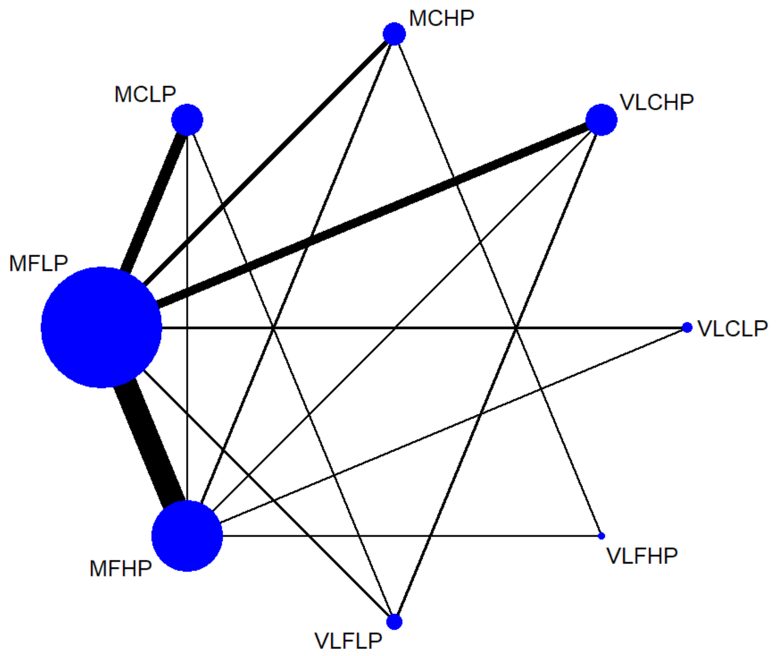

Fig.S7 Network plot of named dietary groups for HDL cholesterol.

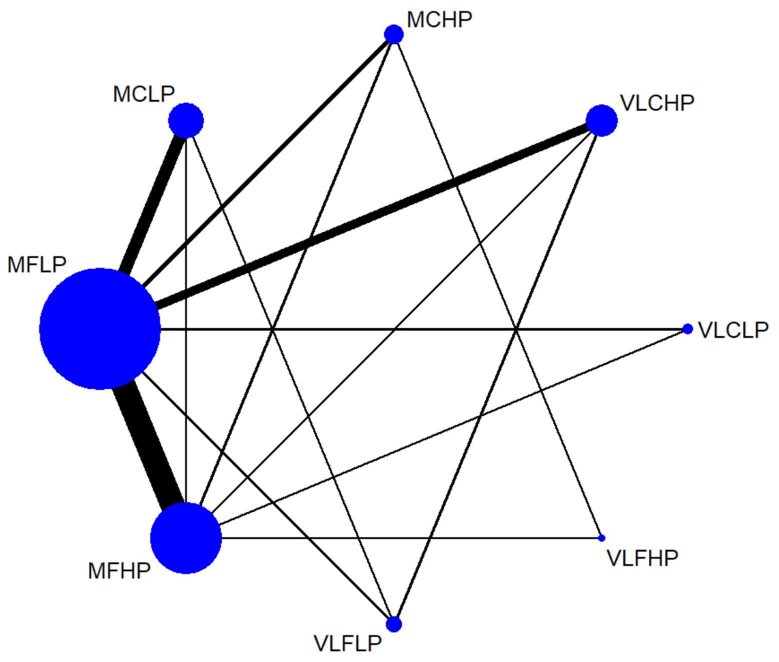

Fig.S8 Network plot of named dietary groups for LDL cholesterol.

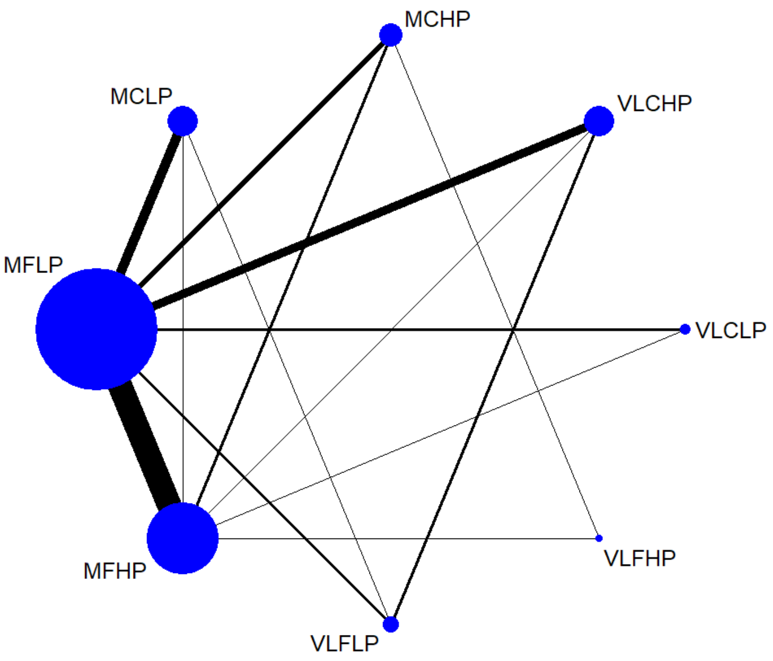

Fig.S9 Network plot of named dietary groups for triglyceride.

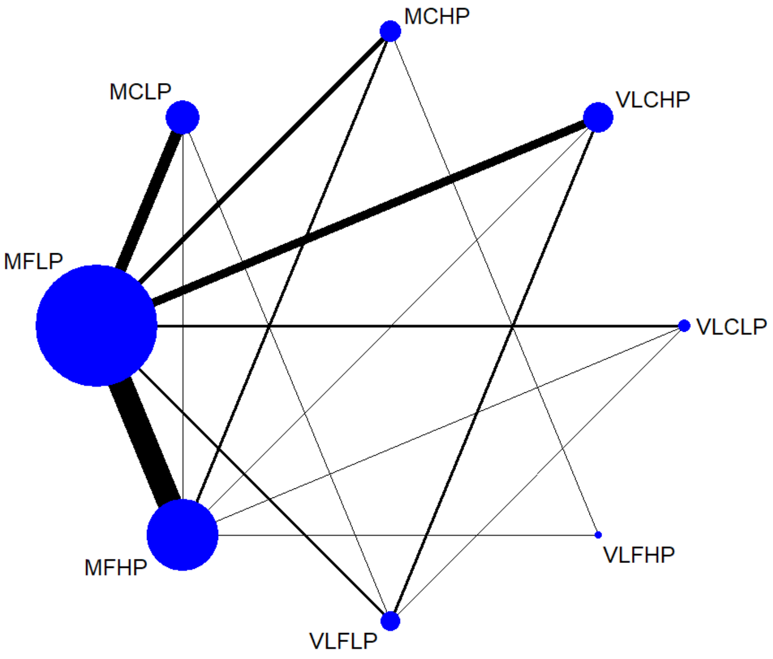

Fig.S10 Network plot of named dietary groups for total cholesterol.

Figure S11 Rank- heat plot of SUCRA for eight macronutrient dietary groups with all outcome indicators

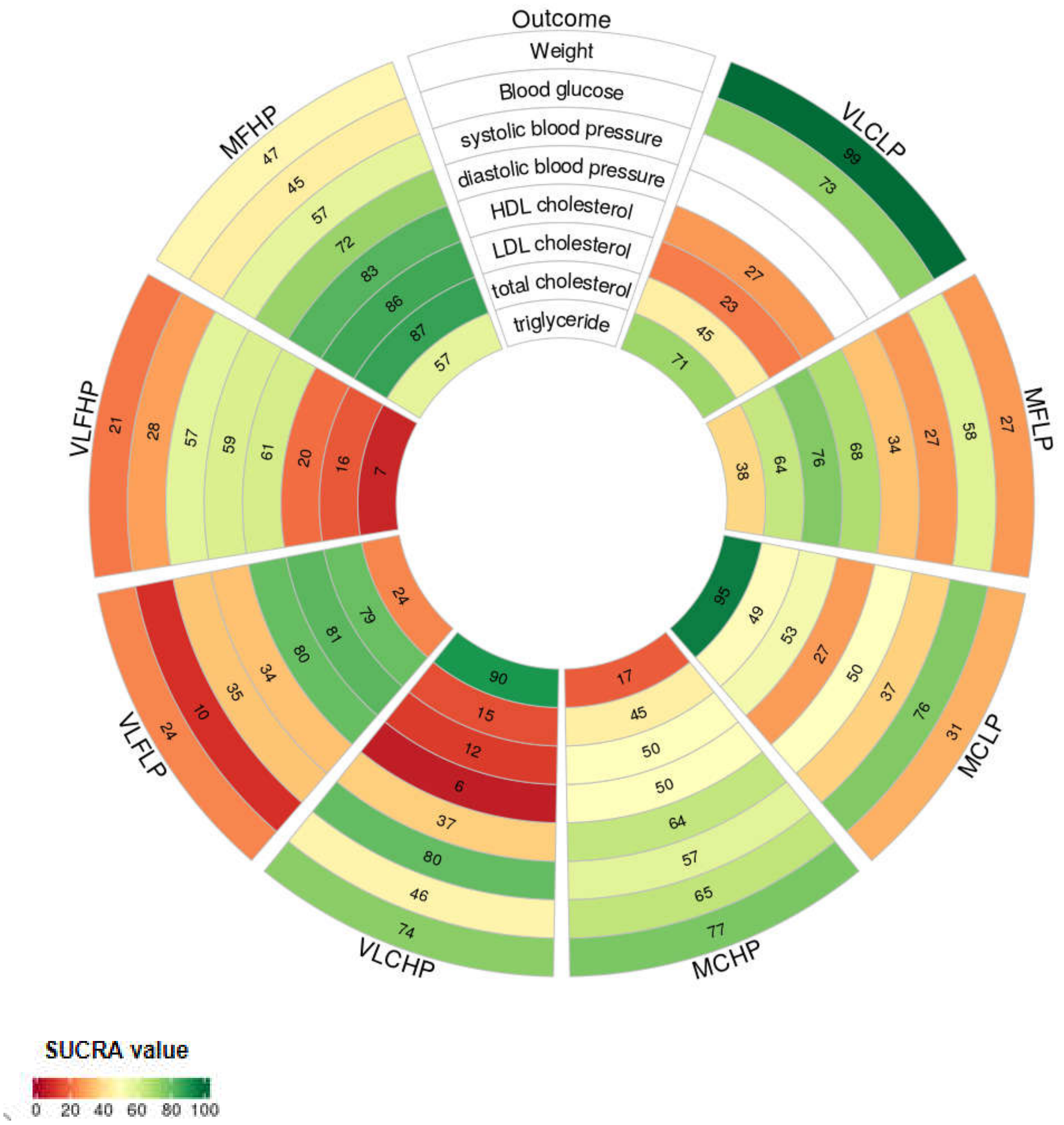

Fig.S11 Rank- heat plot of SUCRA for eight macronutrient dietary groups, including MFLP, MFHP, MCLP, MCHP, VLFLP, VLFHP, VLCLP, VLCHP with all outcome indicators. The blank spaces in the figure indicate that there were no studies on the VLCLP dietary group included in the original research for the outcomes of systolic and diastolic blood pressure. Circles from inside out present results for different network meta- analyses including: body weight, blood glucose, systolic and diastolic blood pressure, HDL cholesterol, LDL cholesterol, total cholesterol, and triglycerides, and total cholesterol. Numbers within each sector correspond to the SUCRA values as calculated in each model. Higher SUCRA values indicate that the dietary group ranked higher in terms of improving the corresponding outcome indicators. Abbreviations: MFLP, moderate fat-low protein; MFHP, moderate fat-high protein; MCLP, moderate carbohydrate-low protein; MCHP, moderate carbohydrate-high protein; VLFLP, very low fat-low protein; VLFHP, very low fat-high protein; VLCLP, very low carbohydrate-low protein; VLCHP, very low carbohydrate-high protein; HDL, high density lipoprotein; LDL, low density lipoprotein; SUCRA, SUCRA, surface under the curve cumulative ranking probabilities.

Table S4: Inconsistency test of network meta-analysis

Outcome	DIC of consistency model	DIC of unrelated mean effect model
Body weight	194.2	197
Blood glucose	185.5	189.8
Systolic blood pressure	97.9	99.1
Diastolic blood pressure	96.8	97.6
High density lipoprotein cholesterol	166.8	154.2
Low density lipoprotein cholesterol	173.2	173.9
Triglycerides	149.6	152.2
Total cholesterol	173.4	171.1

The difference in DIC values between consistency model and unrelated mean effect model is within 5 suggesting significant agreement between direct and indirect evidence

Figure S12-S19: Global inconsistency test chart for each outcome.

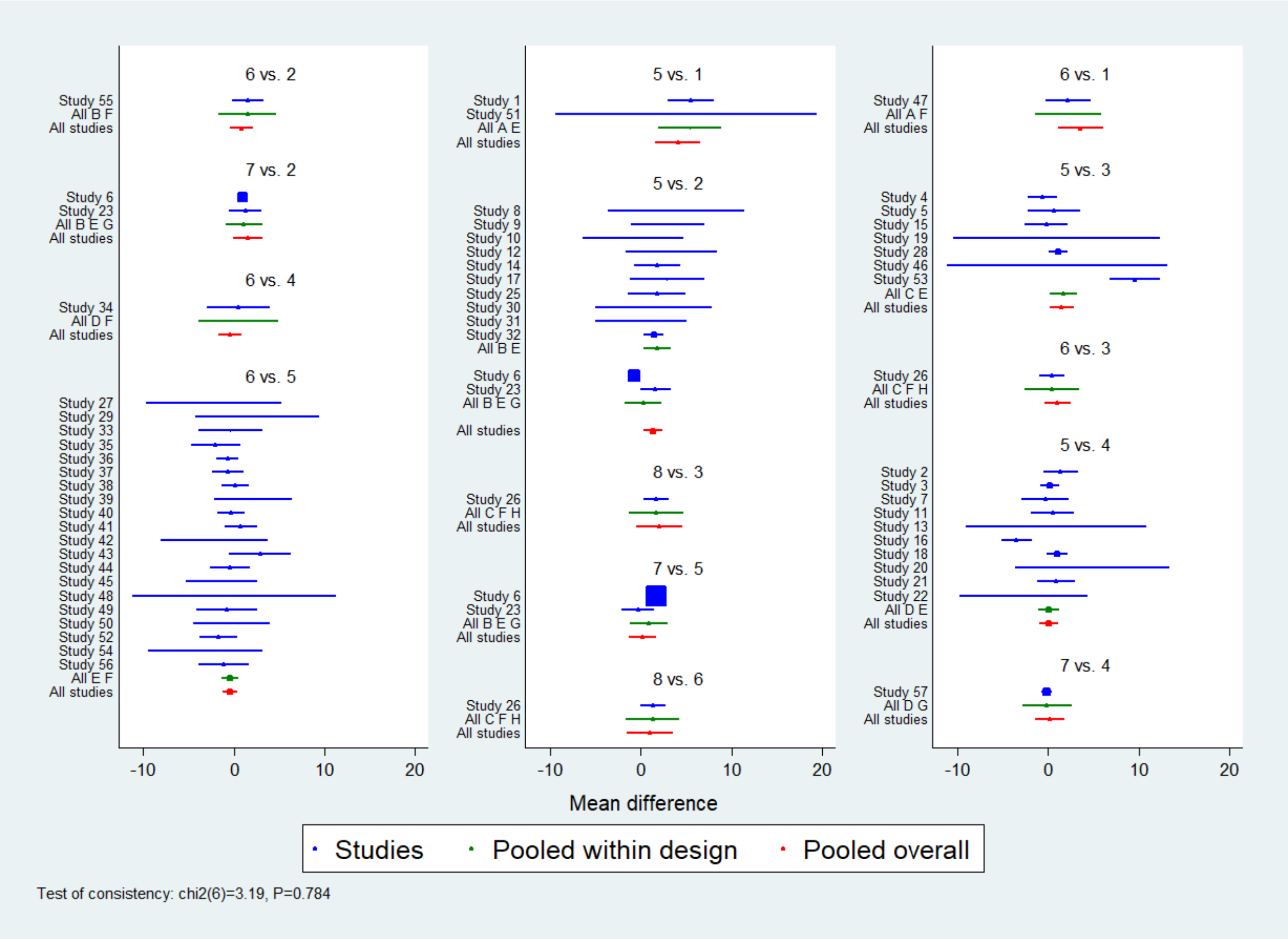

Figure S12 Inconsistency test chart of weight change. P > 0.05: Significant consistency between direct and circumstantial evidence. 1: VLCLP; 2: VLCHP; 3: MCHP; 4: MCLP; 5: MFLP; 6: MFHP; 7: VLFLP; 8: VLFHP.

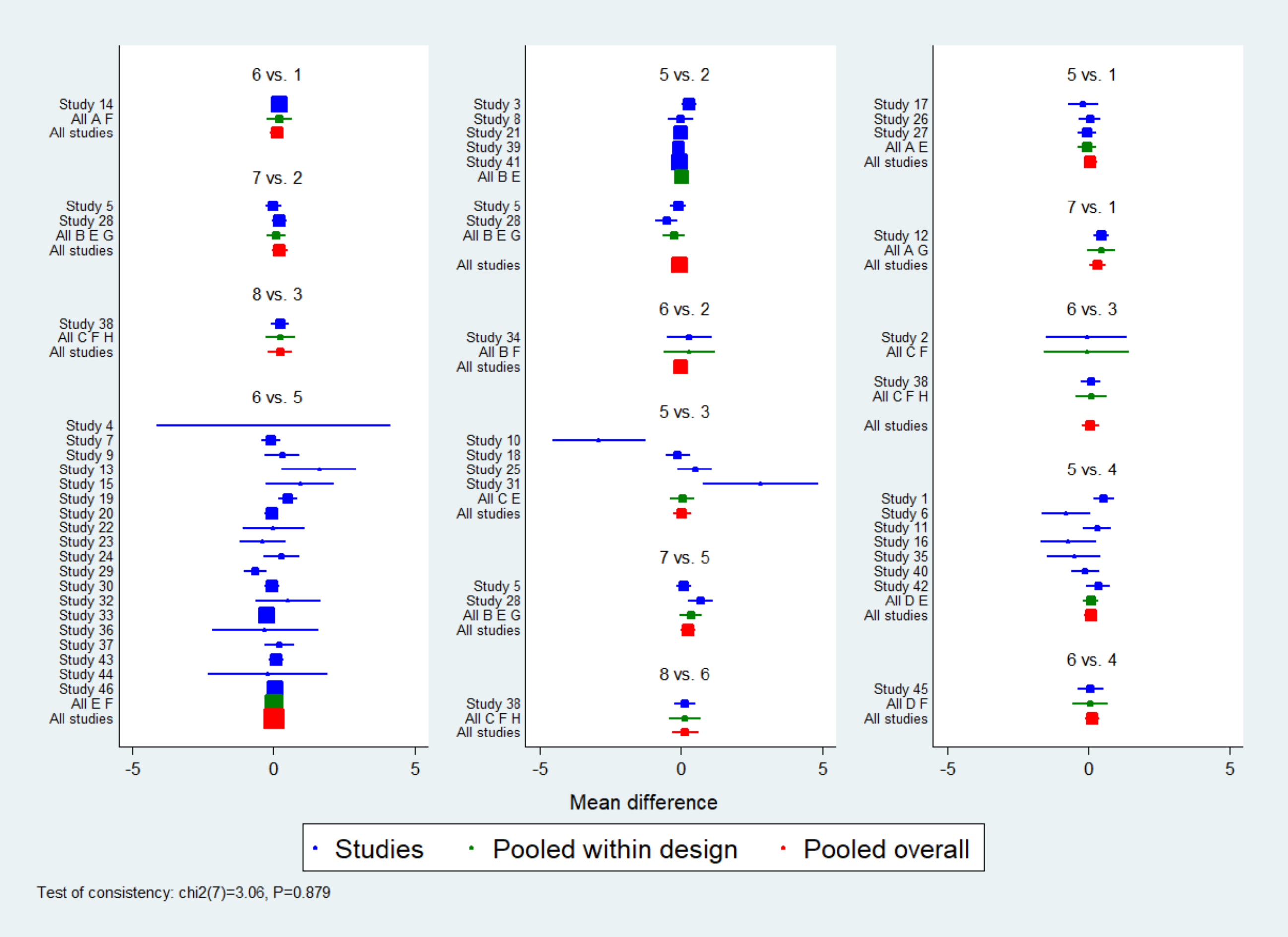

Figure S13 Inconsistency test chart of blood glucose. P > 0.05: Significant consistency between direct and circumstantial evidence. 1: VLCLP; 2: VLCHP; 3: MCHP; 4: MCLP; 5: MFLP; 6: MFHP; 7: VLFLP; 8: VLFHP.

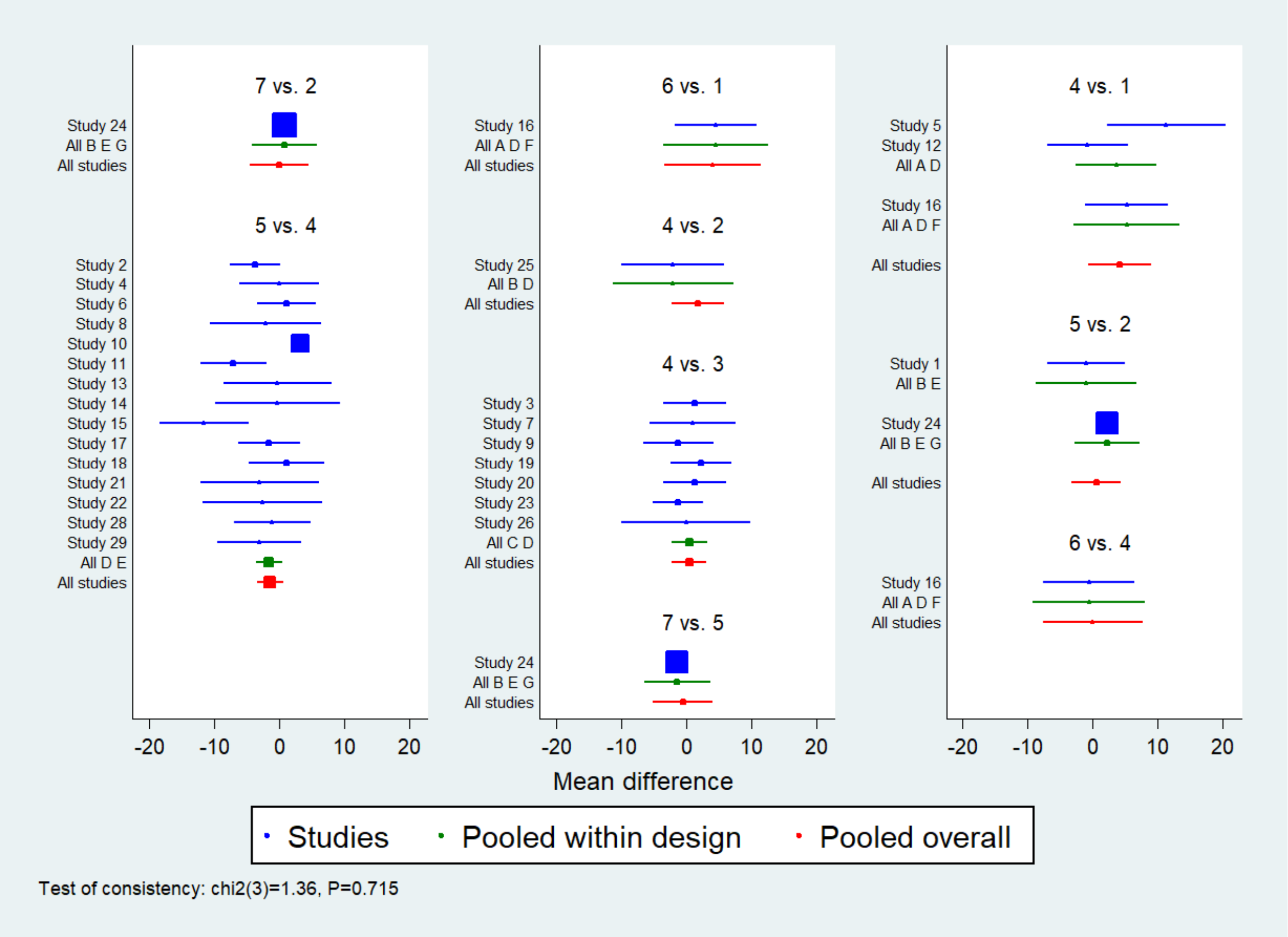

Figure S14 Inconsistency test chart of systolic blood pressure. P > 0.05: Significant consistency between direct and circumstantial evidence. 1: VLCHP; 2: MCHP; 3: MCLP; 4: MFLP; 5: MFHP; 6: VLFLP; 7: VLFHP.

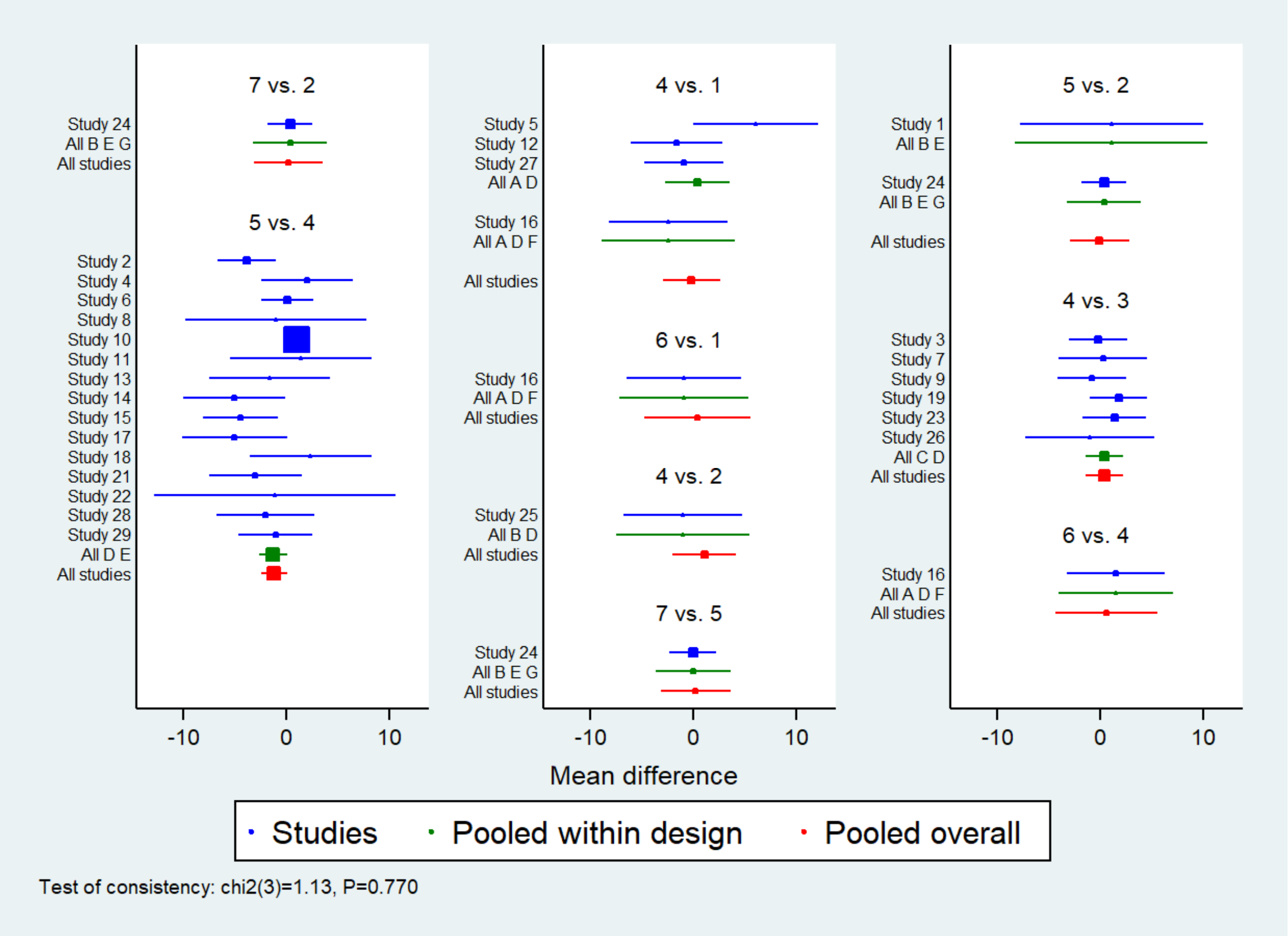

Figure S15 Inconsistency test chart of diastolic blood pressure. P > 0.05: Significant consistency between direct and circumstantial evidence. 1: VLCHP; 2: MCHP; 3: MCLP; 4: MFLP; 5: MFHP; 6: VLFLP; 7: VLFHP.

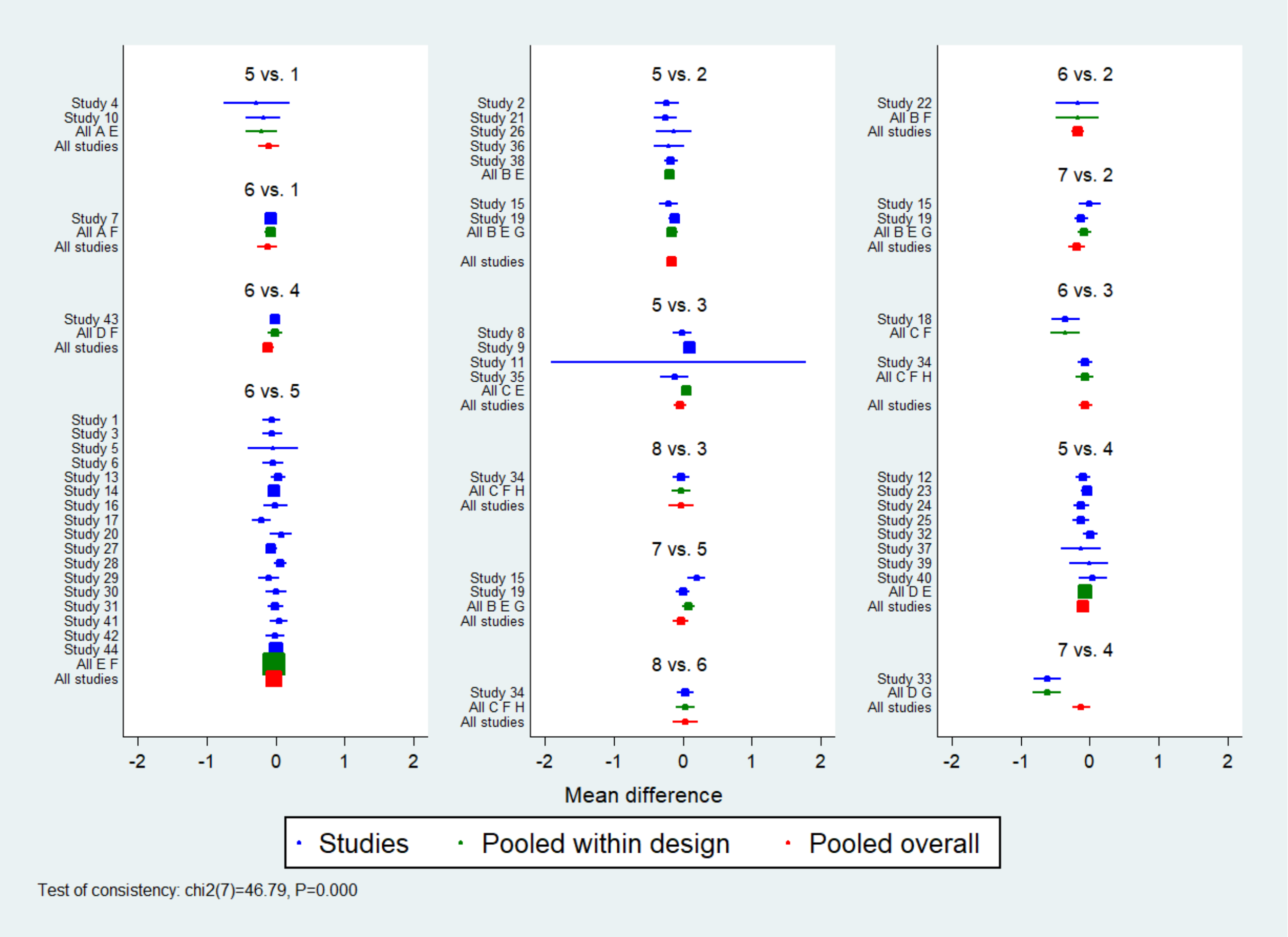

Figure S16 Inconsistency test chart of HDL cholesterol. P ＜ 0.05: No significant consistency between direct and circumstantial evidence. 1: VLCLP; 2: VLCHP; 3: MCHP; 4: MCLP; 5: MFLP; 6: MFHP; 7: VLFLP; 8: VLFHP.

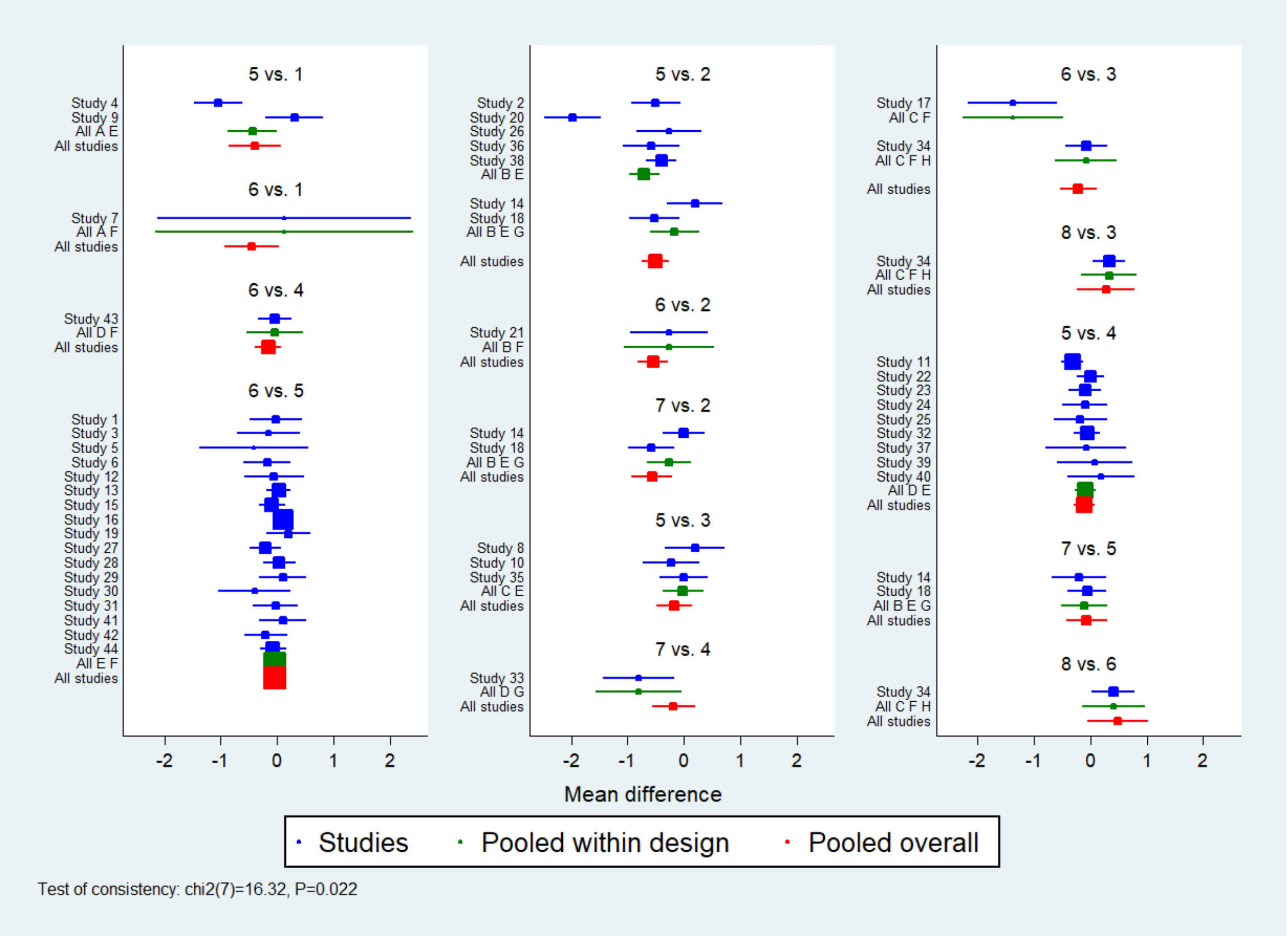

Figure S17 Inconsistency test chart of LDL cholesterol. P ＜ 0.05: No significant consistency between direct and circumstantial evidence. 1: VLCLP; 2: VLCHP; 3: MCHP; 4: MCLP; 5: MFLP; 6: MFHP; 7: VLFLP; 8: VLFHP.

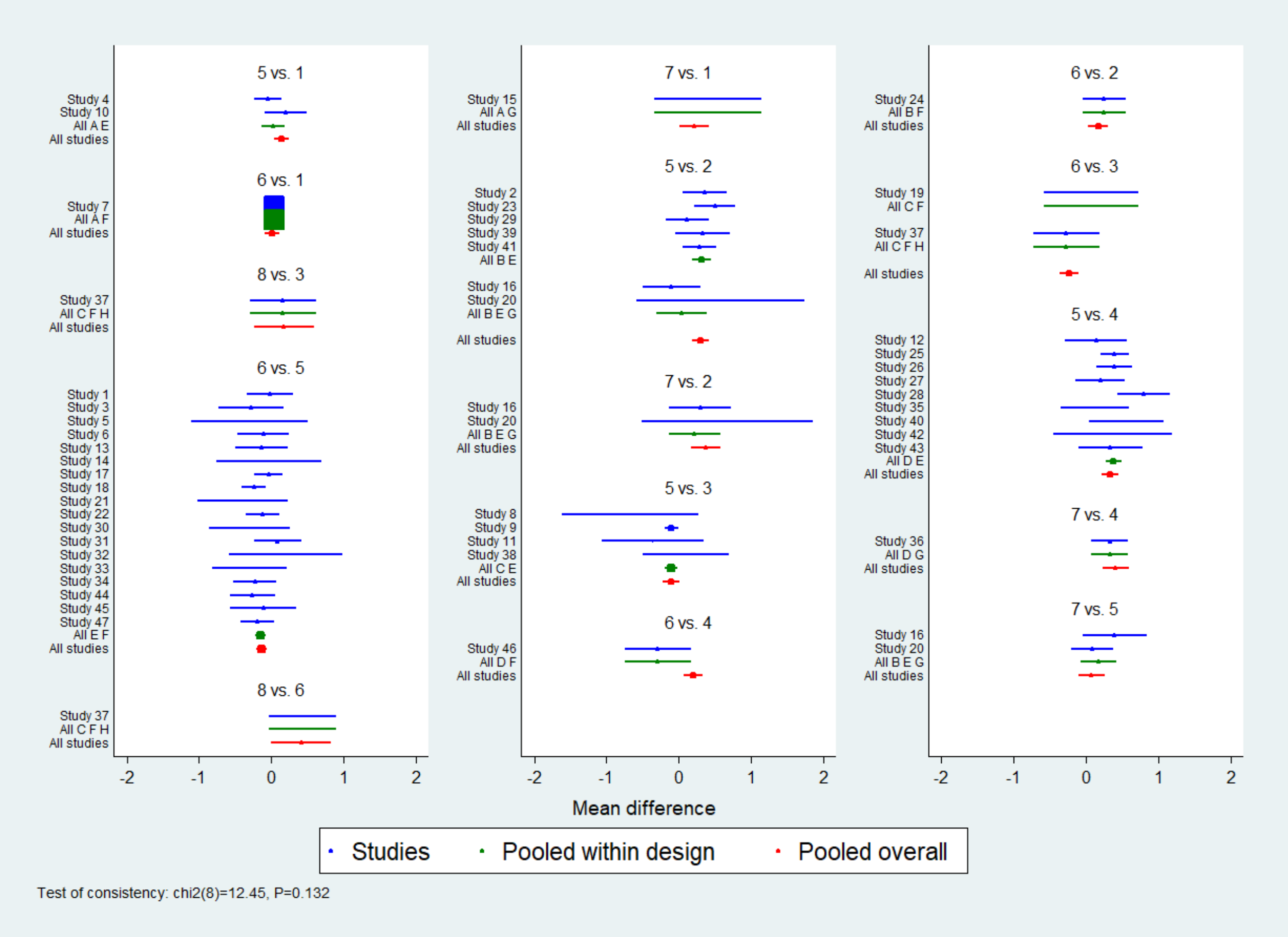

Figure S18 Inconsistency test chart of triglyceride. P > 0.05: Significant consistency between direct and circumstantial evidence. 1: VLCLP; 2: VLCHP; 3: MCHP; 4: MCLP; 5: MFLP; 6: MFHP; 7: VLFLP; 8: VLFHP.

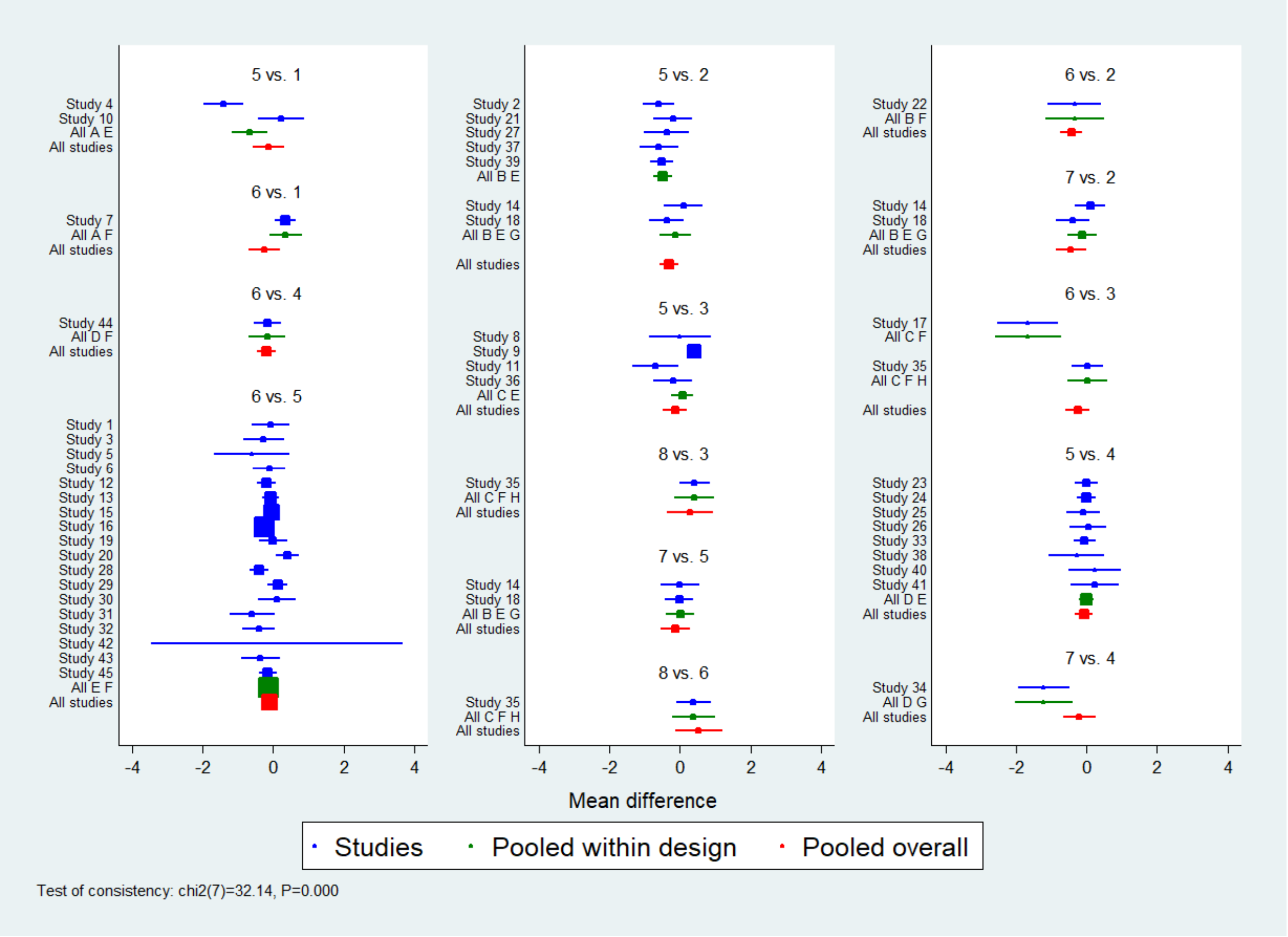

Figure S19 Inconsistency test chart of total cholesterol. $P < 0.05$: No significant consistency between direct and circumstantial evidence. 1: VLCLP; 2: VLCHP; 3: MCHP; 4: MCLP; 5: MFLP; 6: MFHP; 7: VLFLP; 8: VLFHP.

Figure S20-S27: Local inconsistency test chart for each outcome.

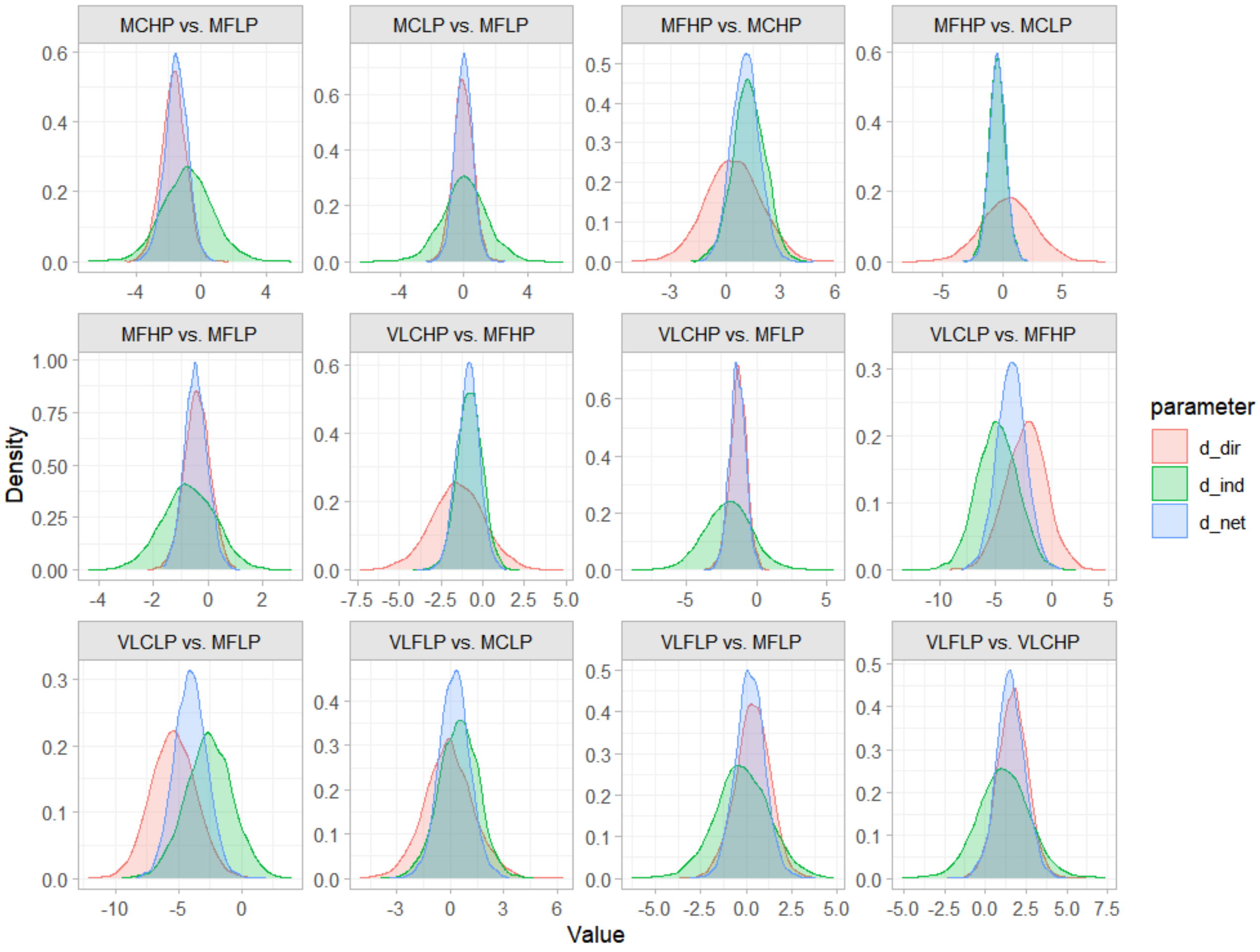

Figure S20 Local inconsistency test chart of weight change. P > 0.05: Significant consistency between direct and circumstantial evidence.

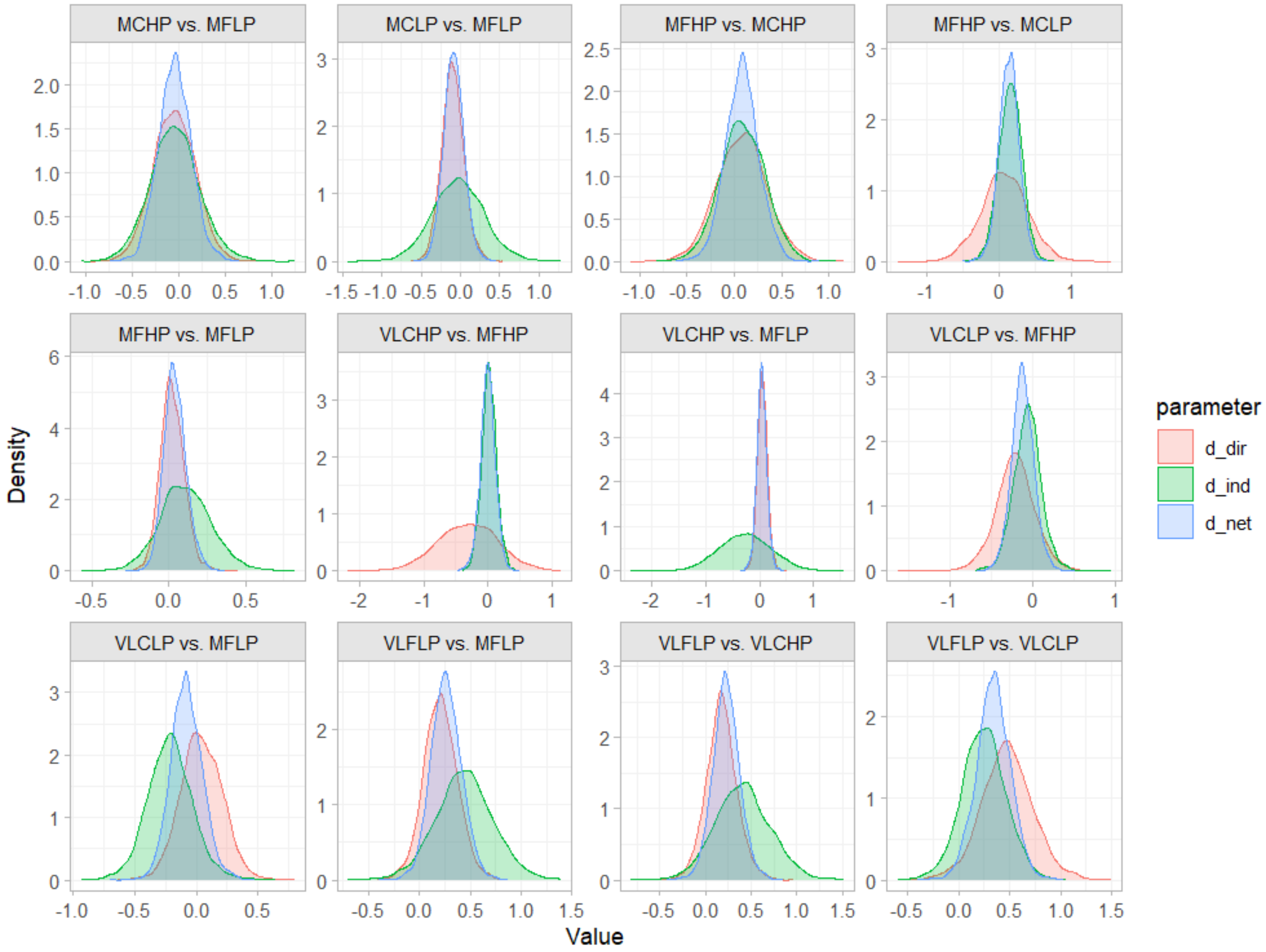

Figure S21 Local inconsistency test chart of blood glucose. P > 0.05: Significant consistency between direct and circumstantial evidence.

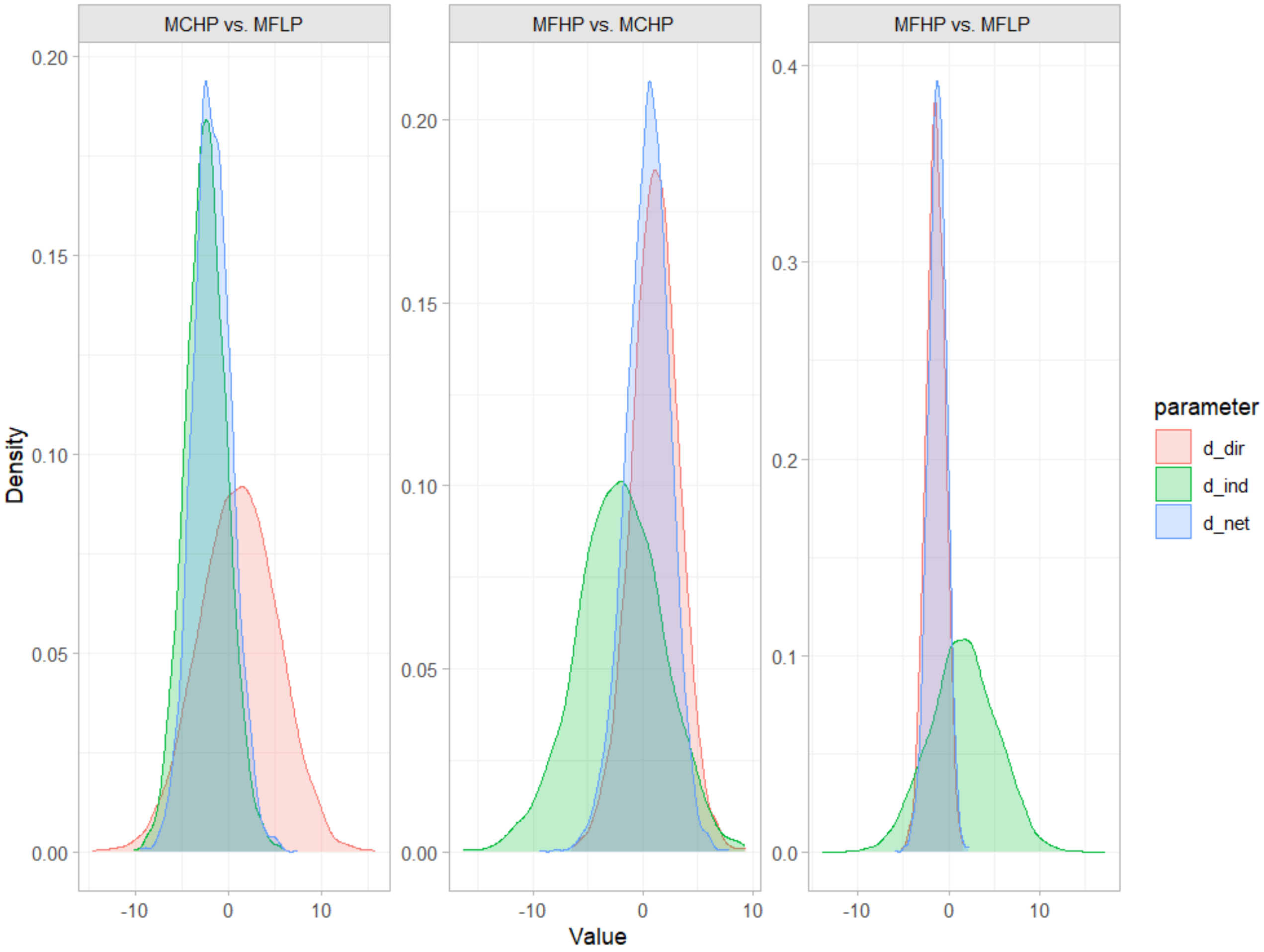

Figure S22 Local inconsistency test chart of systolic blood pressure. P > 0.05: Significant consistency between direct and circumstantial evidence.

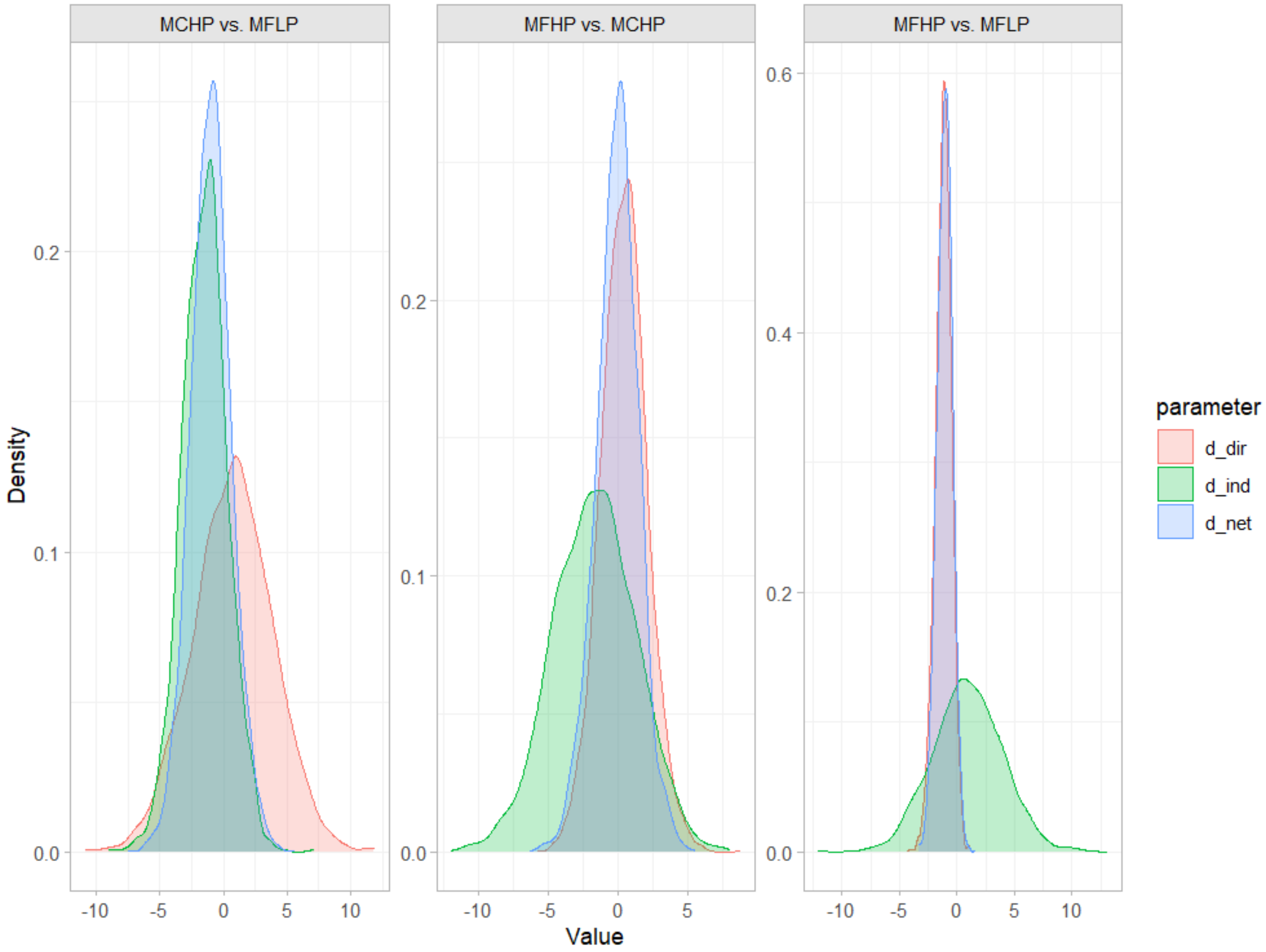

Figure S23 Local inconsistency test chart of diastolic blood pressure. P > 0.05: Significant consistency between direct and circumstantial evidence.

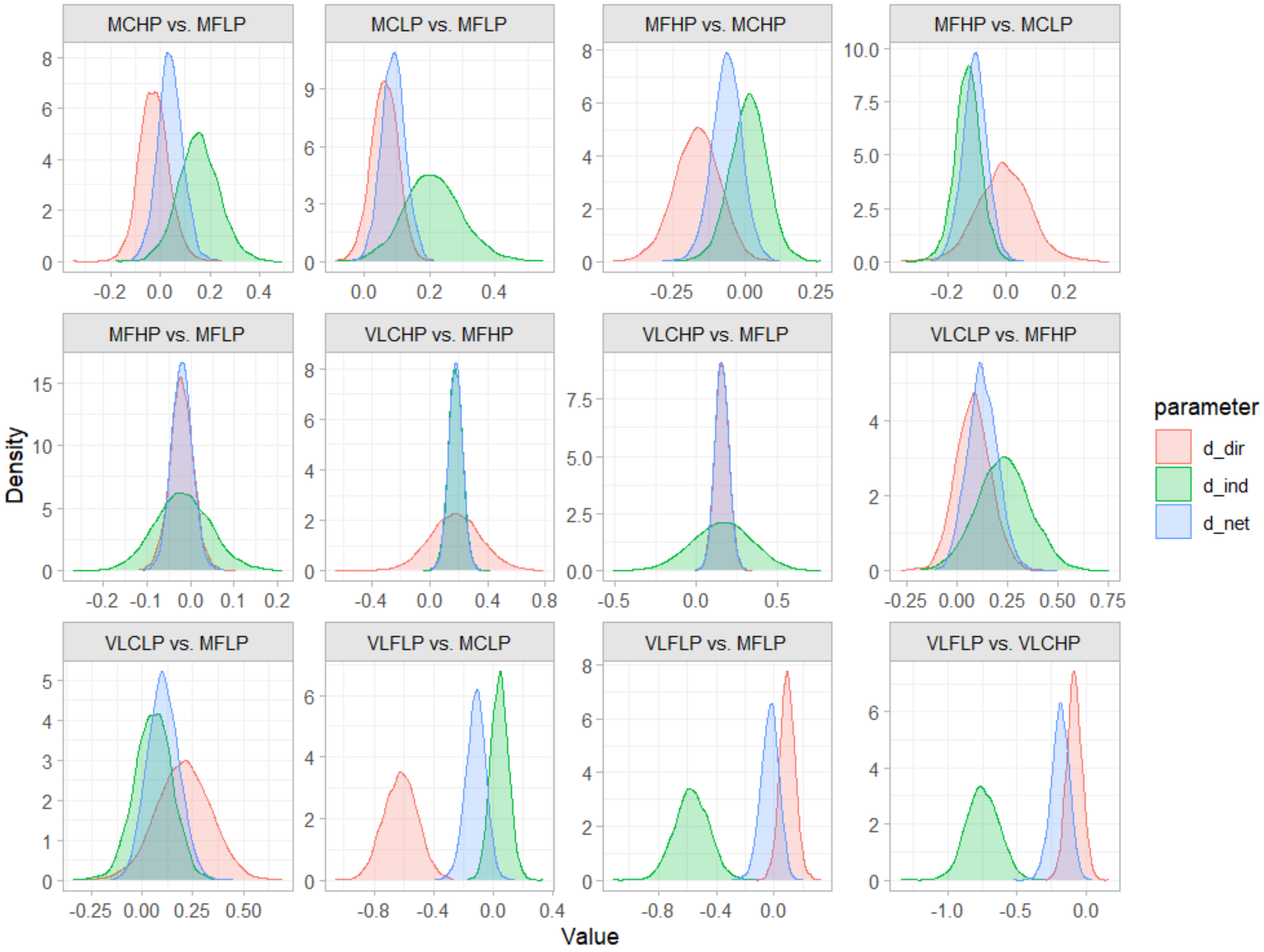

Figure S24 Local inconsistency test chart of HDL cholesterol. P > 0.05: Significant consistency between direct and circumstantial evidence.

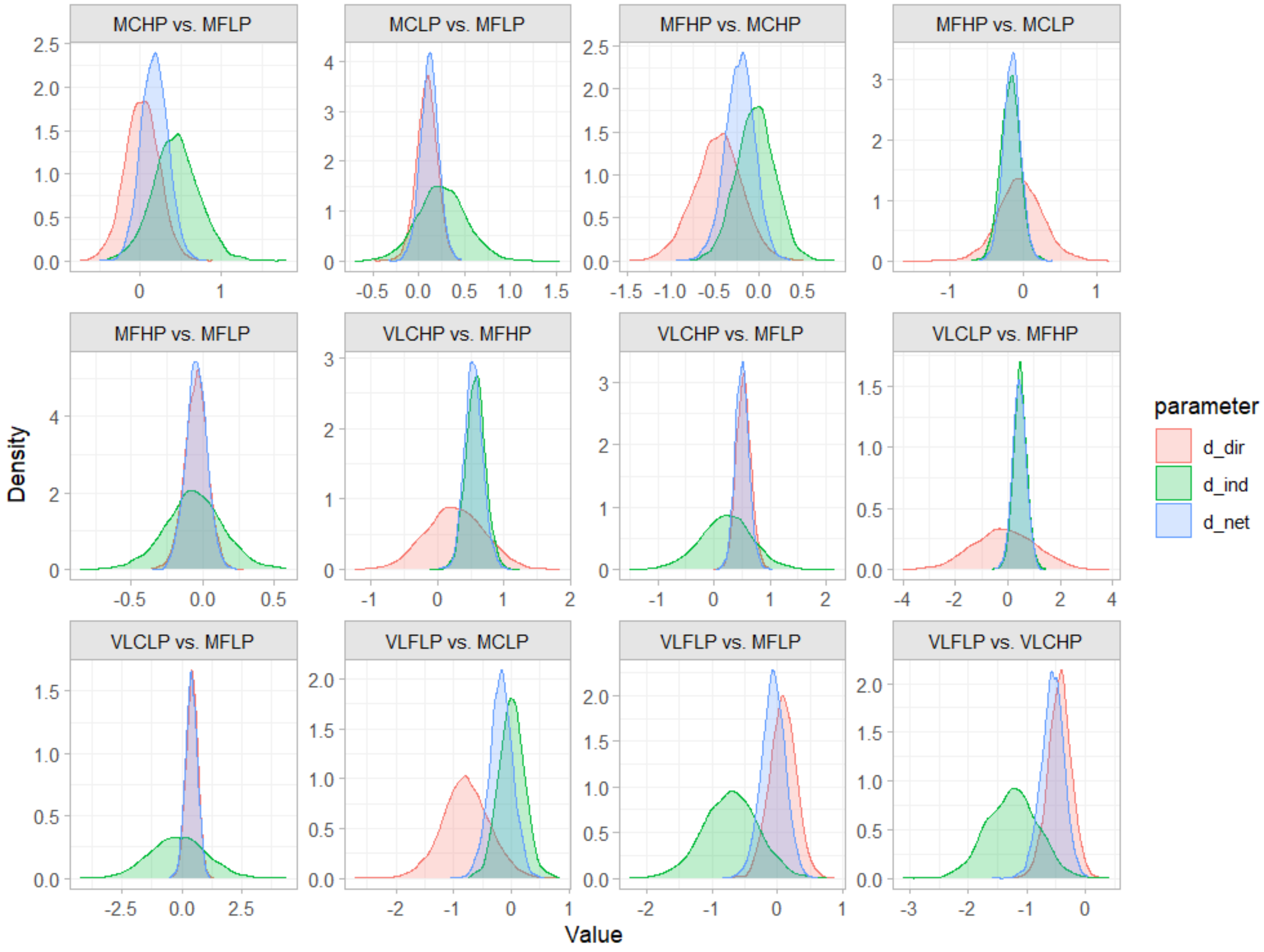

Figure S25 Local inconsistency test chart of LDL cholesterol. P > 0.05: Significant consistency between direct and circumstantial evidence.

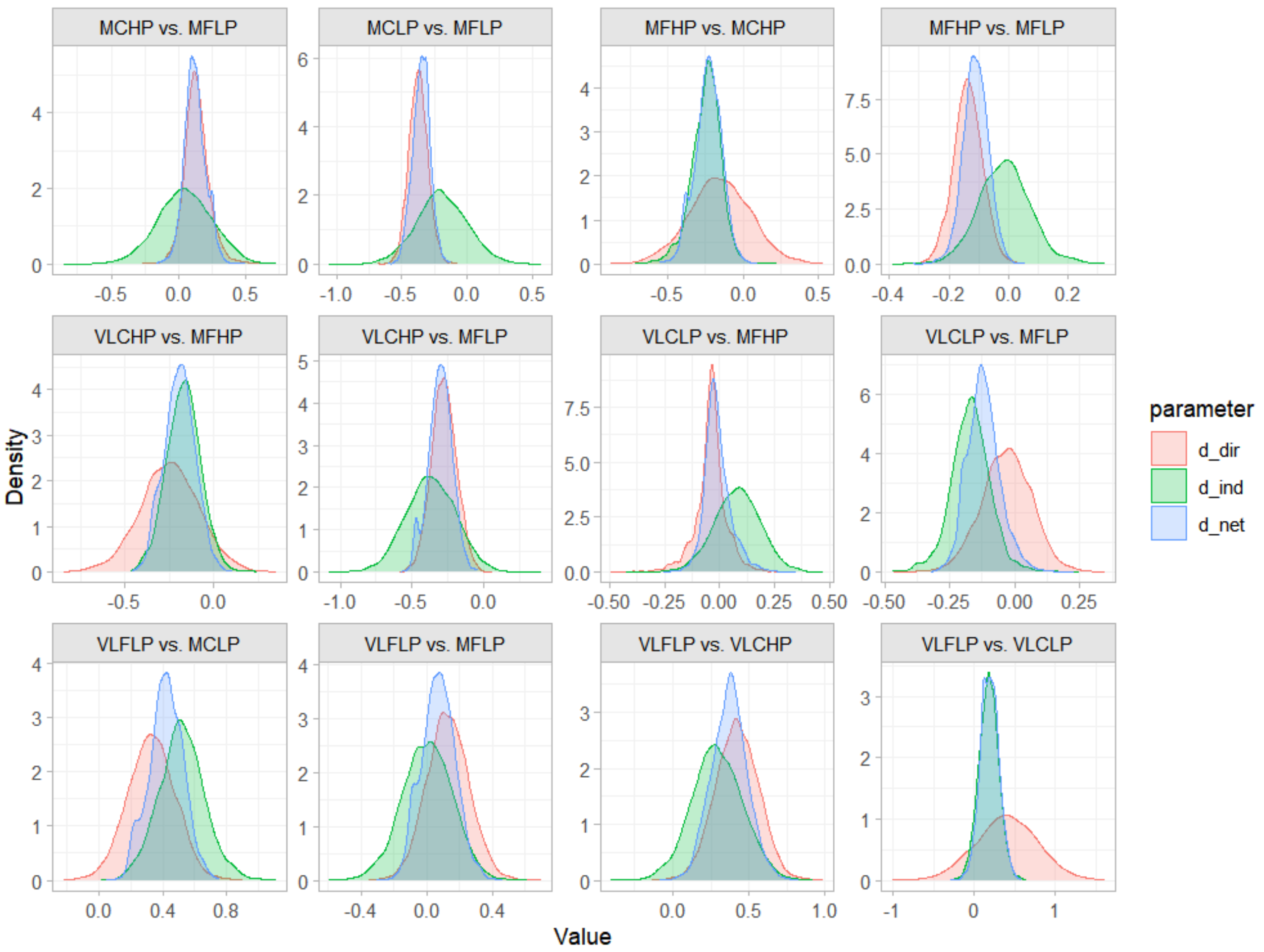

Figure S26 Local inconsistency test chart of triglyceride. P > 0.05: Significant consistency between direct and circumstantial evidence.

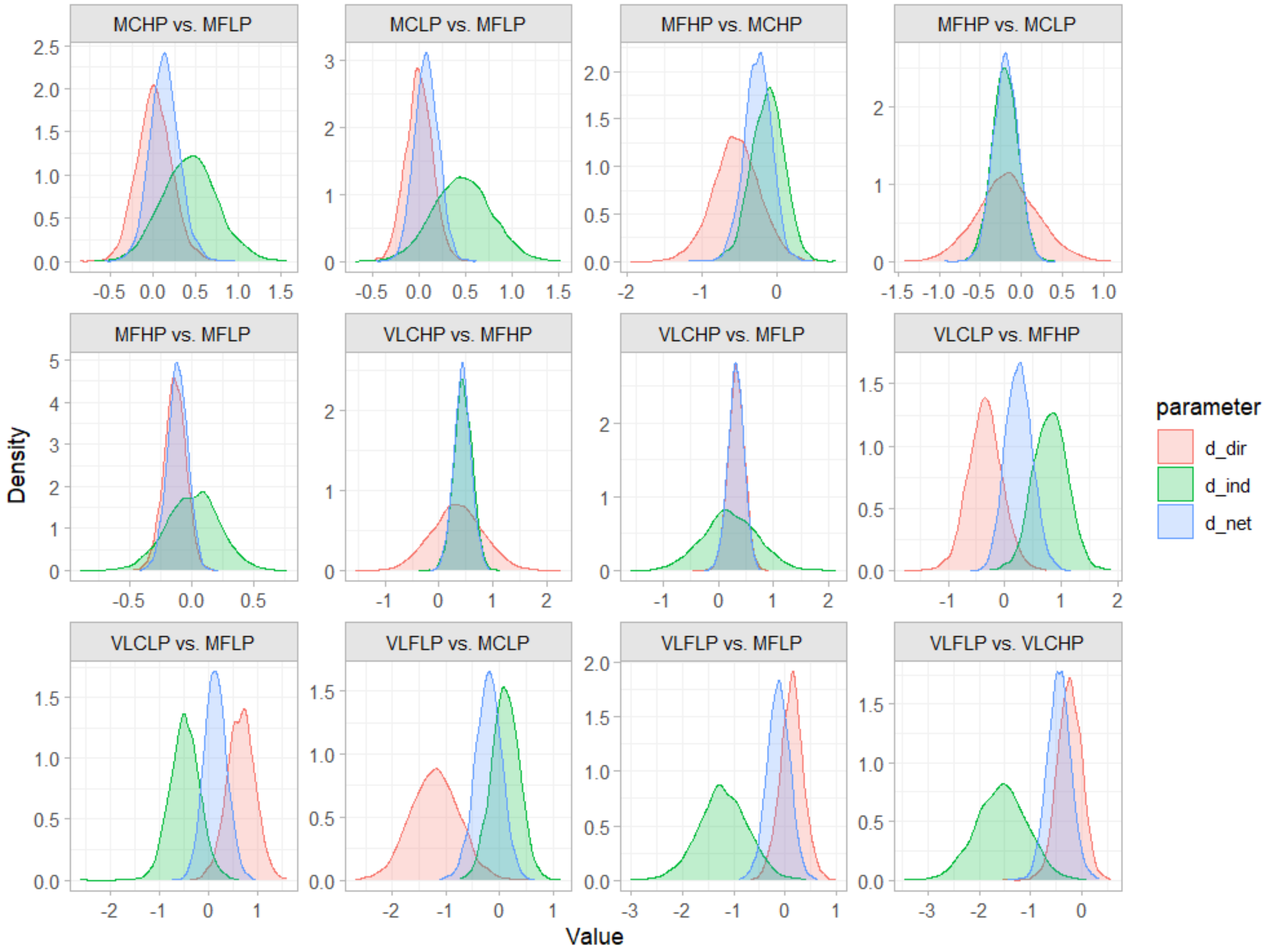

Figure S27 Local inconsistency test chart of total cholesterol. P > 0.05: Significant consistency between direct and circumstantial evidence.

Figure S28-S35: The funnel plots and egger's test values for each outcome.

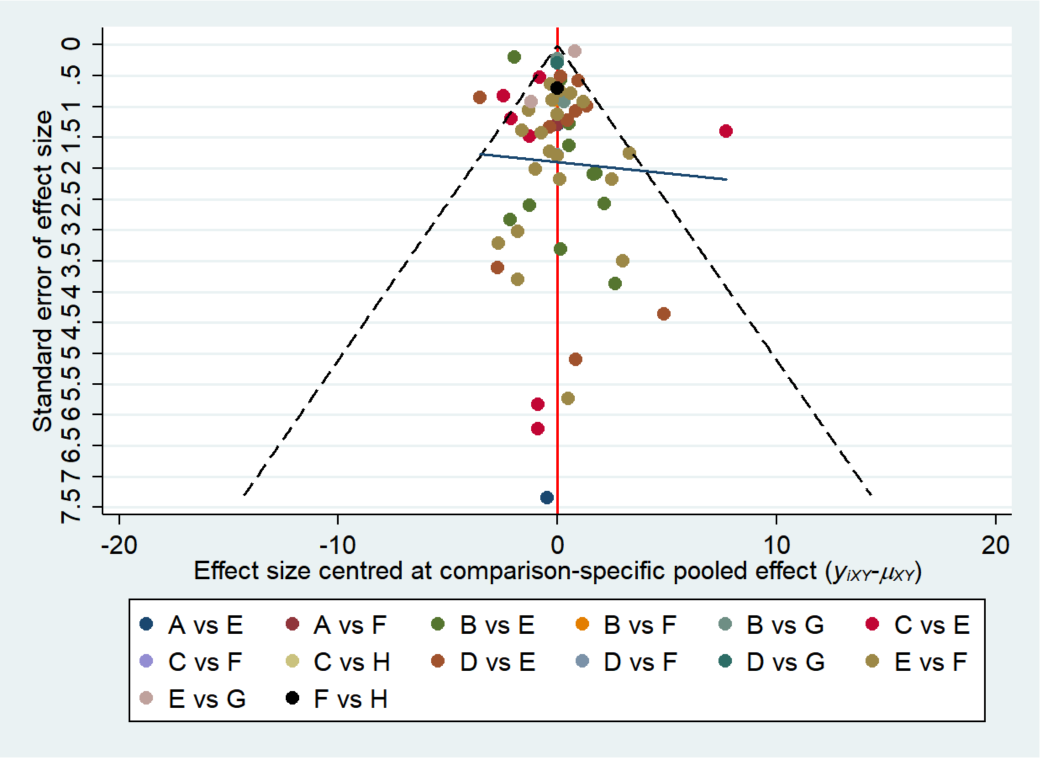

Figure S28 The funnel plot of weight change showing no significant publication bias (Egger's test: $p = 0.164$, $p > 0.05$). A: VLCLP; B: VLCHP; C: MCHP; D: MCLP; E: MFLP; F: MFHP; G: VLFLP; H: VLFHP.

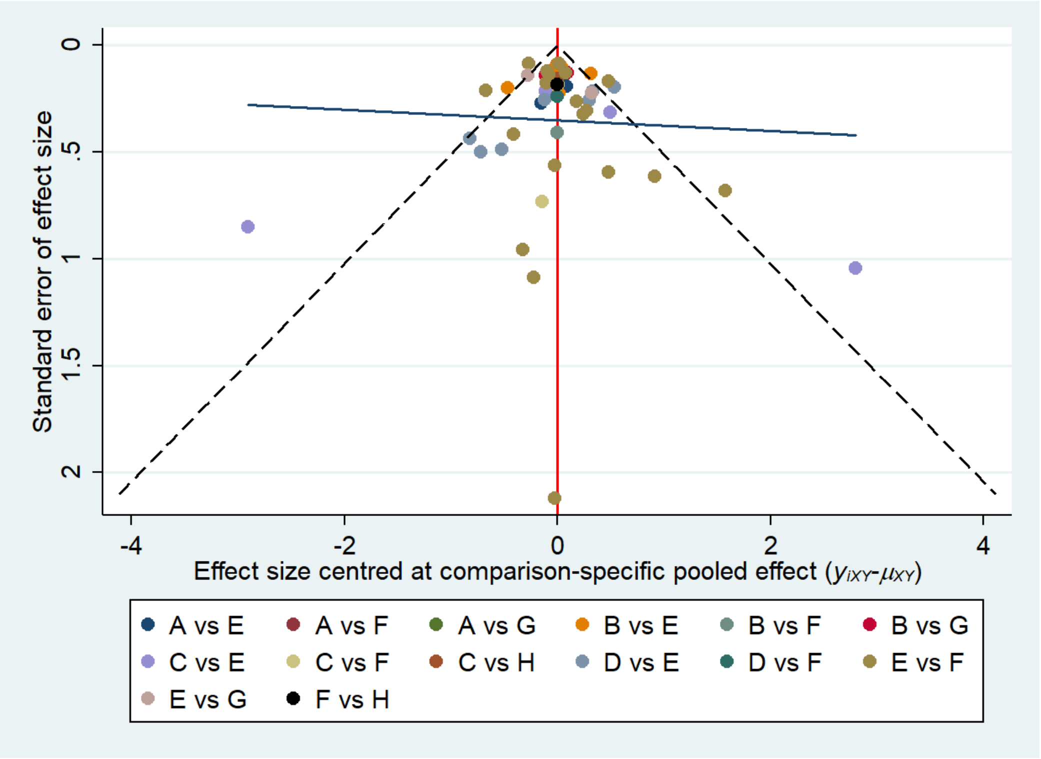

Figure S29 The funnel plot of blood glucose showing no significant publication bias (Egger's test: $p = 0.6$, $p > 0.05$). A: VLCLP; B: VLCHP; C: MCHP; D: MCLP; E: MFLP; F: MFHP; G: VLFLP; H: VLFHP.

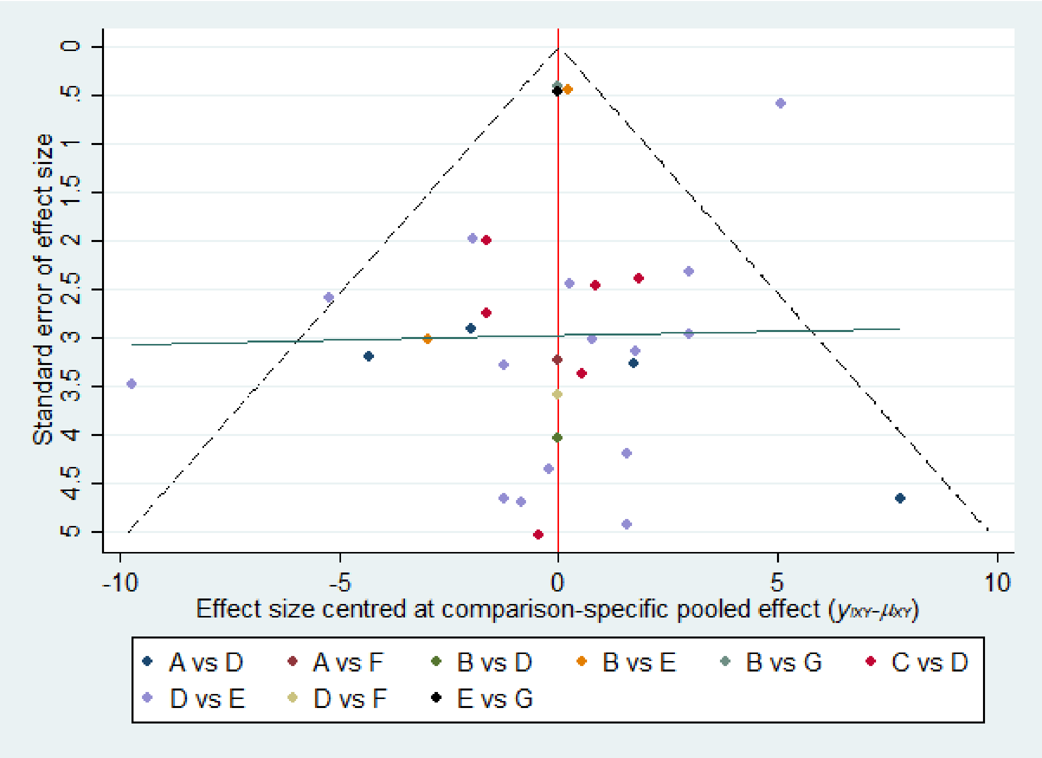

Figure S30 The funnel plot of systolic blood pressure showing no significant publication bias (Egger's test: $p = 0.183$, $p > 0.05$). A: VLCHP; B: MCHP; C: MCLP; D: MFLP; E: MFHP; F: VLFLP; G: VLFHP.

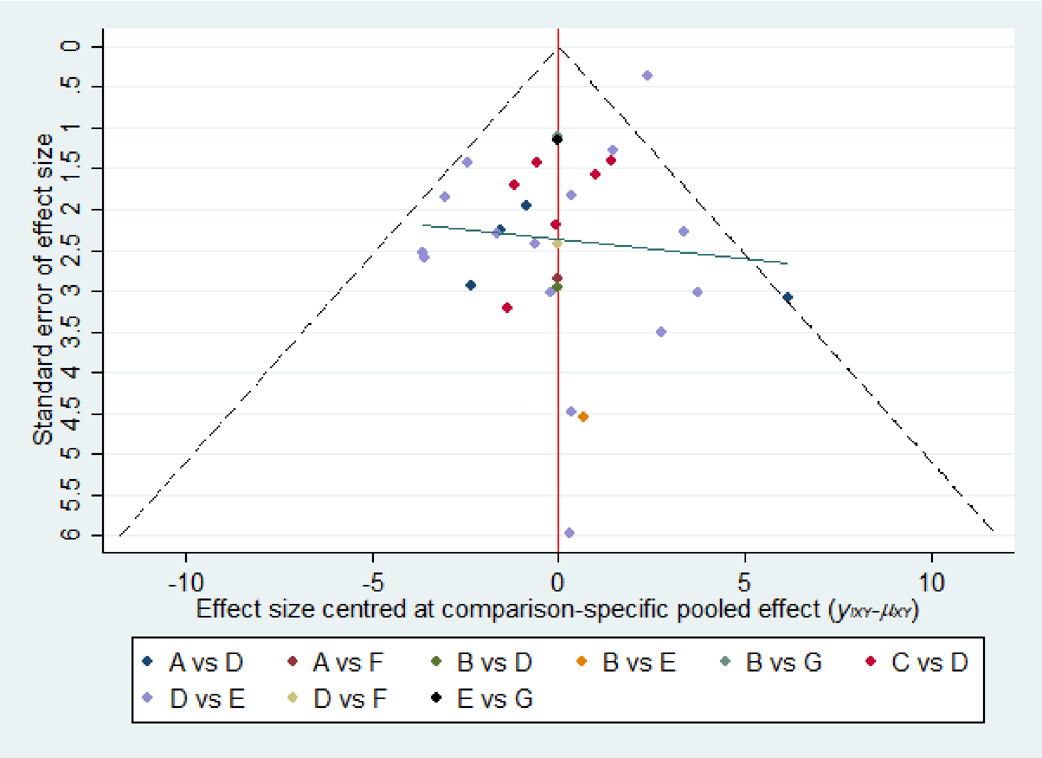

Figure S31 The funnel plot of diastolic blood pressure showing significant publication bias (Egger's test: $p = 0.012$, $p < 0.05$). A: VLCHP; B: MCHP; C: MCLP; D: MFLP; E: MFHP; F: VLFLP; G: VLFHP.

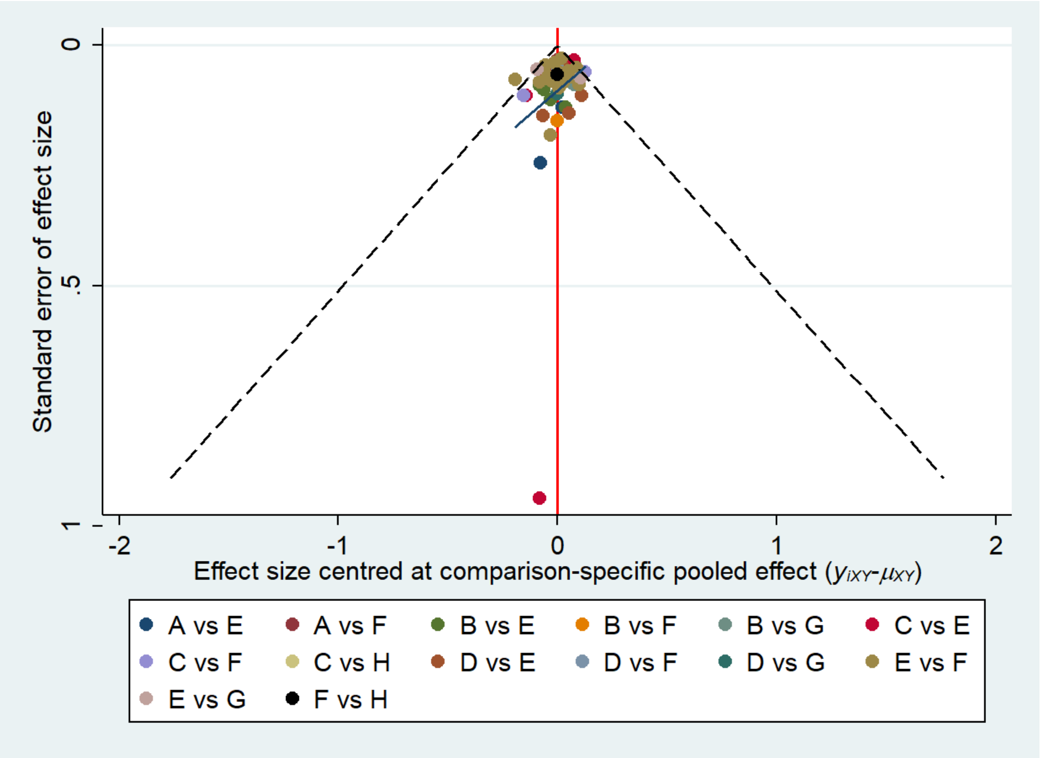

Figure S32 The funnel plot of HDL cholesterol showing significant publication bias (Egger's test: $p = 0.016$, $p < 0.05$). A: VLCLP; B: VLCHP; C: MCHP; D: MCLP; E: MFLP; F: MFHP; G: VLFLP; H: VLFHP.

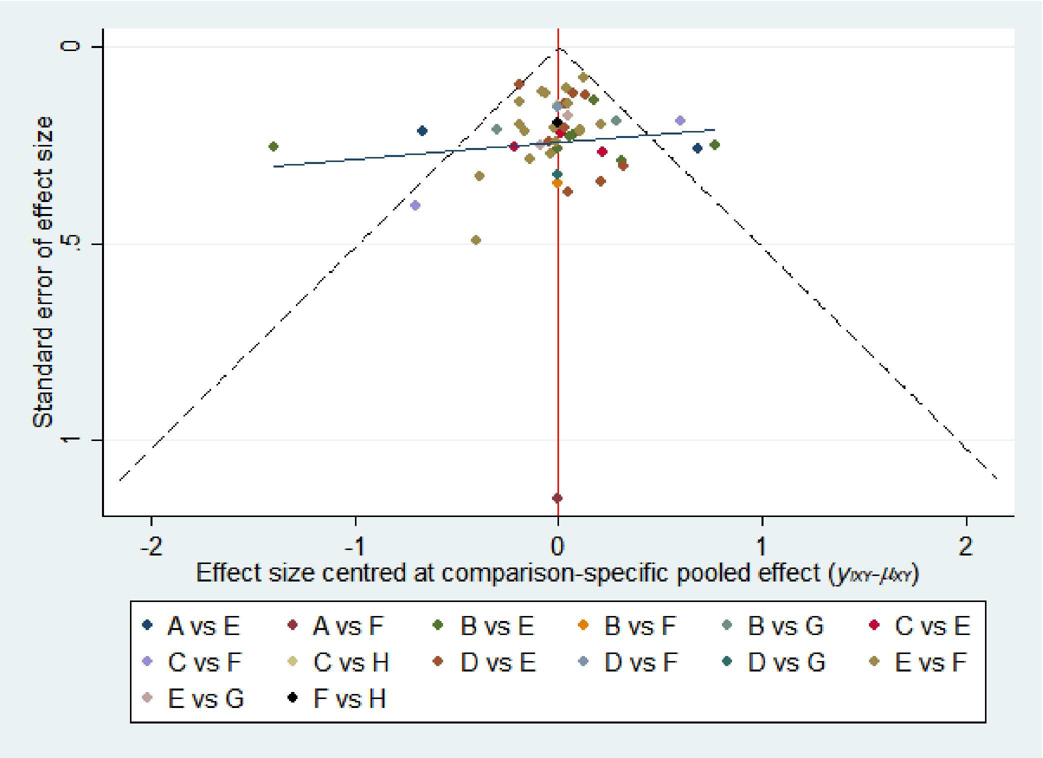

Figure S33 The funnel plot of LDL cholesterol showing no significant publication bias (Egger's test: $p = 0.059$, $p > 0.05$). A: VLCLP; B: VLCHP; C: MCHP; D: MCLP; E: MFLP; F: MFHP; G: VLFLP; H: VLFHP.

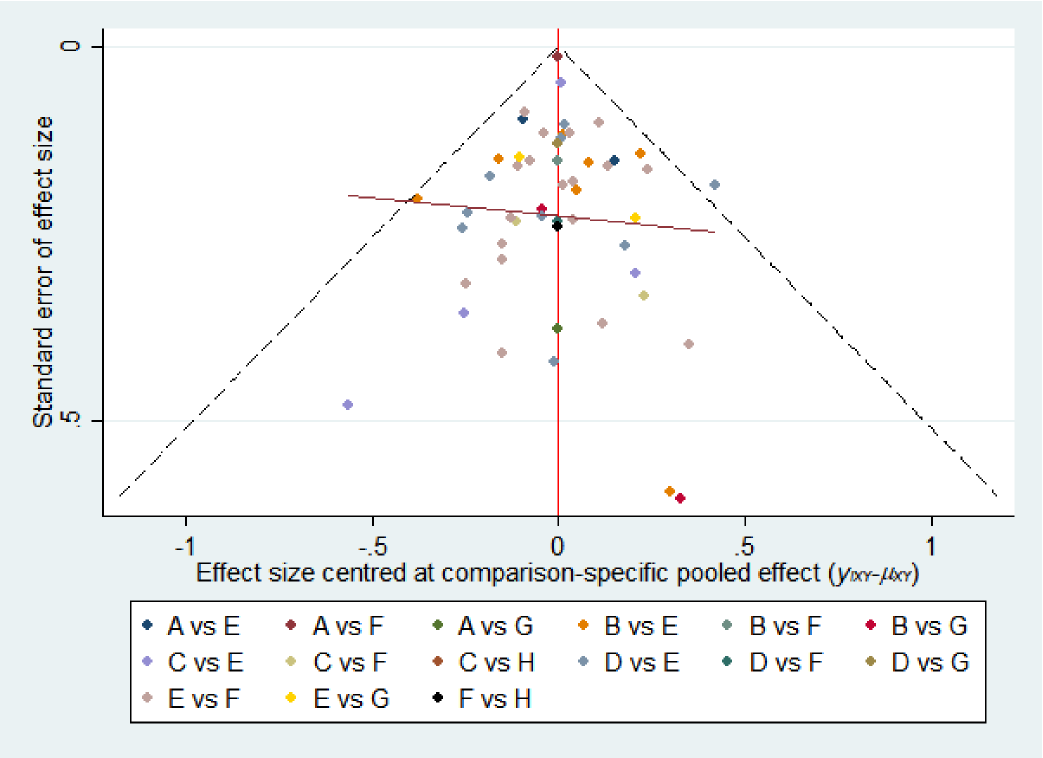

Figure S34 The funnel plot of triglyceride showing no significant publication bias (Egger's test: p = 0.309, p > 0.05). A: VLCLP; B: VLCHP; C: MCHP; D: MCLP; E: MFLP; F: MFHP; G: VLFLP; H: VLFHP.

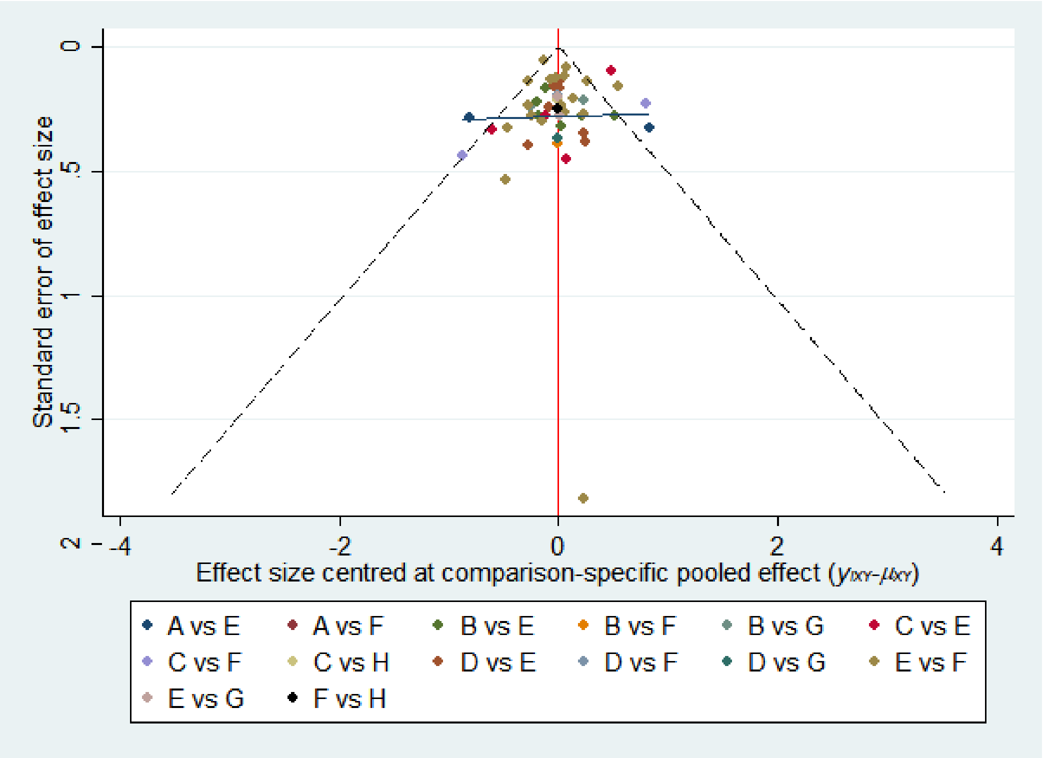

Figure S35 The funnel plot of total cholesterol showing no significant publication bias (Egger's test: p = 0.195, p > 0.05). A: VLCLP; B: VLCHP; C: MCHP; D: MCLP; E: MFLP; F: MFHP; G: VLFLP; H: VLFHP.

Table S5: Transitivity (Indirectness) Assessment

	Baseline variable (Mean ± SD)	
Intervention	Age(year)	BMI
Moderate fat-low protein diet (MFLP)	52.3±10.0	34.3±4.7
Moderate fat-high protein diet (MFHP)	52.4±11.1	34.6±5.5
Moderate carbohydrate-low protein diet (MCLP)	53.9±11.2	34.2±5.5
Moderate carbohydrate-high protein diet (MCHP)	51.4±13.2	34.1±4.8
Very low fat-low protein diet (VLFLP)	46.4±14.4	33.8±4.9
Very low fat-high protein diet (VLFHP)	41.3±12.4	35.4±3.9
Very low carbohydrate-low protein diet (VLCLP)	46.4±14.4	33.9±4.9
Very low carbohydrate-high protein diet (VLCHP)	49.1±5.7	33.6±3.1

Table S6: CINeMA for the outcome “weight change” in dietary groups NMA.

Comparison	Number of studies	Nature of evidence	Confidence level	Downgrading reason
MCHP:MFHP	1	Mixed	Low	["Imprecision"]
MCHP:MFLP	7	Mixed	Low	["Heterogeneity"]
MCHP:VLFHP	1	Mixed	Low	["Imprecision"]
MCLP:MFHP	1	Mixed	Low	["Imprecision"]
MCLP:MFLP	10	Mixed	Low	["Imprecision"]
MCLP:VLFLP	1	Mixed	Very low	["Within-study bias", "Imprecision"]
MFHP:MFLP	20	Mixed	Low	["Imprecision"]
MFHP:VLCHP	1	Mixed	Low	["Imprecision"]
MFHP:VLCLP	1	Mixed	High	[]
MFHP:VLFHP	1	Mixed	Low	["Imprecision"]
MFLP:VLCHP	12	Mixed	Low	["Heterogeneity"]
MFLP:VLCLP	2	Mixed	High	[]
MFLP:VLFLP	2	Mixed	Very low	["Within-study bias", "Imprecision"]
VLCHP:VLFLP	2	Mixed	Low	["Imprecision"]
MCHP:MCLP	—	Indirect	Low	["Imprecision"]
MCHP:VLCHP	—	Indirect	Low	["Imprecision"]
MCHP:VLCLP	—	Indirect	Low	["Imprecision"]
MCHP:VLFLP	—	Indirect	Low	["Imprecision"]
MCLP:VLCHP	—	Indirect	Low	["Imprecision"]
MCLP:VLCLP	—	Indirect	High	[]
MCLP:VLFHP	—	Indirect	Low	["Imprecision"]
MFHP:VLFLP	—	Indirect	Low	["Imprecision"]
MFLP:VLFHP	—	Indirect	Low	["Imprecision"]
VLCHP:VLCLP	—	Indirect	Low	["Heterogeneity"]
VLCHP:VLFHP	—	Indirect	Low	["Imprecision"]
VLCLP:VLFHP	—	Indirect	High	[]
VLCLP:VLFLP	—	Indirect	High	[]
VLFHP:VLFLP	—	Indirect	Low	["Imprecision"]

Table S7: CINeMA for the outcome "blood glucose" in dietary groups NMA.

Comparison	Number of studies	Nature of evidence	Confidence level	Downgrading reason
MCHP:MFHP	2	Mixed	Low	["Imprecision"]
MCHP:MFLP	4	Mixed	Very low	["Within-study bias", "Imprecision"]
MCHP:VLFHP	1	Mixed	Low	["Imprecision"]
MCLP:MFLP	1	Mixed	Low	["Imprecision"]
MFHP:MFLP	7	Mixed	Low	["Imprecision"]
MFHP:VLCHP	19	Mixed	Low	["Imprecision"]
MFHP:VLCLP	1	Mixed	Low	["Imprecision"]
MFHP:VLFHP	1	Mixed	Low	["Imprecision"]
MFLP:VLCHP	1	Mixed	Low	["Imprecision"]
MFLP:VLCLP	7	Mixed	Low	["Imprecision"]
MFLP:VLFLP	3	Mixed	Very low	["Within-study bias", "Imprecision"]
VLCHP:VLFLP	2	Mixed	Low	["Imprecision"]
VLCLP:VLFLP	2	Mixed	Low	["Imprecision"]
MCHP:MCLP	—	Indirect	Very low	["Within-study bias", "Heterogeneity"]
MCHP:VLCHP	—	Indirect	Low	["Imprecision"]
MCHP:VLCLP	—	Indirect	Low	["Imprecision"]
MCHP:VLFLP	—	Indirect	Very low	["Within-study bias", "Imprecision"]
MCLP:MFHP	—	Indirect	Low	["Imprecision"]
MCLP:VLCHP	—	Indirect	Low	["Imprecision"]
MCLP:VLCLP	—	Indirect	Low	["Imprecision"]
MCLP:VLFHP	—	Indirect	Low	["Imprecision"]
MCLP:VLFLP	—	Indirect	Low	["Imprecision"]
MFHP:VLFLP	—	Indirect	Low	["Imprecision"]
MFLP:VLFHP	—	Indirect	Low	["Imprecision"]
VLCHP:VLCLP	—	Indirect	Low	["Imprecision"]
VLCHP:VLFHP	—	Indirect	Low	["Imprecision"]
VLCLP:VLFHP	—	Indirect	Low	["Imprecision"]
VLFHP:VLFLP	—	Indirect	Low	["Imprecision"]

Table S8: CINeMA for the outcome "systolic blood pressure" in dietary groups NMA.

Comparison	Number of studies	Nature of evidence	Confidence level	Downgrading reason
MCHP:MFHP	2	Mixed	Low	["Imprecision"]
MCHP:VLFHP	1	Mixed	Low	["Imprecision"]
MCLP:MFLP	1	Mixed	Low	["Imprecision"]
MFHP:MFLP	6	Mixed	Low	["Imprecision"]
MFHP:VLFHP	15	Mixed	Low	["Imprecision"]
MFLP:VLCHP	1	Mixed	Low	["Imprecision"]
MFLP:VLFLP	4	Mixed	Low	["Imprecision"]
VLCHP:VLFLP	1	Mixed	Very low	["Within-study bias", "Imprecision"]
MCHP:MCLP	—	Indirect	Very low	["Within-study bias", "Imprecision"]
MCHP:MFLP	—	Indirect	Low	["Imprecision"]
MCHP:VLCHP	—	Indirect	Low	["Imprecision"]
MCHP:VLFLP	—	Indirect	Low	["Imprecision"]
MCLP:MFHP	—	Indirect	Low	["Imprecision"]
MCLP:VLCHP	—	Indirect	Low	["Imprecision"]
MCLP:VLFHP	—	Indirect	Low	["Imprecision"]
MCLP:VLFLP	—	Indirect	Very low	["Within-study bias", "Imprecision"]
MFHP:VLCHP	—	Indirect	Low	["Imprecision"]
MFHP:VLFLP	—	Indirect	Very low	["Within-study bias", "Imprecision"]
MFLP:VLFHP	—	Indirect	Low	["Imprecision"]
VLCHP:VLFHP	—	Indirect	Low	["Imprecision"]
VLFHP:VLFLP	—	Indirect	Low	["Imprecision"]

Table S9: CINeMA for the outcome “diastolic blood pressure” in dietary groups NMA.

Comparison	Number of studies	Nature of evidence	Confidence level	Downgrading reason
MCHP:MFHP	2	Mixed	Very low	["Reporting bias", "Imprecision"]
MCHP:MFLP	1	Mixed	Very low	["Reporting bias", "Imprecision"]
MCHP:VLFHP	1	Mixed	Very low	["Reporting bias", "Imprecision"]
MCLP:MFLP	6	Mixed	Very low	["Reporting bias", "Imprecision"]
MFHP:MFLP	15	Mixed	Very low	["Reporting bias", "Imprecision"]
MFHP:VLFHP	1	Mixed	Very low	["Reporting bias", "Imprecision"]
MFLP:VLCHP	4	Mixed	Very low	["Reporting bias", "Imprecision"]
MFLP:VLFLP	1	Mixed	Very low	["Within-study bias", "Reporting bias", "Imprecision"]
VLCHP:VLFLP	1	Mixed	Very low	["Within-study bias", "Reporting bias", "Imprecision"]
MCHP:MCLP	—	Indirect	Very low	["Reporting bias", "Imprecision"]
MCHP:VLCHP	—	Indirect	Very low	["Reporting bias", "Imprecision"]
MCHP:VLFLP	—	Indirect	Very low	["Reporting bias", "Imprecision"]
MCLP:MFHP	—	Indirect	Very low	["Reporting bias", "Imprecision"]
MCLP:VLCHP	—	Indirect	Very low	["Reporting bias", "Imprecision"]
MCLP:VLFHP	—	Indirect	Very low	["Reporting bias", "Imprecision"]
MCLP:VLFLP	—	Indirect	Very low	["Within-study bias", "Reporting bias", "Imprecision"]
MFHP:VLCHP	—	Indirect	Very low	["Reporting bias", "Imprecision"]
MFHP:VLFLP	—	Indirect	Very low	["Within-study bias", "Reporting bias", "Imprecision"]
MFLP:VLFHP	—	Indirect	Very low	["Reporting bias", "Imprecision"]
VLCHP:VLFHP	—	Indirect	Very low	["Reporting bias", "Imprecision"]
VLFHP:VLFLP	—	Indirect	Very low	["Reporting bias", "Imprecision"]

Table S10: CINeMA for the outcome "HDL cholesterol" in dietary groups NMA.

Comparison	Number of studies	Nature of evidence	Confidence level	Downgrading reason
MCHP:MFHP	2	Mixed	Very low	["Within-study bias", "Reporting bias", "Imprecision", "Incoherence"]
MCHP:MFLP	4	Mixed	Very low	["Within-study bias", "Reporting bias", "Imprecision", "Incoherence"]
MCHP:VLFHP	1	Mixed	Very low	["Reporting bias", "Imprecision"]
MCLP:MFHP	1	Mixed	Very low	["Reporting bias", "Heterogeneity", "Incoherence"]
MCLP:MFLP	8	Mixed	Very low	["Reporting bias", "Heterogeneity"]
MCLP:VLFLP	1	Mixed	Very low	["Reporting bias", "Imprecision", "Incoherence"]
MFHP:MFLP	17	Mixed	Very low	["Reporting bias", "Imprecision"]
MFHP:VLCHP	1	Mixed	Low	["Reporting bias"]
MFHP:VLCLP	1	Mixed	Very low	["Reporting bias", "Heterogeneity"]
MFHP:VLFHP	1	Mixed	Very low	["Reporting bias", "Imprecision"]
MFLP:VLCHP	7	Mixed	Very low	["Reporting bias", "Heterogeneity", "Incoherence"]
MFLP:VLCLP	2	Mixed	Very low	["Reporting bias", "Heterogeneity"]
MFLP:VLFLP	2	Mixed	Very low	["Reporting bias", "Imprecision", "Incoherence"]
VLCHP:VLFLP	2	Mixed	Very low	["Reporting bias", "Heterogeneity"]
MCHP:MCLP	—	Indirect	Very low	["Within-study bias", "Reporting bias", "Heterogeneity", "Incoherence"]
MCHP:VLCHP	—	Indirect	Very low	["Within-study bias", "Reporting bias", "Heterogeneity", "Incoherence"]
MCHP:VLCLP	—	Indirect	Very low	["Reporting bias", "Heterogeneity", "Incoherence"]
MCHP:VLFLP	—	Indirect	Very low	["Within-study bias", "Reporting bias", "Imprecision", "Incoherence"]
MCLP:VLCHP	—	Indirect	Very low	["Reporting bias", "Heterogeneity", "Incoherence"]
MCLP:VLCLP	—	Indirect	Very low	["Reporting bias", "Imprecision", "Incoherence"]
MCLP:VLFHP	—	Indirect	Very low	["Reporting bias", "Imprecision", "Incoherence"]
MFHP:VLFLP	—	Indirect	Very low	["Reporting bias", "Imprecision", "Incoherence"]
MFLP:VLFHP	—	Indirect	Very low	["Reporting bias", "Imprecision", "Incoherence"]
VLCHP:VLCLP	—	Indirect	Very low	["Reporting bias", "Heterogeneity", "Incoherence"]
VLCHP:VLFHP	—	Indirect	Very low	["Reporting bias", "Heterogeneity", "Incoherence"]
VLCLP:VLFHP	—	Indirect	Very low	["Reporting bias", "Imprecision", "Incoherence"]
VLCLP:VLFLP	—	Indirect	Very low	["Reporting bias", "Imprecision", "Incoherence"]
VLFHP:VLFLP	—	Indirect	Very low	["Reporting bias", "Imprecision", "Incoherence"]

Table S11: CINeMA for the outcome "LDL cholesterol" in dietary groups NMA.

Comparison	Number of studies	Nature of evidence	Confidence level	Downgrading reason
MCHP:MFHP	2	Mixed	Low	["Imprecision"]
MCHP:MFLP	3	Mixed	Very low	["Within-study bias", "Imprecision"]
MCHP:VLFHP	1	Mixed	Low	["Imprecision"]
MCLP:MFHP	1	Mixed	Low	["Imprecision"]
MCLP:MFLP	9	Mixed	Low	["Imprecision"]
MCLP:VLFLP	1	Mixed	Very low	["Imprecision", "Incoherence"]
MFHP:MFLP	17	Mixed	Low	["Imprecision"]
MFHP:VLCHP	1	Mixed	High	[]
MFHP:VLCLP	1	Mixed	Low	["Imprecision"]
MFHP:VLFHP	1	Mixed	Low	["Imprecision"]
MFLP:VLCHP	7	Mixed	High	["Incoherence"]
MFLP:VLCLP	2	Mixed	Very low	["Within-study bias", "Imprecision"]
MFLP:VLFLP	2	Mixed	Low	["Imprecision"]
VLCHP:VLFLP	2	Mixed	Low	["Heterogeneity", "Incoherence"]
MCHP:MCLP	—	Indirect	Very low	["Imprecision", "Incoherence"]
MCHP:VLCHP	—	Indirect	Very low	["Within-study bias", "Imprecision", "Incoherence"]
MCHP:VLCLP	—	Indirect	Very low	["Within-study bias", "Imprecision", "Incoherence"]
MCHP:VLFLP	—	Indirect	Very low	["Within-study bias", "Imprecision", "Incoherence"]
MCLP:VLCHP	—	Indirect	Low	["Heterogeneity", "Incoherence"]
MCLP:VLCLP	—	Indirect	Very low	["Imprecision", "Incoherence"]
MCLP:VLFHP	—	Indirect	Very low	["Imprecision", "Incoherence"]
MFHP:VLFLP	—	Indirect	Very low	["Imprecision", "Incoherence"]
MFLP:VLFHP	—	Indirect	Very low	["Imprecision", "Incoherence"]
VLCHP:VLCLP	—	Indirect	Very low	["Imprecision", "Incoherence"]
VLCHP:VLFHP	—	Indirect	Very low	["Imprecision", "Incoherence"]
VLCLP:VLFHP	—	Indirect	Very low	["Imprecision", "Incoherence"]
VLCLP:VLFLP	—	Indirect	Very low	["Imprecision", "Incoherence"]
VLFHP:VLFLP	—	Indirect	Very low	["Imprecision", "Incoherence"]

Table S12: CINeMA for the outcome "triglyceride" in dietary groups NMA.

Comparison	Number of studies	Nature of evidence	Confidence level	Downgrading reason
MCHP:MFHP	2	Mixed	Moderate	["Within-study bias"]
MCHP:MFLP	4	Mixed	Moderate	["Within-study bias"]
MCHP:VLFHP	1	Mixed	Low	["Imprecision"]
MCLP:MFHP	1	Mixed	Low	["Incoherence"]
MCLP:MFLP	9	Mixed	Low	["Incoherence"]
MCLP:VLFLP	1	Mixed	Moderate	["Within-study bias"]
MFHP:MFLP	18	Mixed	High	[]
MFHP:VLCHP	1	Mixed	Moderate	["Within-study bias"]
MFHP:VLCLP	1	Mixed	Low	["Incoherence"]
MFHP:VLFHP	1	Mixed	Low	["Heterogeneity"]
MFLP:VLCHP	7	Mixed	High	[]
MFLP:VLCLP	2	Mixed	Low	["Incoherence"]
MFLP:VLFLP	2	Mixed	Low	["Imprecision"]
VLCHP:VLFLP	2	Mixed	Moderate	["Within-study bias"]
VLCLP:VLFLP	1	Mixed	High	[]
MCHP:MCLP	—	Indirect	Moderate	["Within-study bias"]
MCHP:VLCHP	—	Indirect	Moderate	["Within-study bias"]
MCHP:VLCLP	—	Indirect	High	[]
MCHP:VLFLP	—	Indirect	Very low	["Within-study bias", "Imprecision"]
MCLP:VLCHP	—	Indirect	Low	["Imprecision"]
MCLP:VLCLP	—	Indirect	High	[]
MCLP:VLFHP	—	Indirect	High	[]
MFHP:VLFLP	—	Indirect	High	[]
MFLP:VLFHP	—	Indirect	Low	["Imprecision"]
VLCHP:VLCLP	—	Indirect	High	[]
VLCHP:VLFHP	—	Indirect	High	[]
VLCLP:VLFHP	—	Indirect	High	[]
VLFHP:VLFLP	—	Indirect	Low	["Imprecision"]

Table S13: CINeMA for the outcome "total cholesterol" in dietary groups NMA.

Comparison	Number of studies	Nature of evidence	Confidence level	Downgrading reason
MCHP:MFHP	2	Mixed	Low	["Imprecision"]
MCHP:MFLP	4	Mixed	Very low	["Within-study bias", "Imprecision"]
MCHP:VLFHP	1	Mixed	Low	["Imprecision"]
MCLP:MFHP	1	Mixed	Low	["Imprecision"]
MCLP:MFLP	8	Mixed	Low	["Imprecision"]
MCLP:VLFLP	1	Mixed	Very low	["Imprecision", "Incoherence"]
MFHP:MFLP	18	Mixed	Low	["Imprecision"]
MFHP:VLCHP	1	Mixed	Low	["Heterogeneity"]
MFHP:VLCLP	1	Mixed	Very low	["Imprecision", "Incoherence"]
MFHP:VLFHP	1	Mixed	Low	["Imprecision"]
MFLP:VLCHP	7	Mixed	Low	["Heterogeneity"]
MFLP:VLCLP	2	Mixed	Very low	["Imprecision", "Incoherence"]
MFLP:VLFLP	2	Mixed	Low	["Imprecision"]
VLCHP:VLFLP	2	Mixed	Low	["Heterogeneity", "Incoherence"]
MCHP:MCLP	—	Indirect	Very low	["Imprecision", "Incoherence"]
MCHP:VLCHP	—	Indirect	Very low	["Within-study bias", "Imprecision", "Incoherence"]
MCHP:VLCLP	—	Indirect	Very low	["Imprecision", "Incoherence"]
MCHP:VLFLP	—	Indirect	Very low	["Within-study bias", "Imprecision", "Incoherence"]
MCLP:VLCHP	—	Indirect	Very low	["Imprecision", "Incoherence"]
MCLP:VLCLP	—	Indirect	Very low	["Imprecision", "Incoherence"]
MCLP:VLFHP	—	Indirect	Very low	["Imprecision", "Incoherence"]
MFHP:VLFLP	—	Indirect	Very low	["Imprecision", "Incoherence"]
MFLP:VLFHP	—	Indirect	Very low	["Imprecision", "Incoherence"]
VLCHP:VLCLP	—	Indirect	Very low	["Imprecision", "Incoherence"]
VLCHP:VLFHP	—	Indirect	Very low	["Imprecision", "Incoherence"]
VLCLP:VLFHP	—	Indirect	Very low	["Imprecision", "Incoherence"]
VLCLP:VLFLP	—	Indirect	Very low	["Imprecision", "Incoherence"]
VLFHP:VLFLP	—	Indirect	Very low	["Imprecision", "Incoherence"]

Figure S36: Intervention ranking chart for weight change.

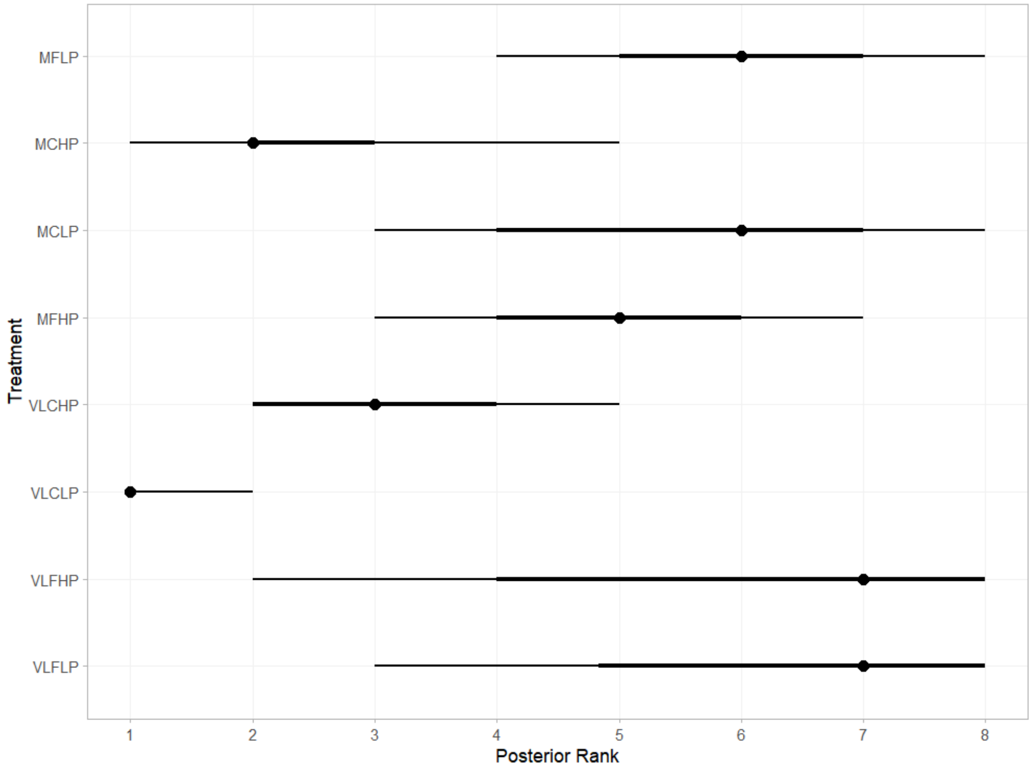

Figure S36 Intervention ranking chart for weight change. The smaller the dietary group rank value, the more effective the dietary group is in losing weight.

Figure S37-39: Network meta-analysis of blood glucose.

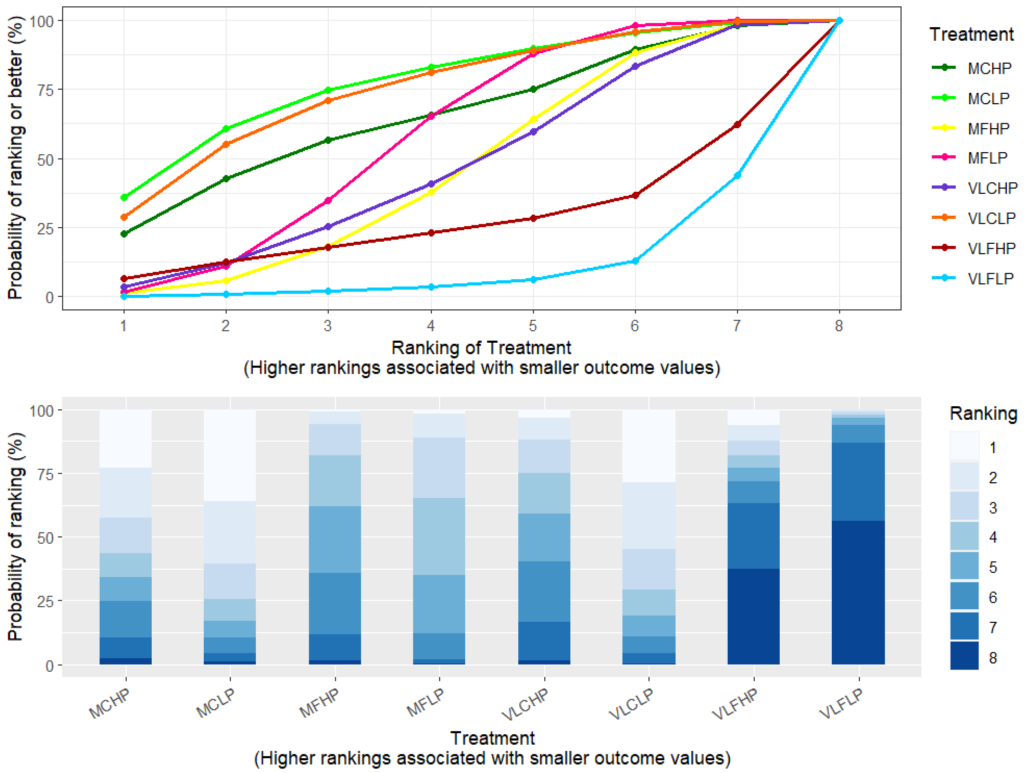

Figure S37 SUCRA and rankogram charts of blood glucose. The upper graph represents a SUCRA chart, indicating that a higher curve in the fietary group corresponds to a more effective outcome for reduction of blood glucose; the lower graph displays a rankogram chart, suggesting that lighter shades in the dietary group indicate a superior effect for reduction of blood glucose.

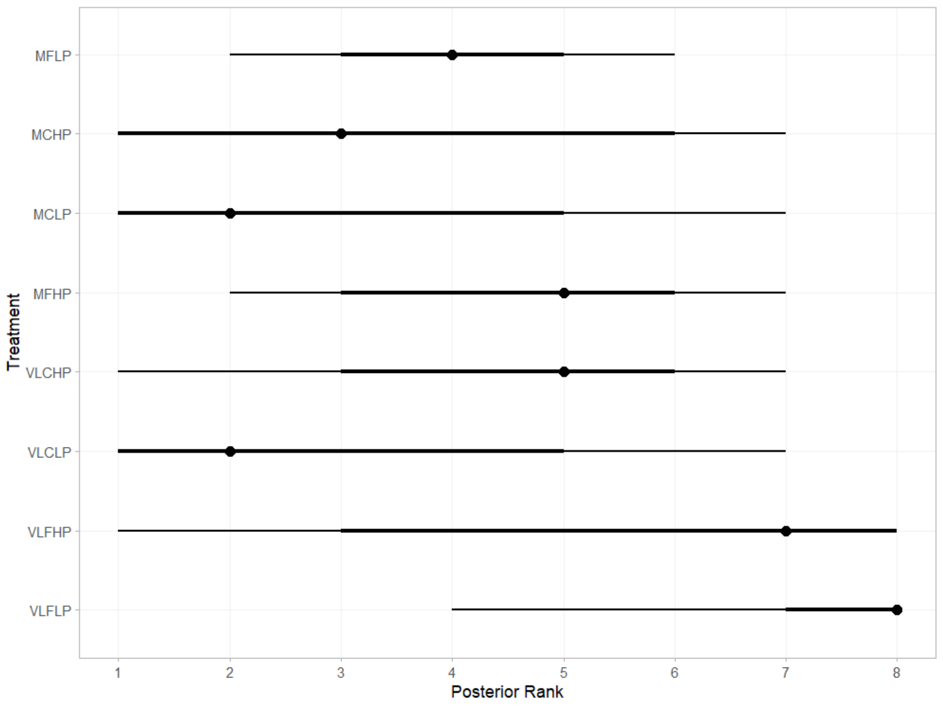

Figure S38 Intervention ranking chart for reduction of blood glucose.

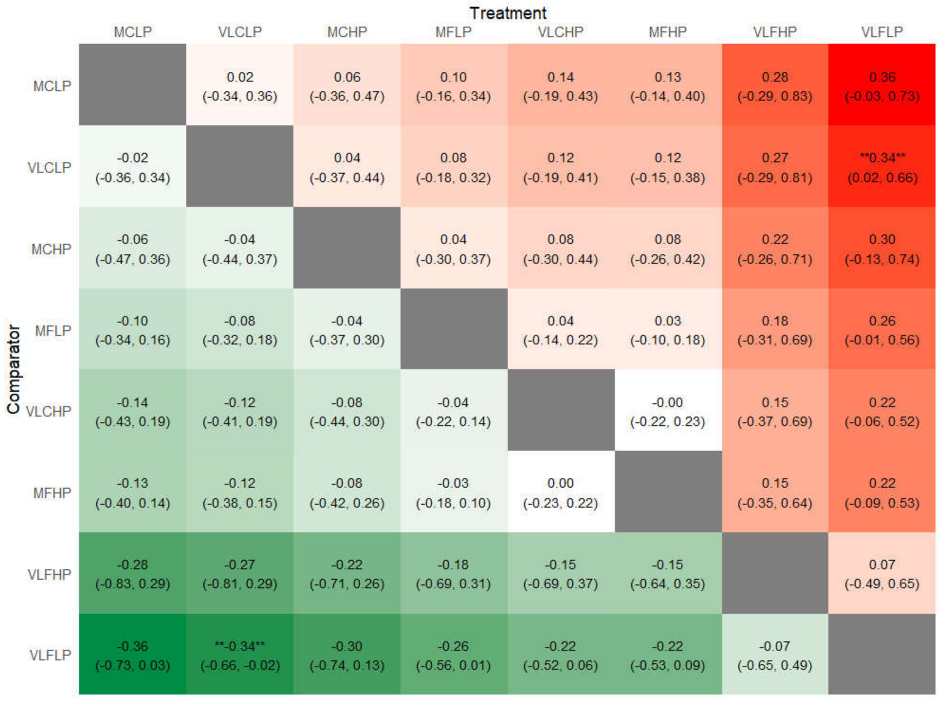

Fig.S39 Ranking list heat map of blood glucose. "Treatment" represents the intervention and "Comparator" represents the control. The symbol (**) indicates that there is a statistically significant difference between "Treatment" and "Comparator" at the 95% confidence interval level.

Figure S40-42: Network meta-analysis of systolic blood pressure.

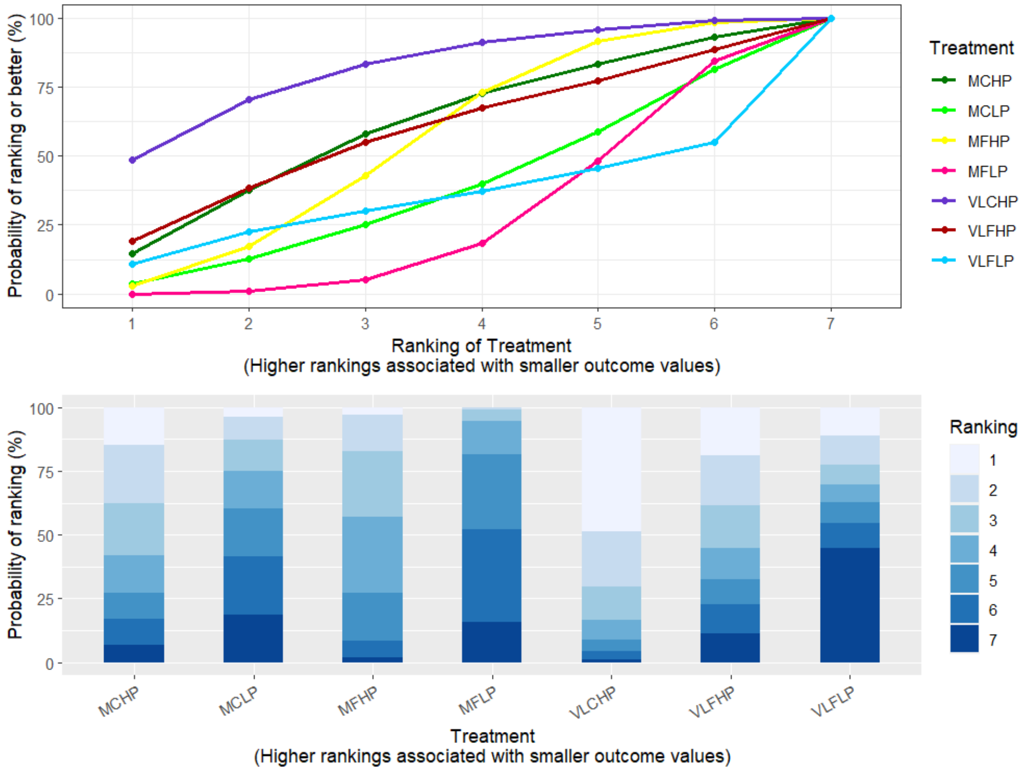

Figure S40 SUCRA and rankogram charts of systolic blood pressure. The upper graph represents a SUCRA chart, indicating that a higher curve in the dietary group corresponds to a more effective weight for reduction of systolic blood pressure; the lower graph displays a rankogram chart, suggesting that lighter shades in the dietary group indicate a superior effect for reduction of systolic blood pressure.

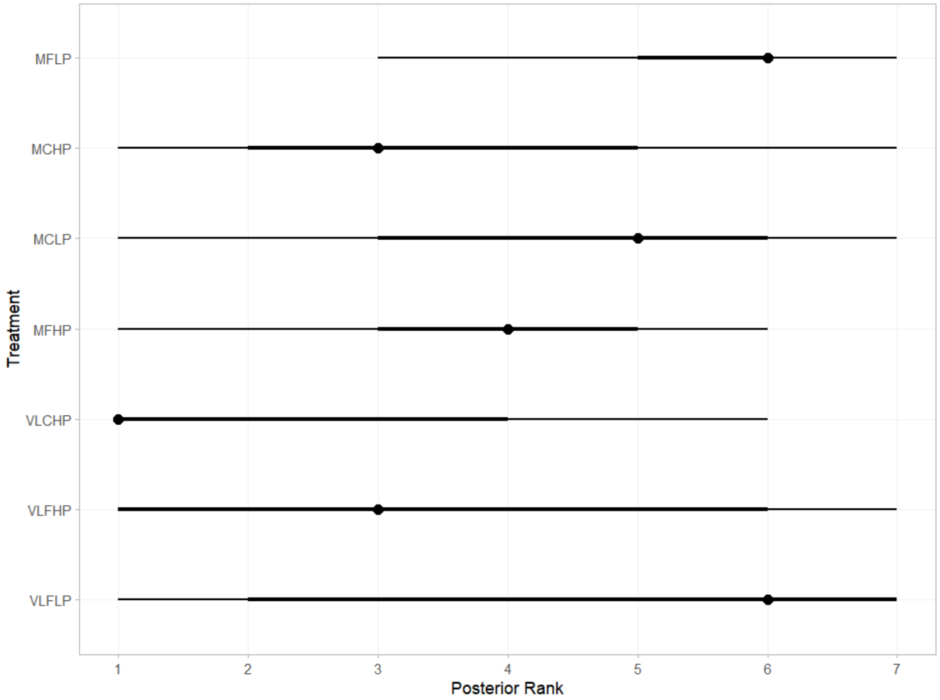

Figure S41 Intervention ranking chart for reduction of systolic blood pressure.

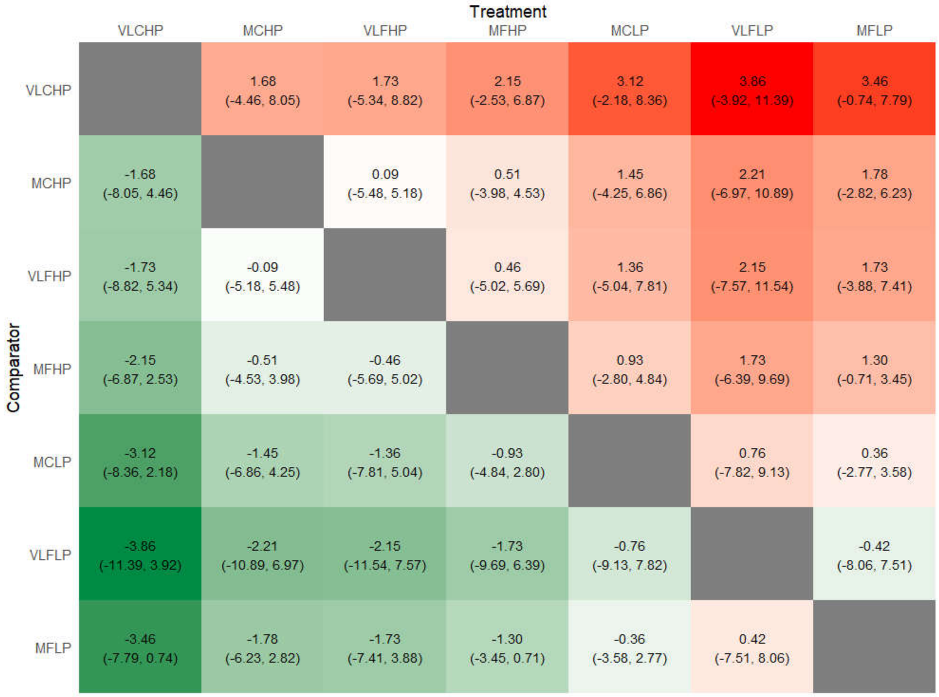

Fig.S42 Ranking list heat map of systolic blood pressure. "Treatment" represents the intervention and "Comparator" represents the control. The symbol (**) indicates that there is a statistically significant difference between "Treatment" and "Comparator" at the 95% confidence interval level.

Figure S43-45: Network meta-analysis of diastolic blood pressure.

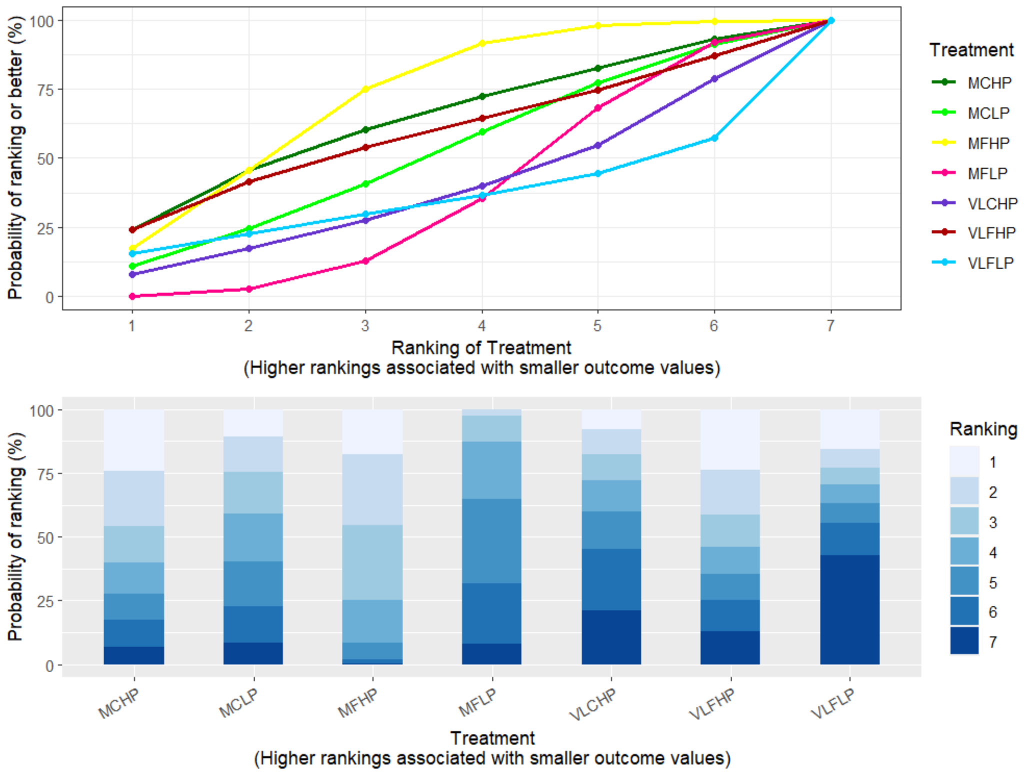

Fig.S43 SUCRA and rankogram charts of diastolic blood pressure. The upper graph represents a SUCRA chart, indicating that a higher curve in the diet group corresponds to a more effective weight for reduction of diastolic blood pressure; the lower graph displays a rankogram chart, suggesting that lighter shades in the diet group indicate a superior effect for reduction of diastolic blood pressure.

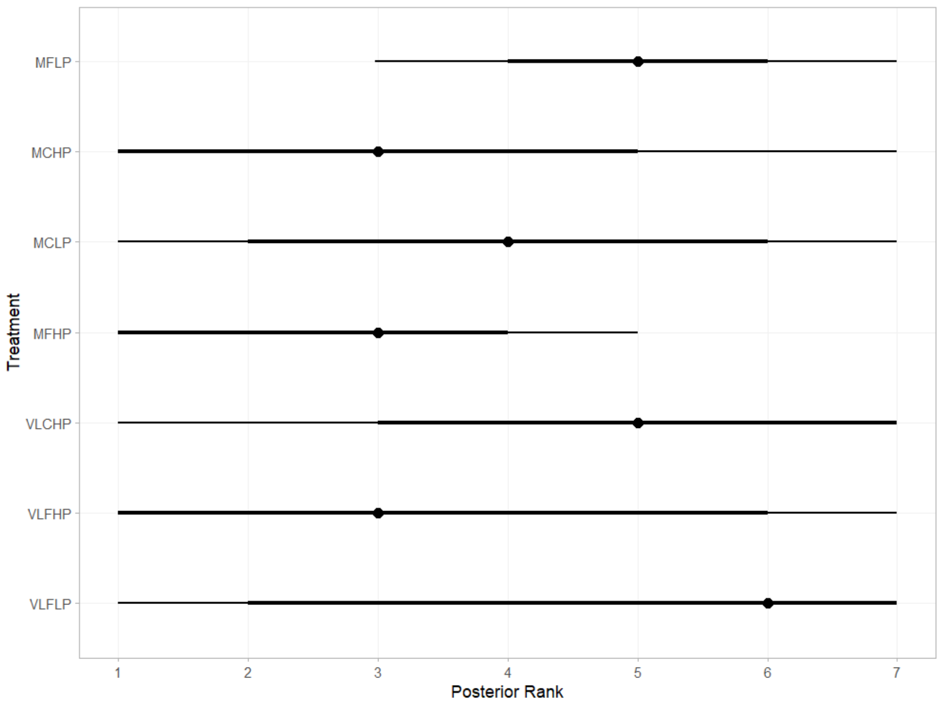

Fig.S44 Intervention ranking chart for reduction of diastolic blood pressure.

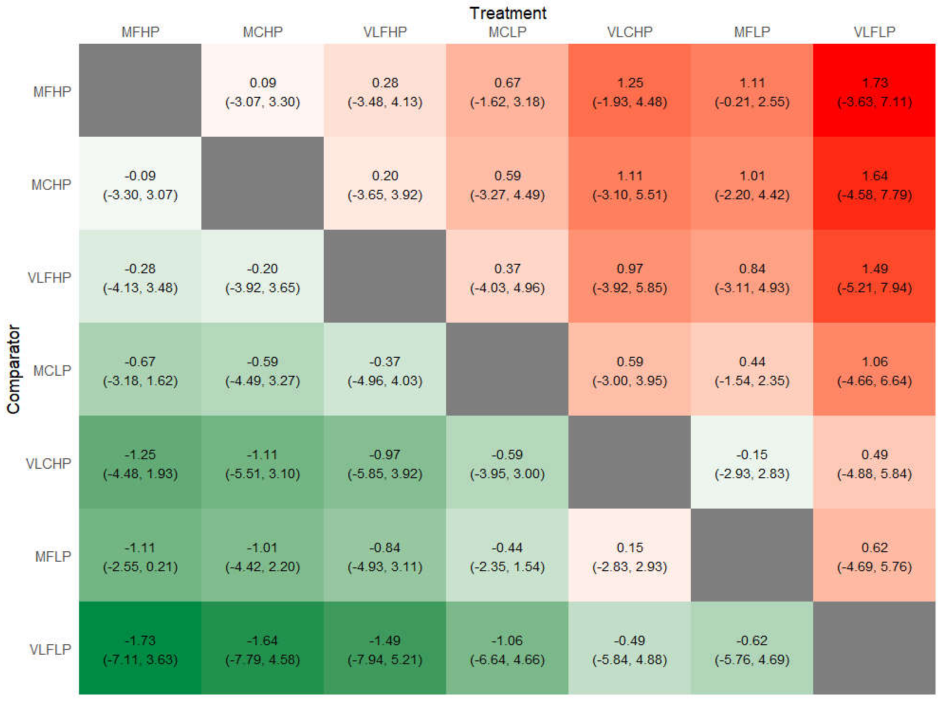

Figure S45 Ranking list heat map of diastolic blood pressure. "Treatment" represents the intervention and "Comparator" represents the control. The symbol (**) indicates that there is a statistically significant difference between "Treatment" and "Comparator" at the 95% confidence interval level.

Figure S46-57: Network meta-analysis of Blood lipids.

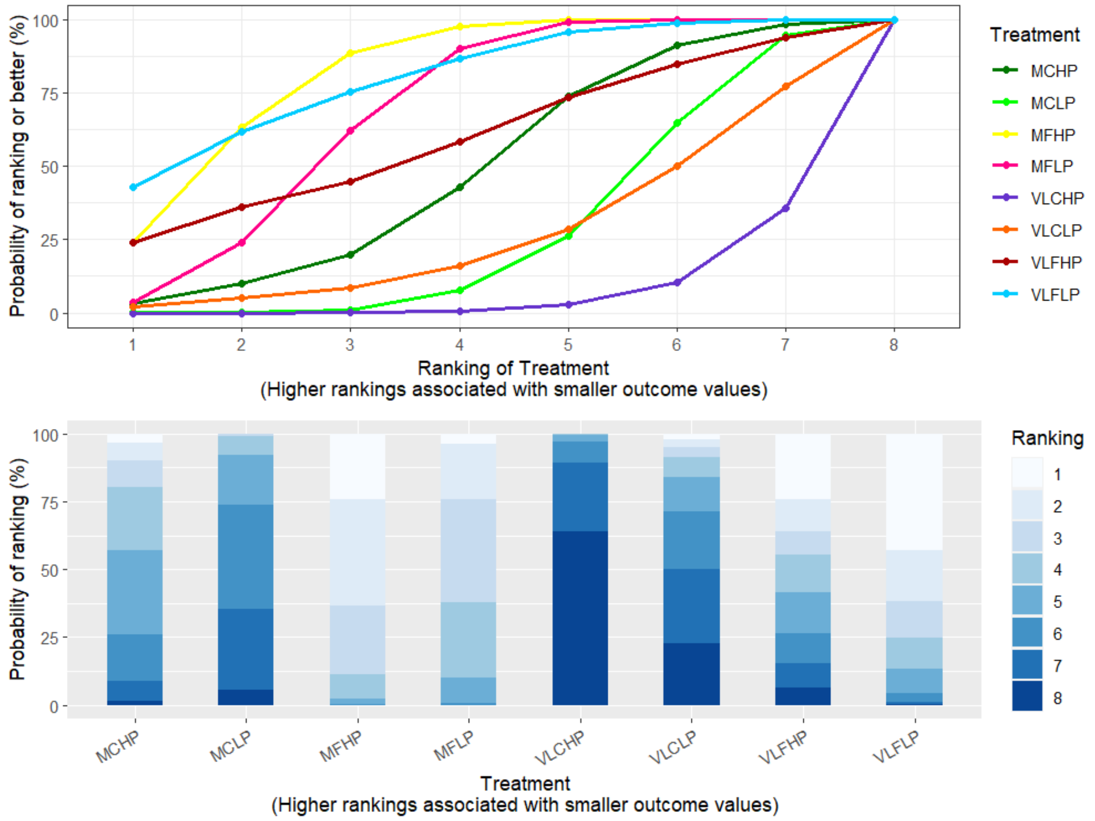

Figure S46 SUCRA and rankogram charts of HDL cholesterol. The upper graph represents a SUCRA chart, indicating that a higher curve in the diet group corresponds to a more effective outcome for reduction of HDL cholesterol; the lower graph displays a rankogram chart, suggesting that lighter shades in the diet group indicate a superior effect for reduction of HDL cholesterol.

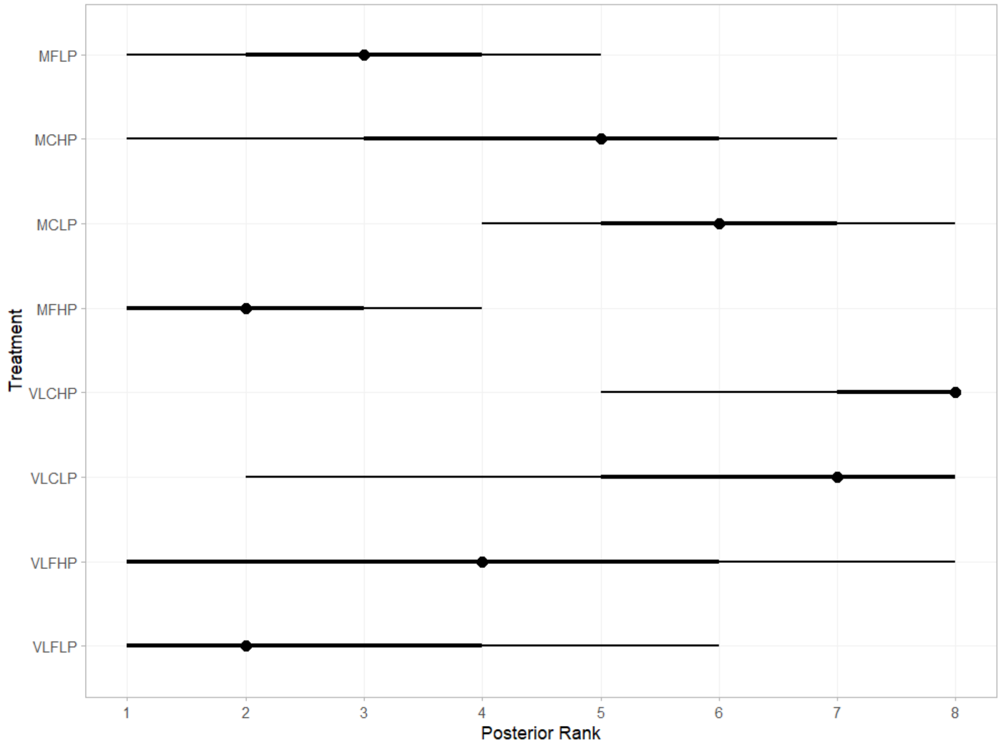

Figure S47 Intervention ranking chart for reduction of HDL cholesterol.

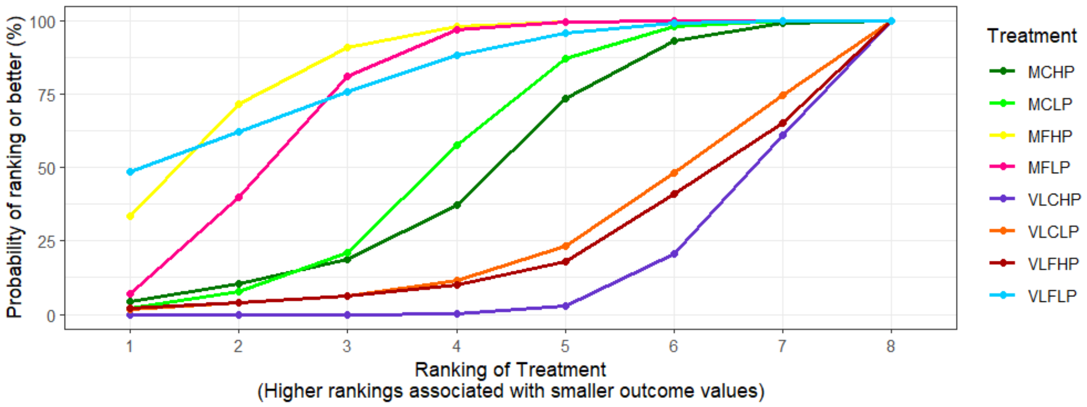

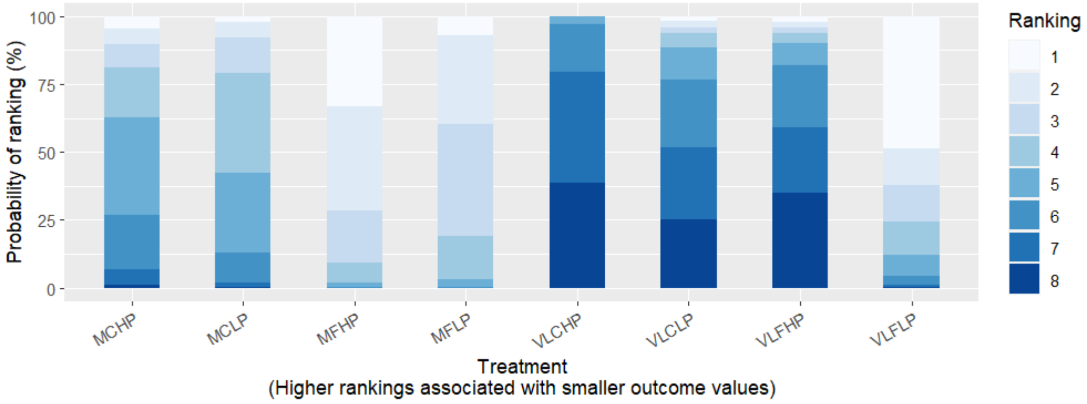

Figure S48. SUCRA and rankogram charts of LDL cholesterol. The upper graph represents a SUCRA chart, indicating that a higher curve in the diet group corresponds to a more effective outcome for reduction of LDL cholesterol; the lower graph displays a rankogram chart, suggesting that lighter shades in the diet group indicate a superior effect for reduction of LDL cholesterol.

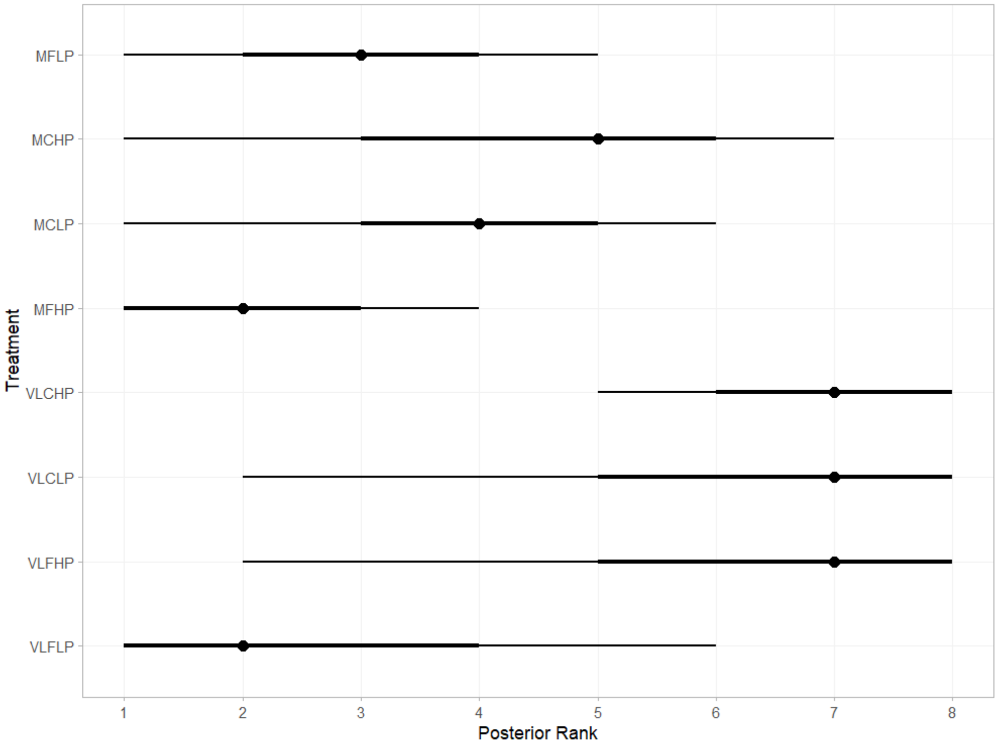

Figure S49 Intervention ranking chart for reduction of LDL cholesterol.

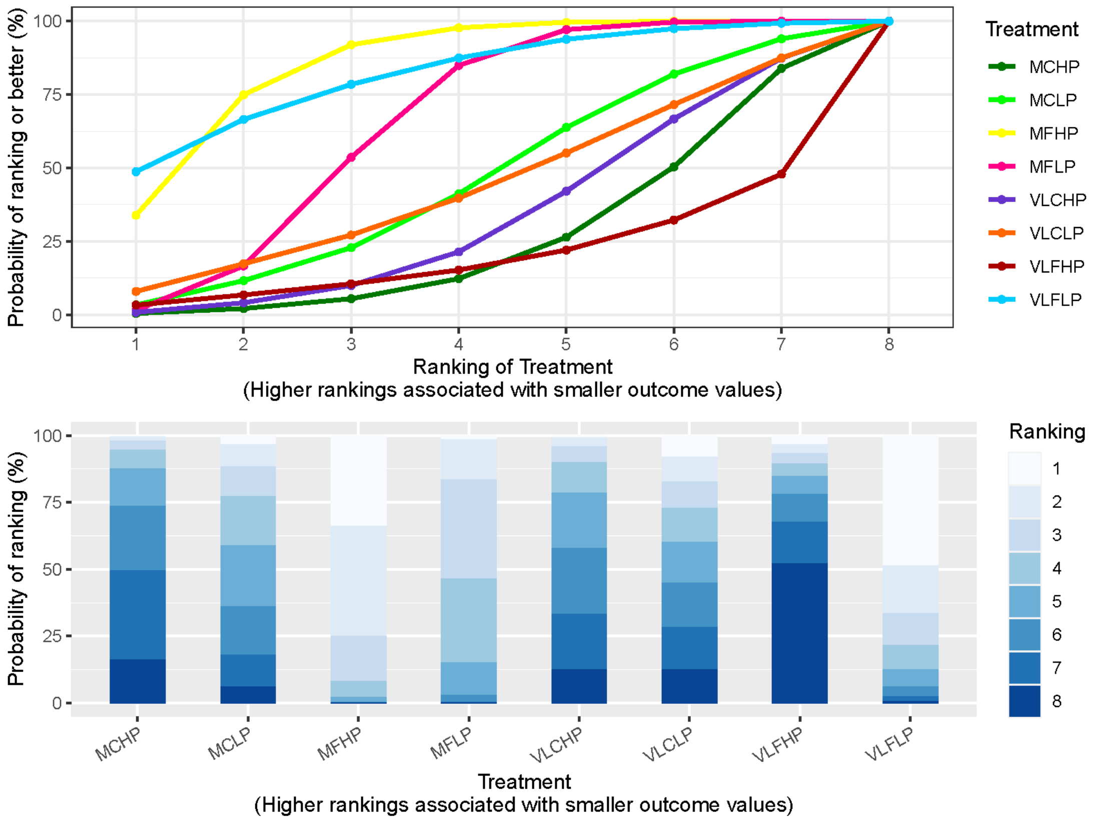

Figure S50 SUCRA and rankogram charts of total cholesterol. The upper graph represents a SUCRA chart, indicating that a higher curve in the diet group corresponds to a more effective outcome for reduction of total cholesterol; the lower graph displays a rankogram chart, suggesting that lighter shades in the diet group indicate a superior effect for reduction of total cholesterol.

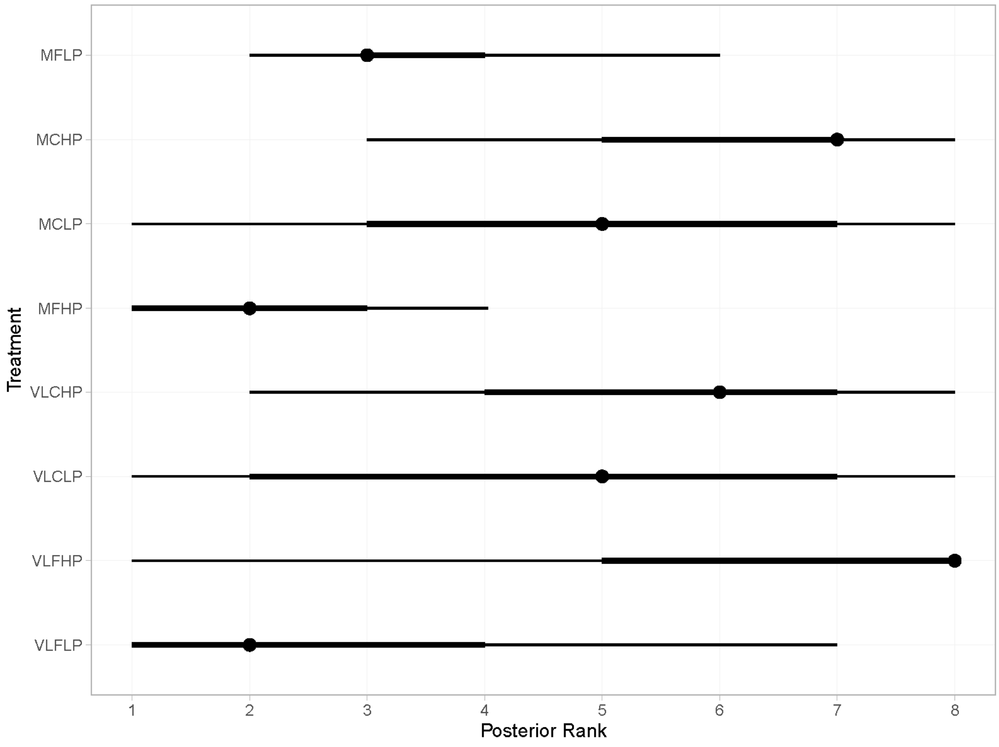

Figure S51 Intervention ranking chart for reduction of total cholesterol.

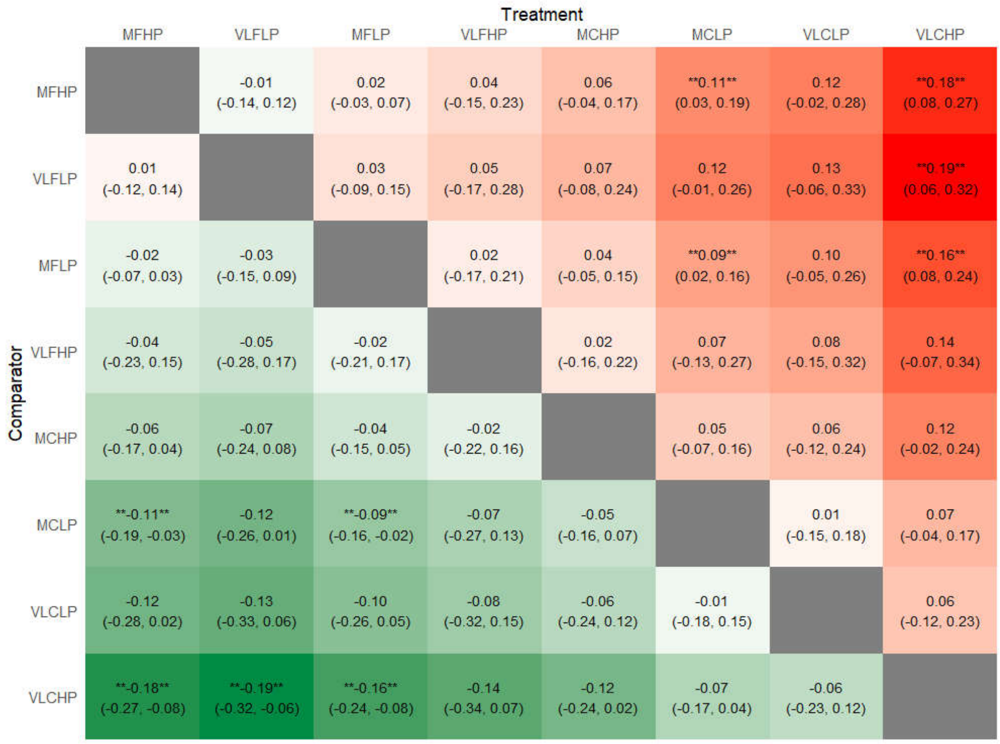

Figure S52 Ranking list heat map of HDL cholesterol. "Treatment" represents the intervention and "Comparator" represents the control. The symbol (**) indicates that there is a statistically significant difference between "Treatment" and "Comparator" at the 95% confidence interval level.

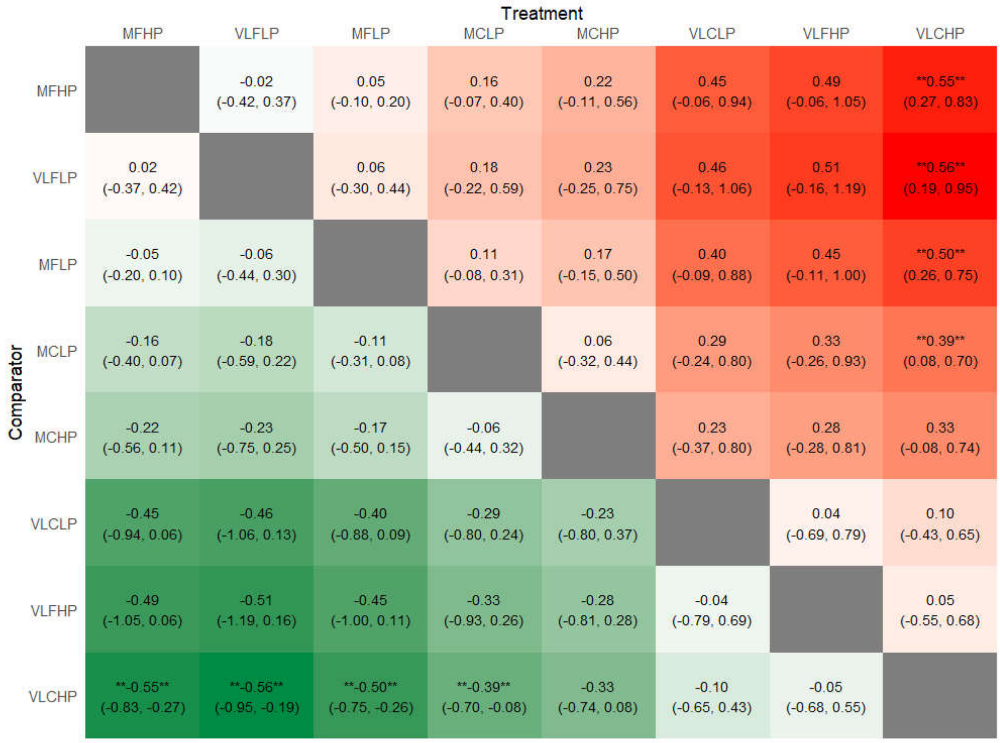

Figure S53 Ranking list heat map of LDL cholesterol. "Treatment" represents the intervention and "Comparator" represents the control. The symbol (**) indicates that there is a statistically significant difference between "Treatment" and "Comparator" at the 95% confidence interval level.

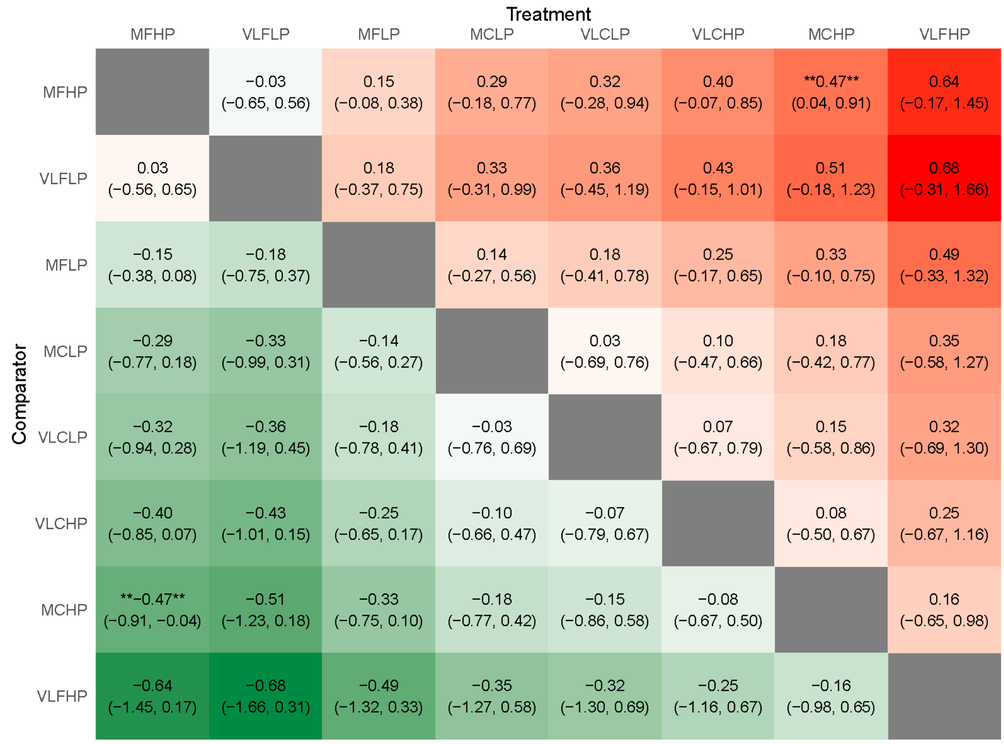

Figure 54 Ranking list heat map of total cholesterol. "Treatment" represents the intervention and "Comparator" represents the control. The symbol (**) indicates that there is a statistically significant difference between "Treatment" and "Comparator" at the 95% confidence interval level.

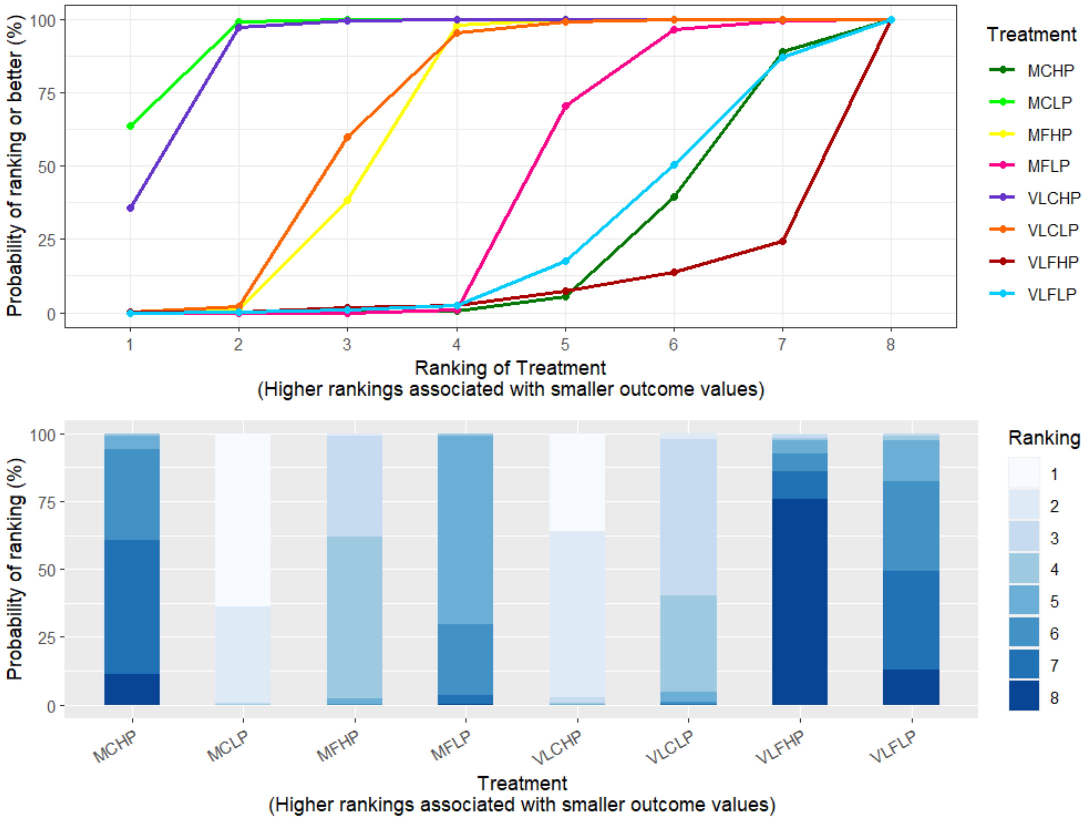

Figure S55 SUCRA and rankogram charts of triglyceride. The upper graph represents a SUCRA chart, indicating that a higher curve in the diet group corresponds to a more effective outcome for reduction of triglyceride; the lower graph displays a rankogram chart, suggesting that lighter shades in the diet group indicate a superior effect for reduction of triglyceride.

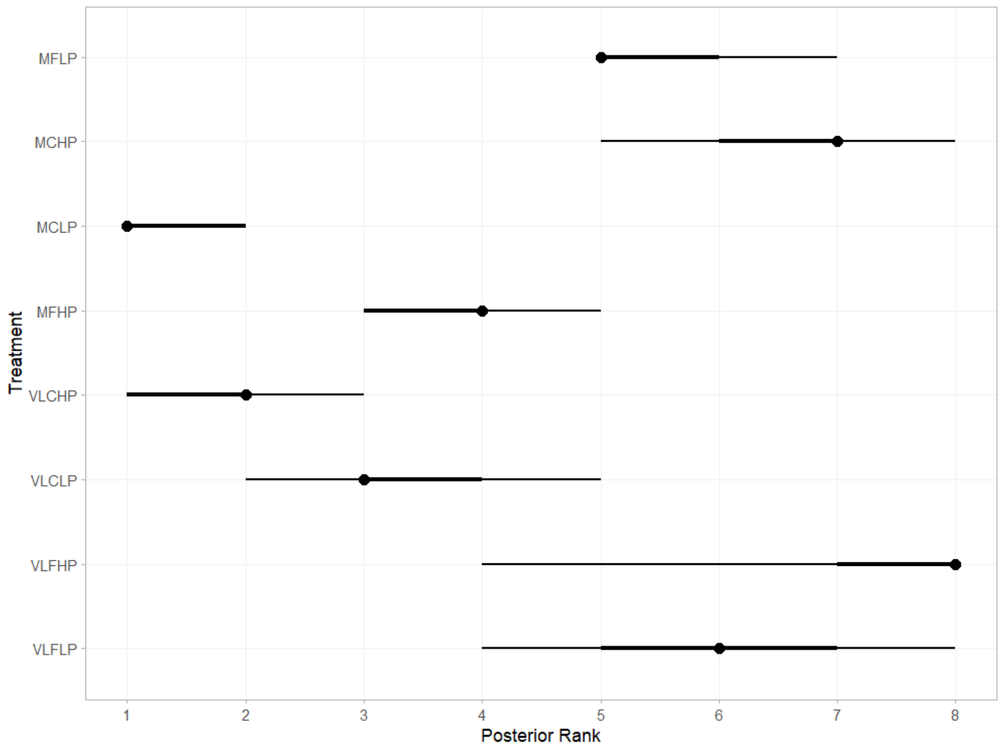

Figure S56 Intervention ranking chart for reduction of Triglyceride.

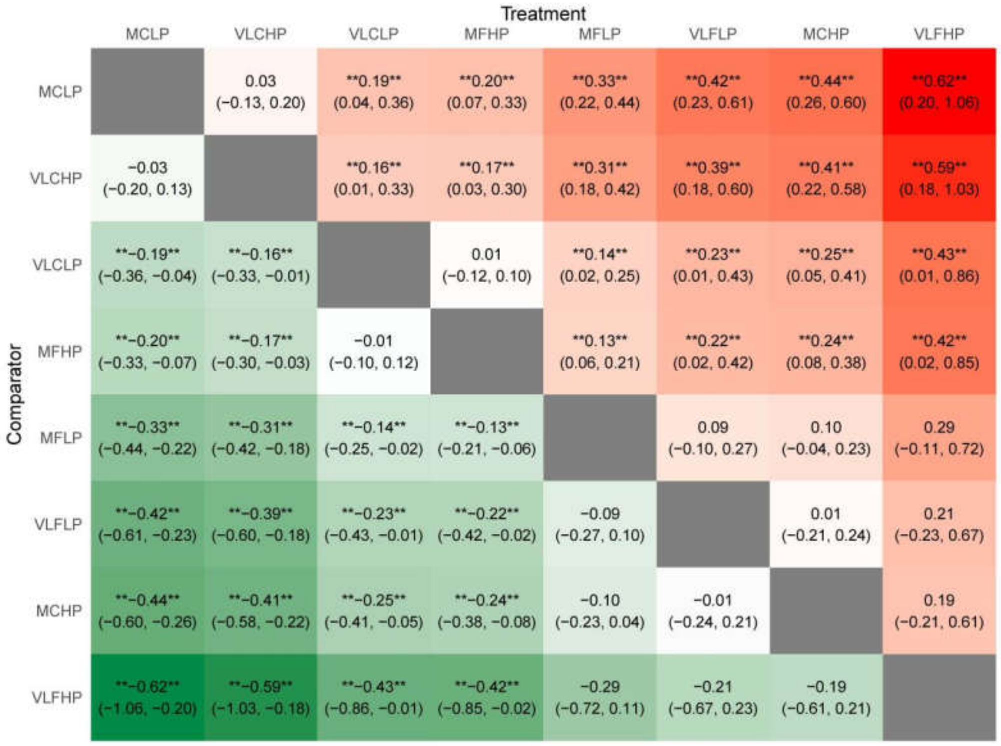

Figure S57 Ranking list heat map of triglyceride. "Treatment" represents the intervention and "Comparator" represents the control. The symbol (**) indicates that there is a statistically significant difference between "Treatment" and "Comparator" at the 95% confidence interval level.

Table S14: Values of network meta regression coefficients for different covariates in different outcomes.

Outcome / Covariate	Weight	Blood glucose	SBP	DBP	HDL	LDL	Triglyceride	Cholesterol
Year	0.54(-0.79,1.93)	0.18(-0.077, 0.41)	0.047(-4.08, 3.98)	-0.87(-3.74, 1.86)	-0.0068(-0.096, 0.080)	0.18(-0.074, 0.44)	0.053(-0.080, 0.19)	0.10(-0.16, 0.39)
Funding	0.52(-0.66,1.68)	0.028(-0.18, 0.26)	-1.34(-4.57, 2.02)	-0.40(-2.50, 1.82)	0.025(-0.049, 0.096)	0.056(-0.16, 0.28)	0.011(-0.12, 0.14)	0.056(-0.21, 0.32)
Country	-2.44(-5.81, 0.87)	-0.22(-0.94, 0.49)	NA	NA	-0.0083(-1.25, 1.24)	-0.07(-0.85, 0.72)	-0.21(-0.86, 0.42)	-0.081(-0.36, 0.21)
Calorie	0.18(-1.12,1.44)	0.10(-0.14, 0.33)	1.56(-1.88, 4.92)	0.39(-1.94, 2.67)	-0.0084(-0.089, 0.073)	-0.16(-0.40, 0.07)	-0.047(-0.17, 0.079)	-0.081(-0.36, 0.21)
Time	0.71(-0.34,1.76)	0.21(-0.0043, 0.42)	1.89(-1.21, 5.11)	0.90(-1.07, 2.93)	0.055(-0.014, 0.12)	0.033(-0.18, 0.25)	0.0059(-0.13, 0.14)	0.072(-0.17, 0.31)
Sample	0.59(-0.64,1.84)	0.068(-0.16, 0.28)	0.87(-2.13, 3.91)	0.34(-1.42, 2.21)	-0.0006(-0.065, 0.064)	0.024(-0.18, 0.22)	0.0094(-0.15, 0.17)	-0.016(-0.24, 0.21)
Activity	0.56(-0.74,1.88)	0.21(-0.021, 0.43)	-1.55(-5.11, 1.78)	-0.52(-2.83, 1.66)	-0.056(-0.14, 0.025)	-0.0088(-0.26, 0.25)	-0.045(-0.17, 0.086)	-0.14(-0.41, 0.14)

The number is the mean (with 95% confidence interval) of regression coefficient. NA=not applicable. Weight: weight change; SBP: systolic blood pressure; DBP: diastolic blood pressure; HDL: high density lipoprotein; LDL: low density lipoprotein; Cholesterol: total cholesterol; Year: publication year; Funding: funding source; Country: country in which the study was conducted; Calorie: calorie restriction; Time: duration time of intervention period; Sample: study sample size; Activity: physical activity.

Figure S58-65: Ranking plots of each outcome in subgroups.

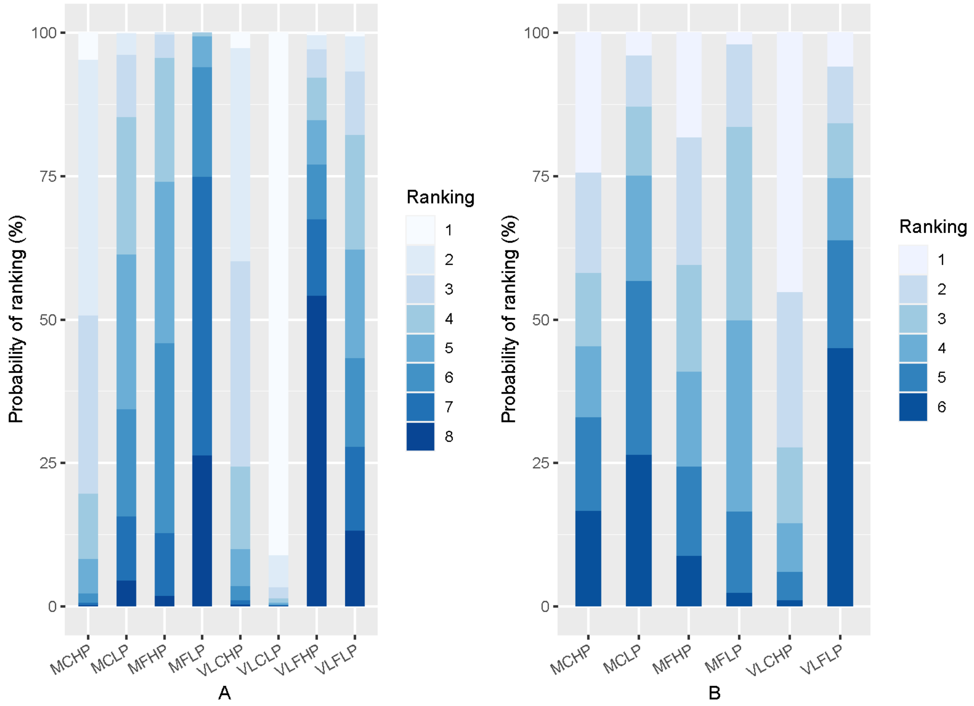

Figure S58 Ranking plot of weight change in subgroups. A, intervention period ≤ 6 months. B, intervention period > 6 months.

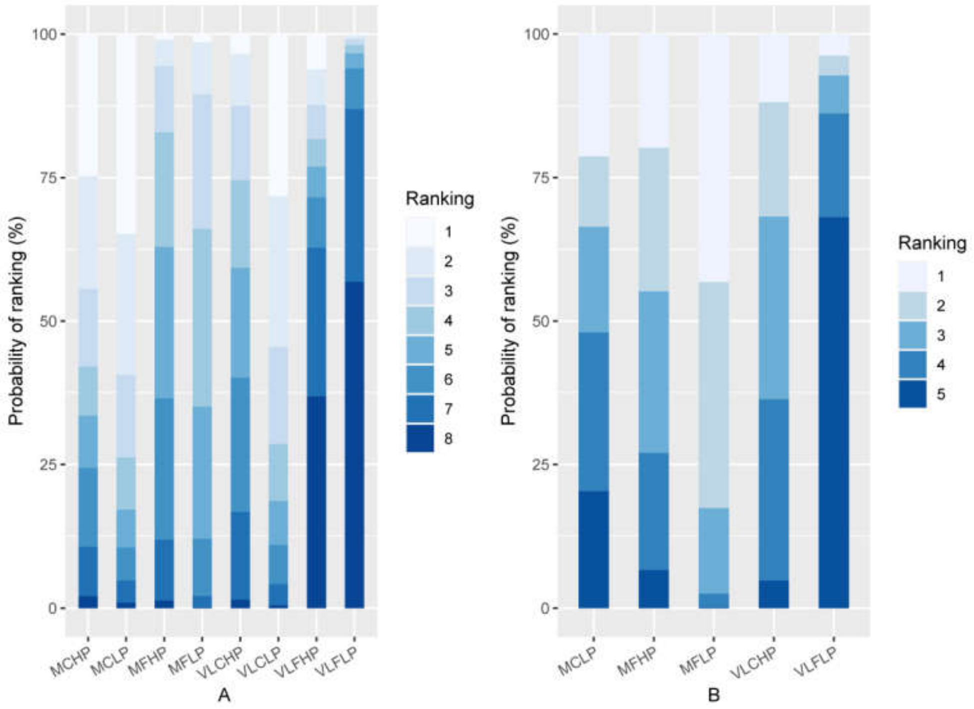

Figure S59 Ranking plot of blood glucose in subgroups. A, intervention period ≤ 6 months. B, intervention period > 6 months.

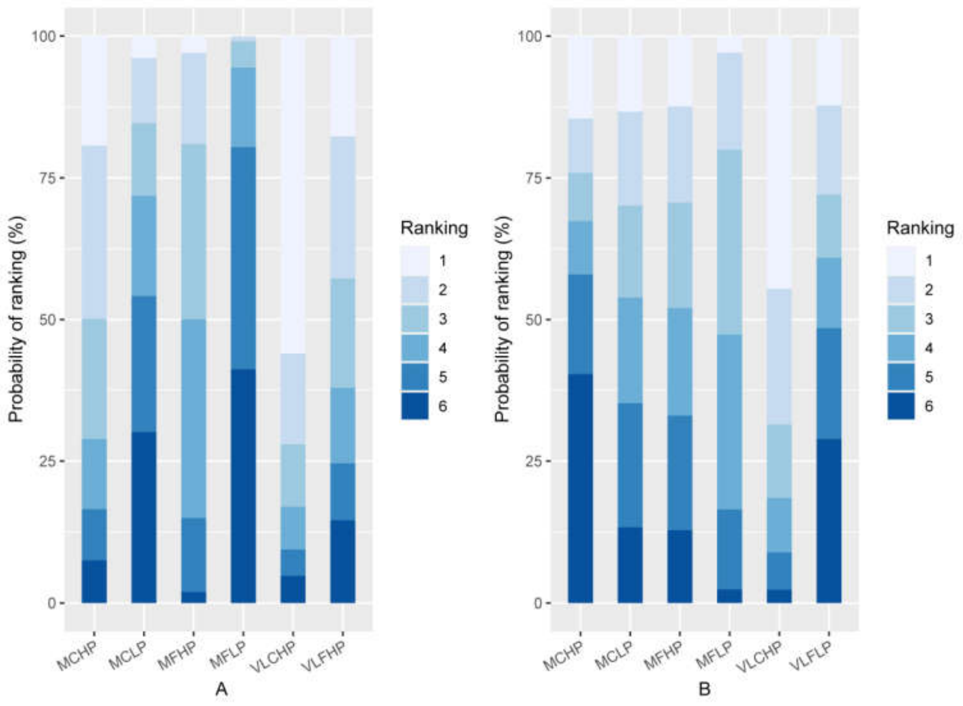

Figure S60 Ranking plot of systolic blood pressure in subgroups. A, intervention period ≤ 6 months. B, intervention period > 6 months.

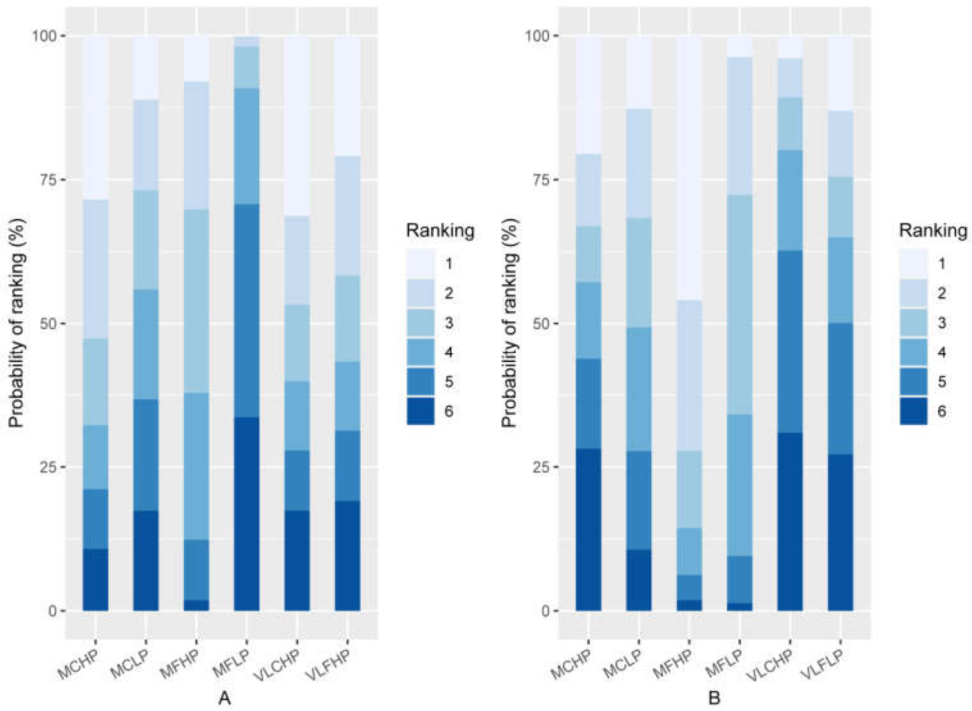

Figure S61 Ranking plot of diastolic blood pressure in subgroups. A, intervention period ≤ 6 months. B, intervention period > 6 months.

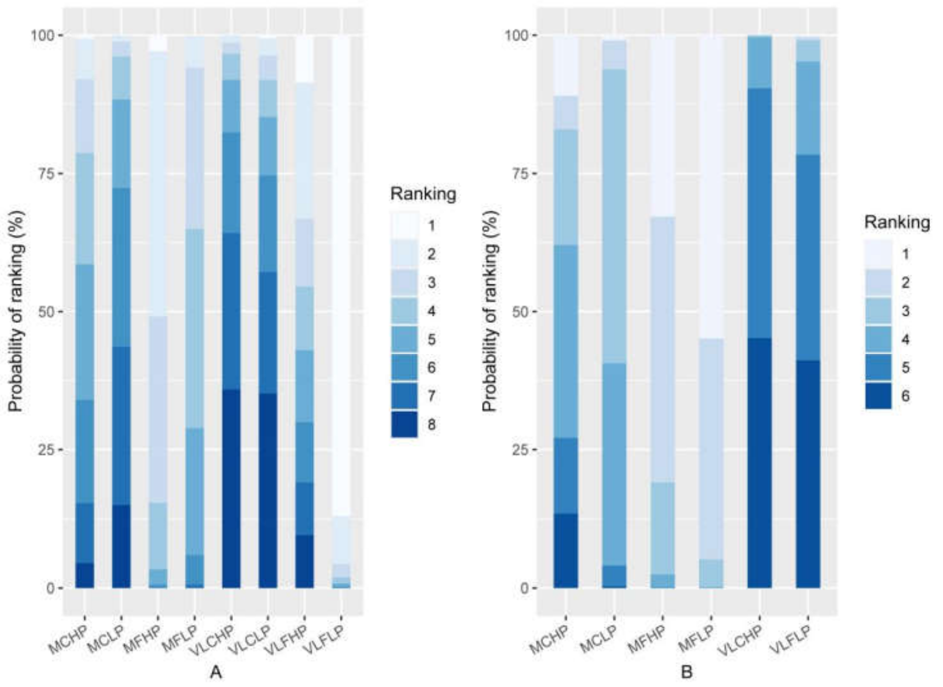

Figure S62 Ranking plot of HDL cholesterol in subgroups. A, intervention period ≤ 6 months. B, intervention period > 6 months.

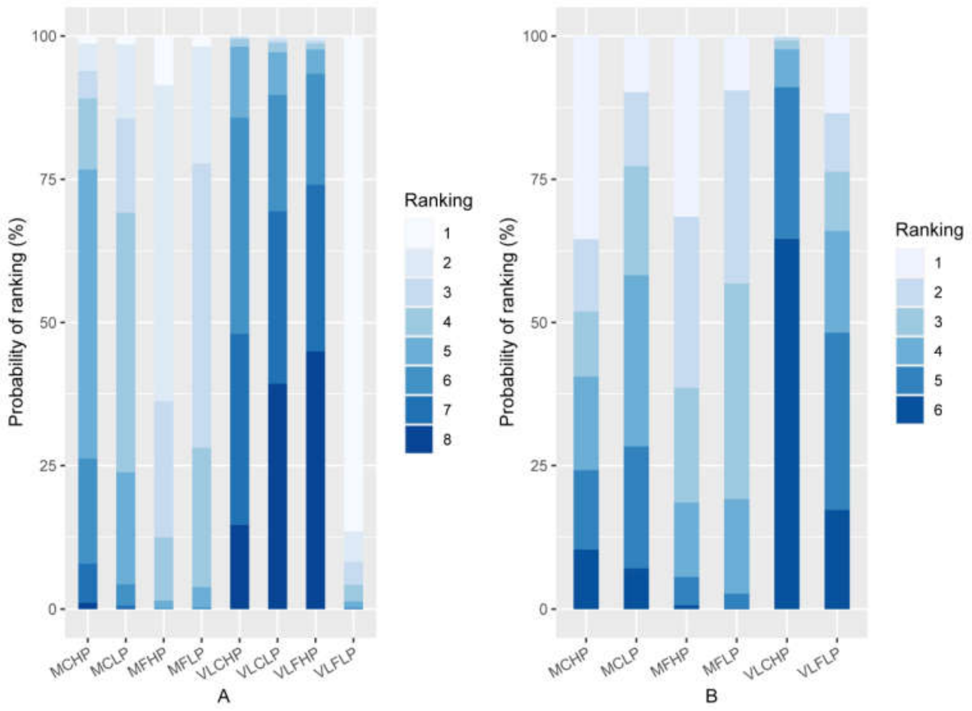

Figure S63 Ranking plot of LDL cholesterol in subgroups. A, intervention period ≤ 6 months. B, intervention period > 6 months.

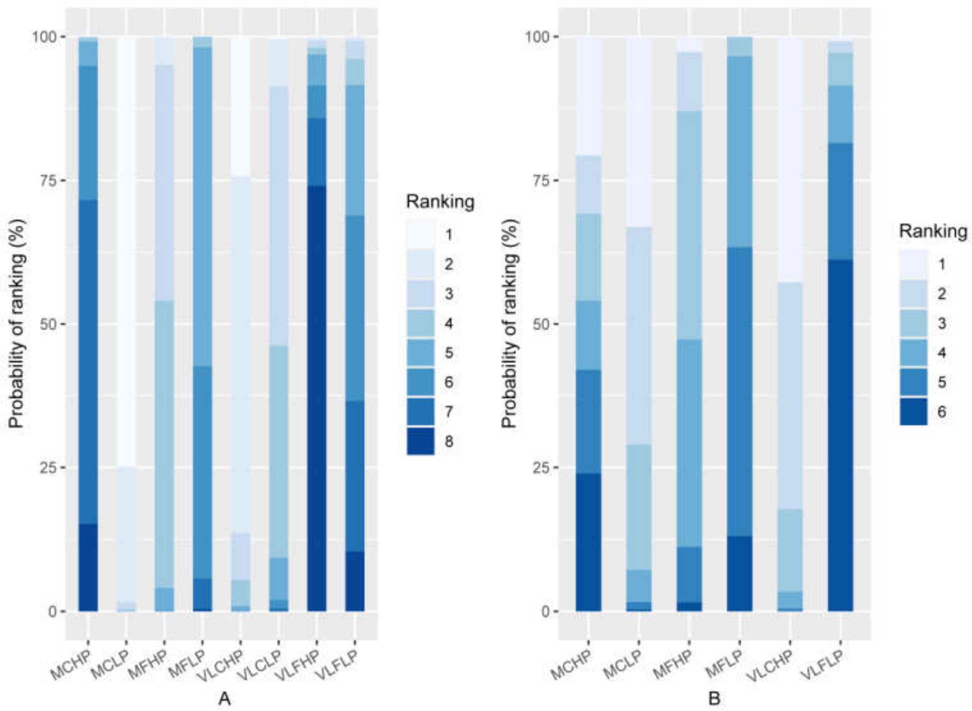

Figure S64 Ranking plot of triglyceride in subgroups. A, intervention period ≤ 6 months. B, intervention period > 6 months.

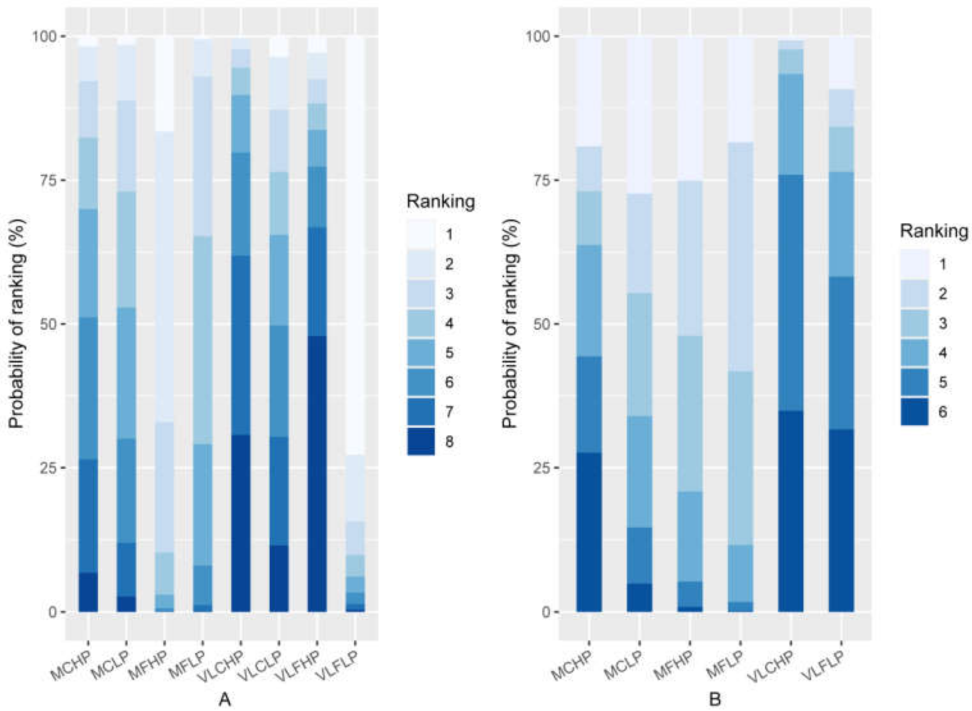

Figure S65 Ranking plot of total cholesterol in subgroups. A, intervention period ≤ 6 months. B, intervention period > 6 months.

Figure S66-73: Ranking list heat maps of each outcome in subgroups.

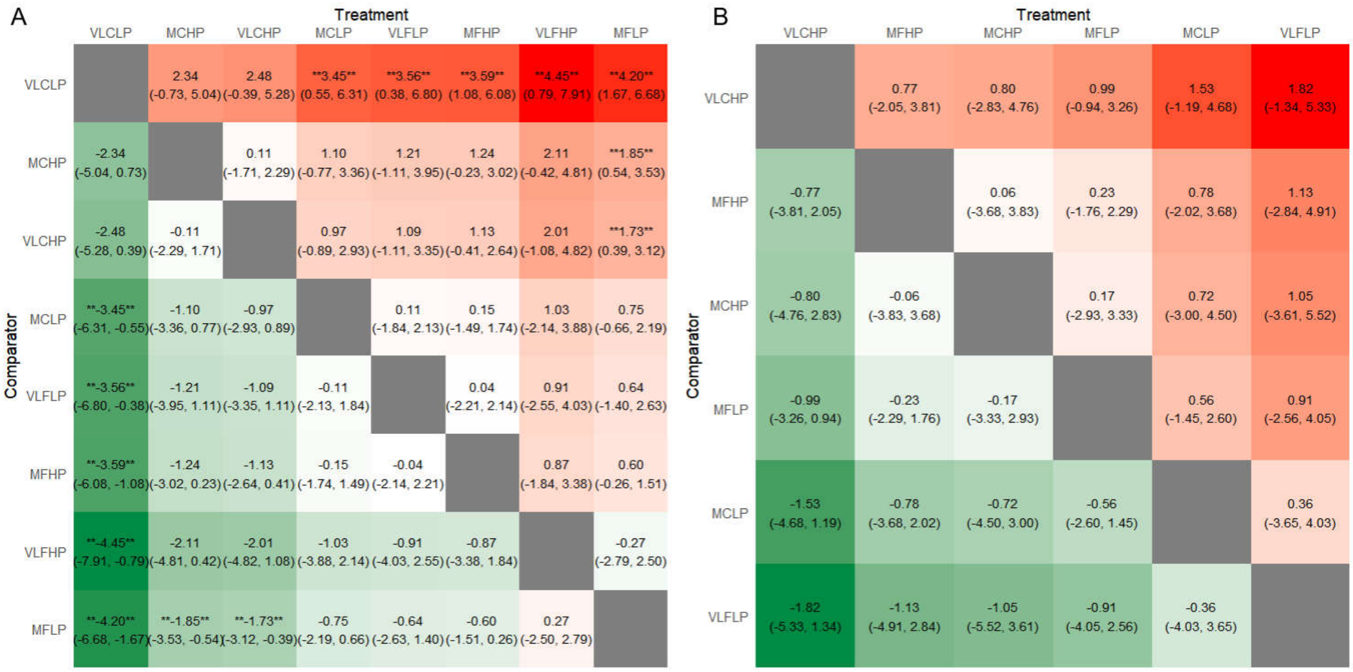

Figure S66 Ranking list heat map of weight change in subgroups (mean difference and 95% credible interval). A, intervention period ≤ 6 months. B, intervention period > 6 months.

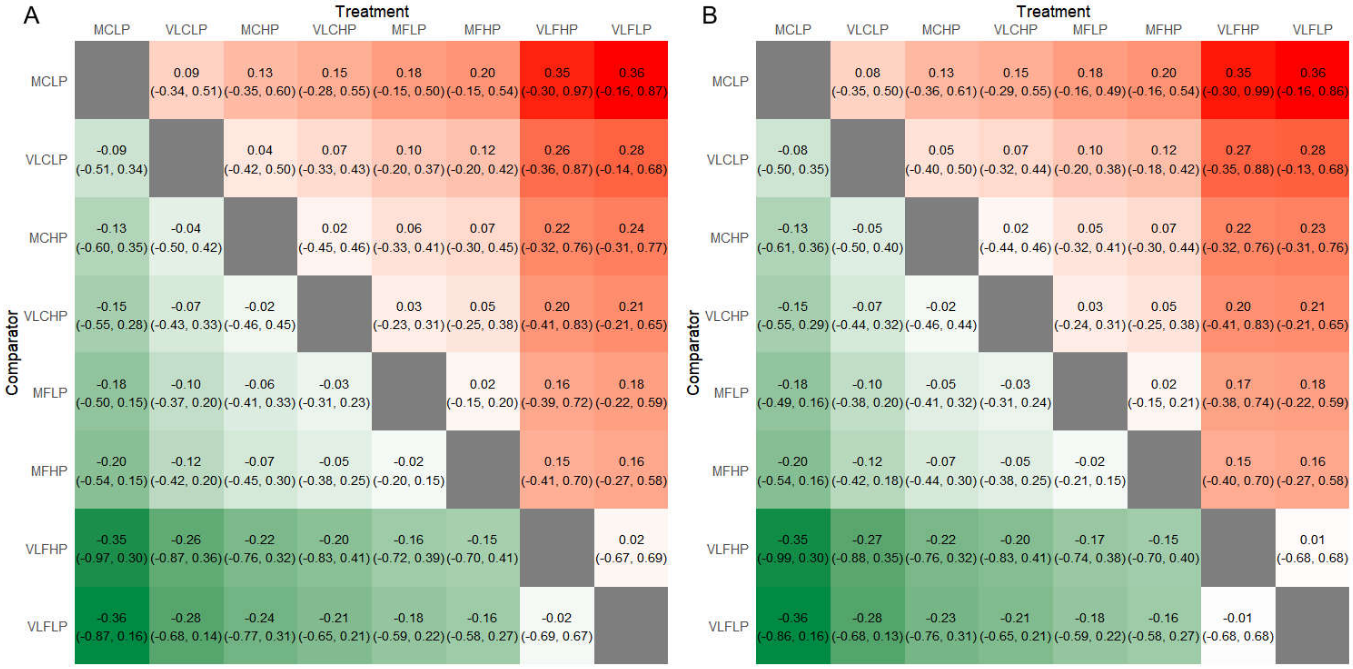

Figure S67 Ranking list heat map of blood glucose in subgroups (mean difference and 95% credible interval). A, intervention period ≤ 6 months. B, intervention period > 6 months.

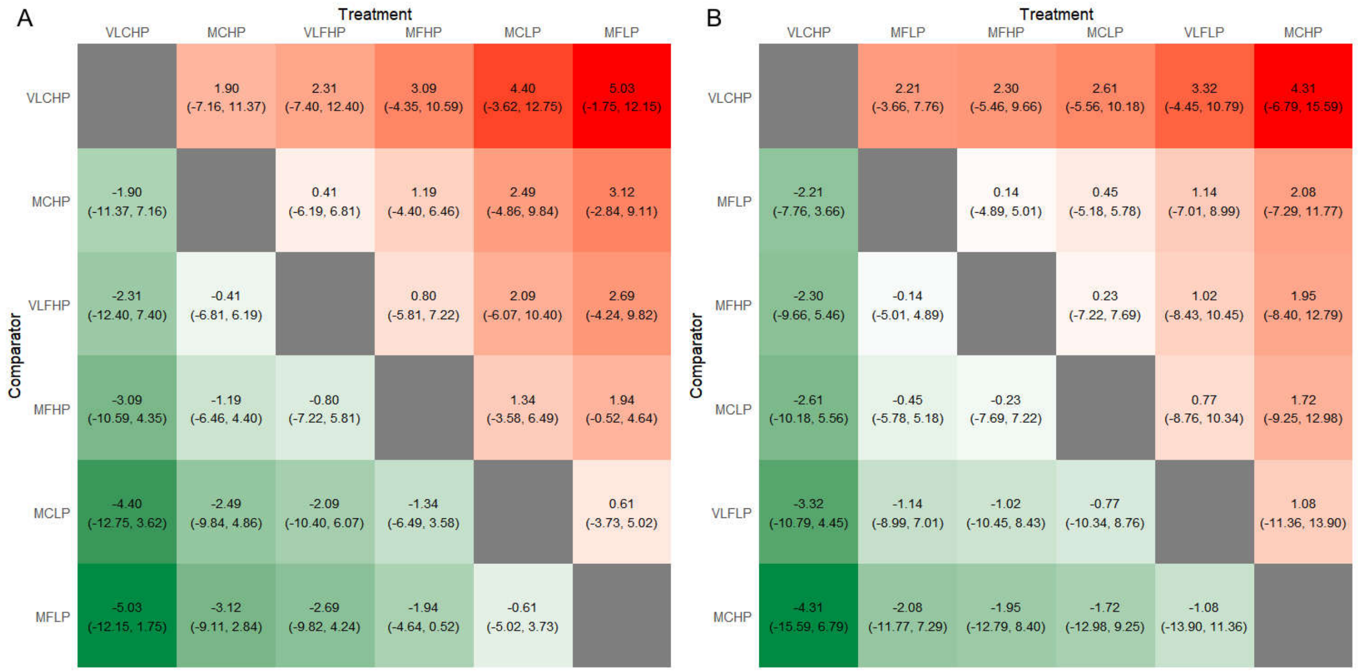

Figure S68 Ranking list heat map of systolic blood pressure in subgroups (mean difference and 95% credible interval). A, intervention period ≤ 6 months. B, intervention period > 6 months.

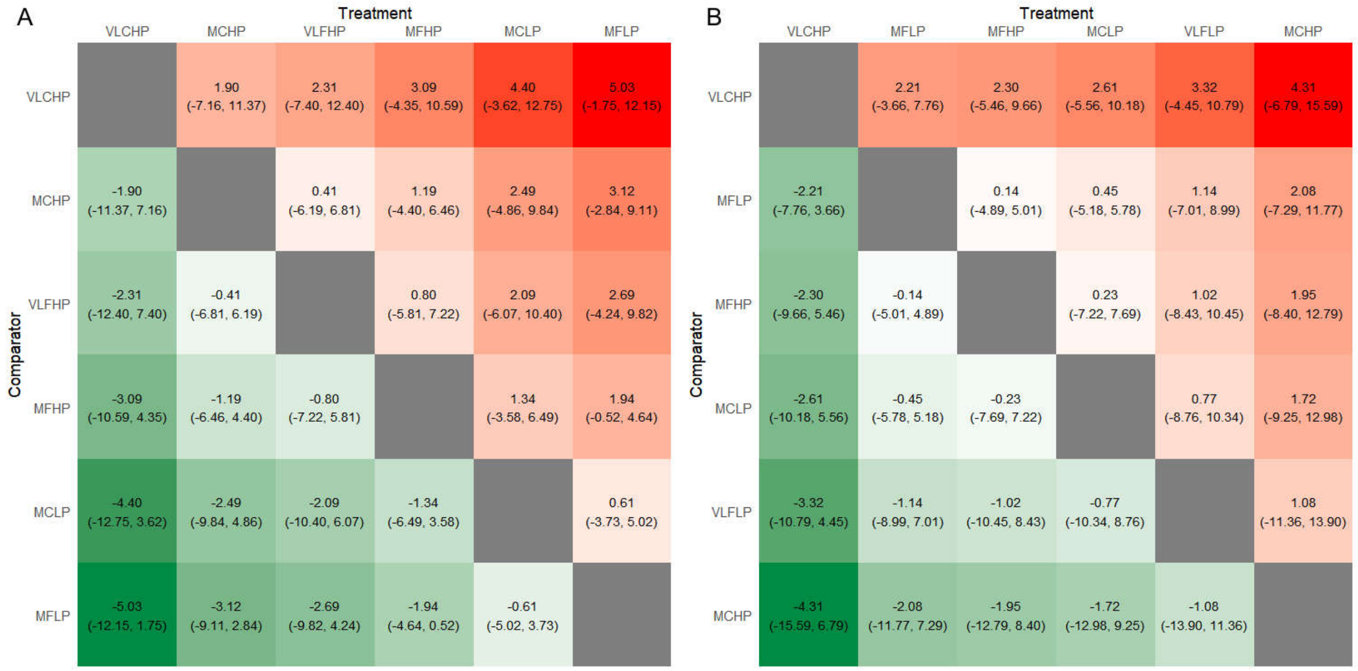

Figure S69 Ranking list heat map of diastolic blood pressure in subgroups (mean difference and 95% credible interval). A, intervention period ≤ 6 months. B, intervention period > 6 months.

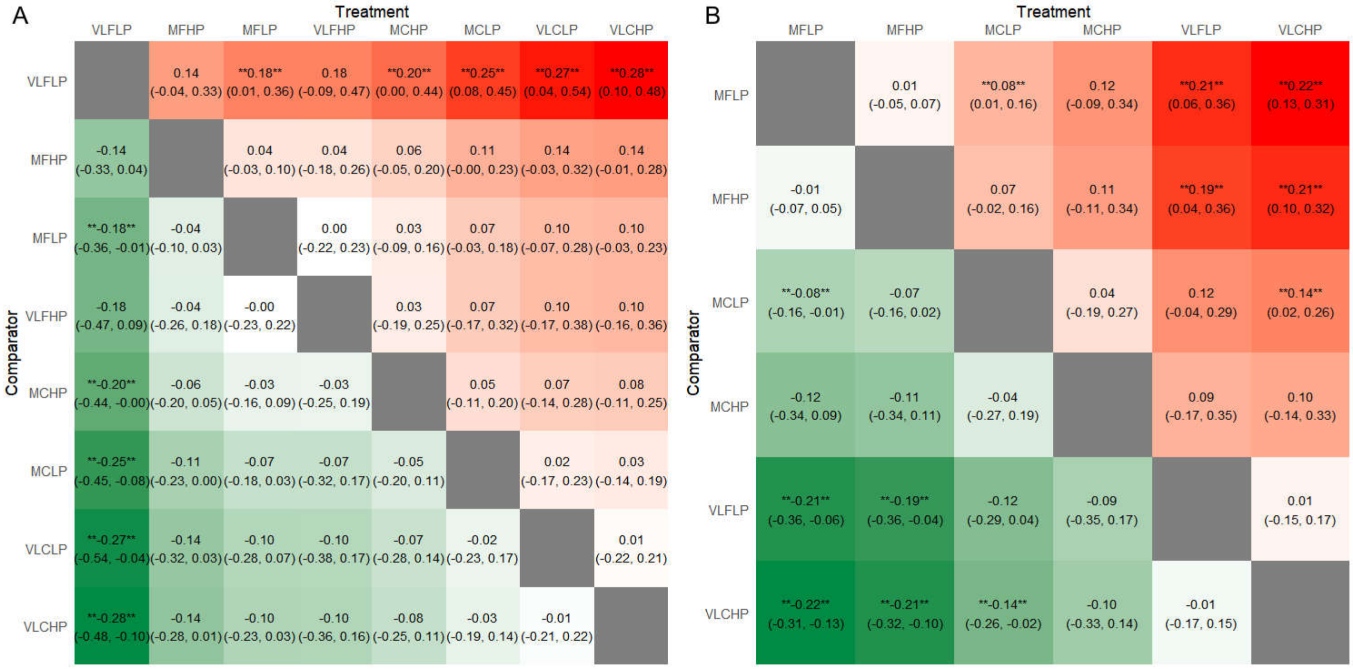

Figure S70 Ranking list heat map of HDL cholesterol in subgroups (mean difference and 95% credible interval). A, intervention period ≤ 6 months. B, intervention period > 6 months.

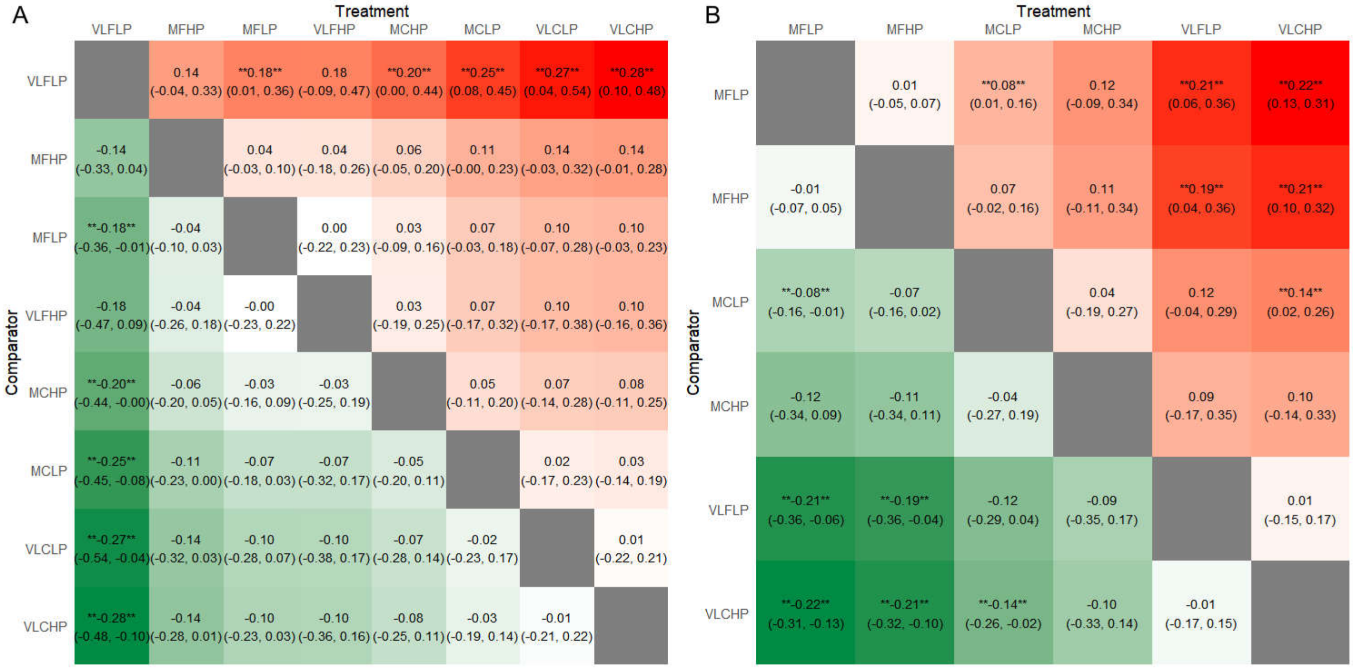

Figure S71 Ranking list heat map of LDL cholesterol in subgroups (mean difference and 95% credible interval). A, intervention period ≤ 6 months. B, intervention period > 6 months.

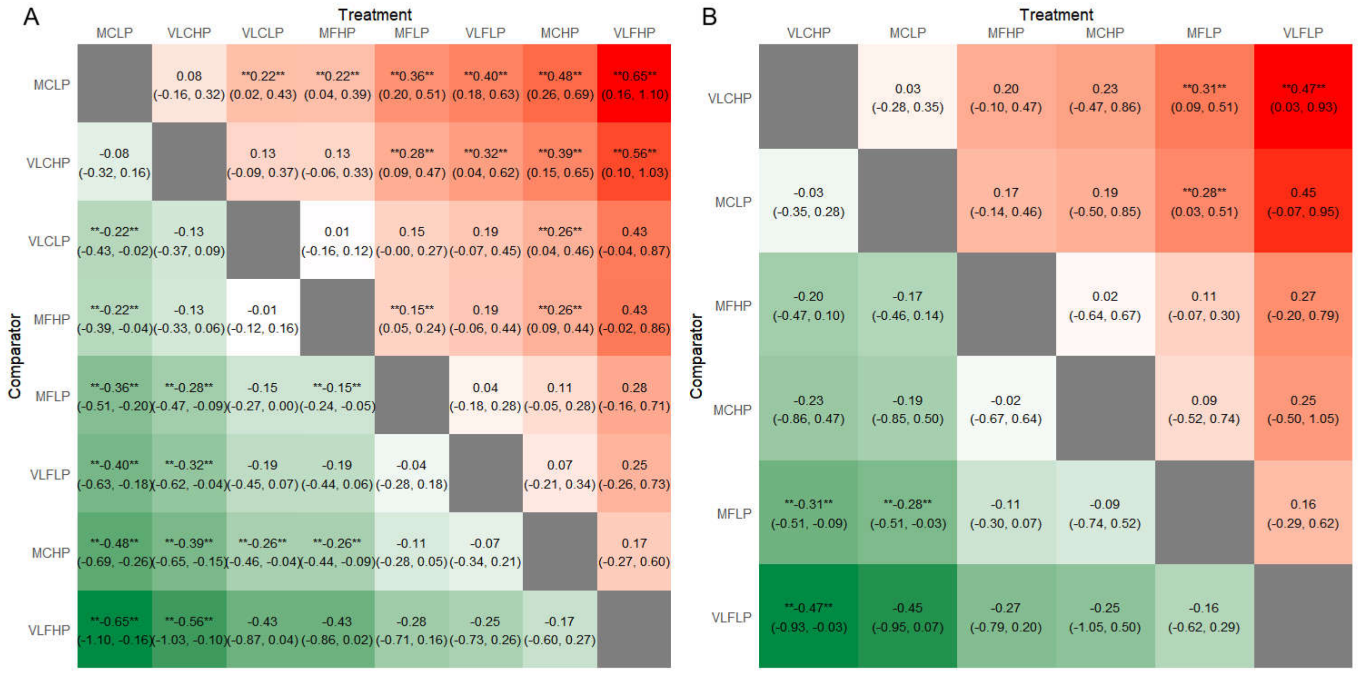

Figure S72 Ranking list heat map of triglyceride in subgroups (mean difference and 95% credible interval). A, intervention period ≤ 6 months. B, intervention period > 6 months.

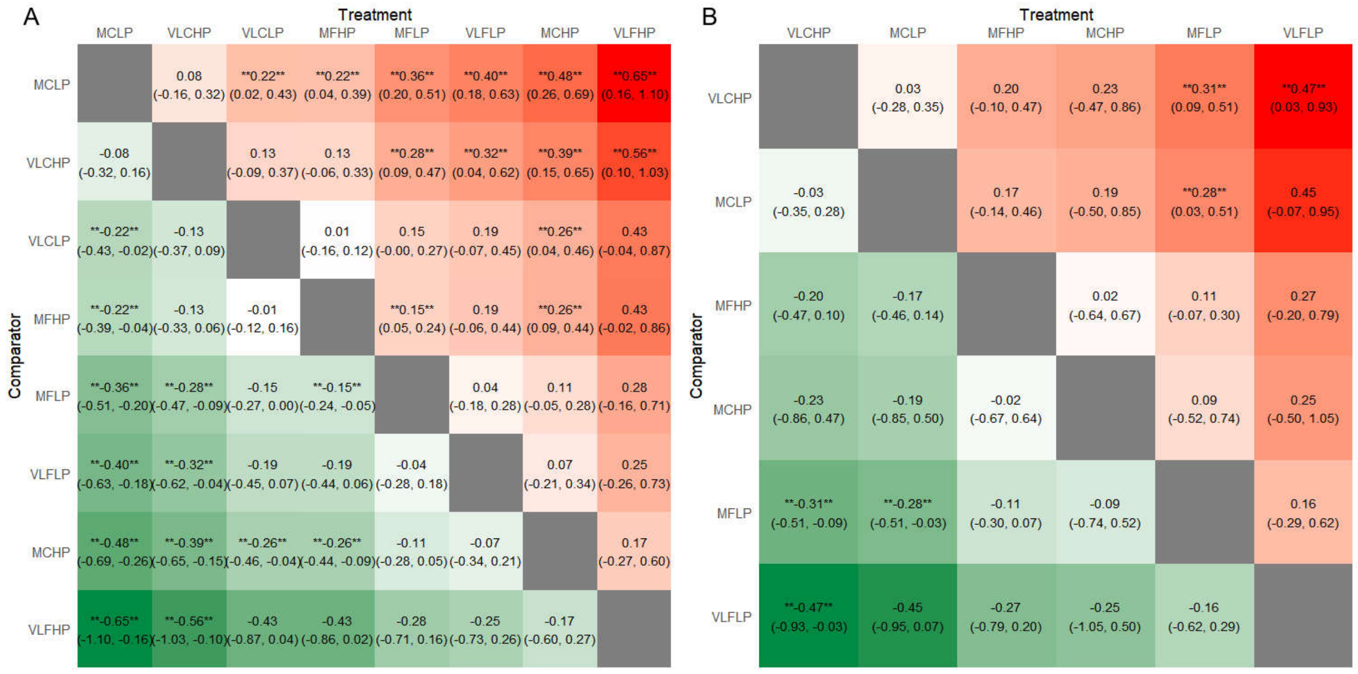

Figure S73 Ranking list heat map of total cholesterol in subgroups (mean difference and 95% credible interval). A, intervention period≤6 months. B, intervention period > 6 months.

Figure S74-81: Sensitivity analyses limiting the study to low risk of bias.

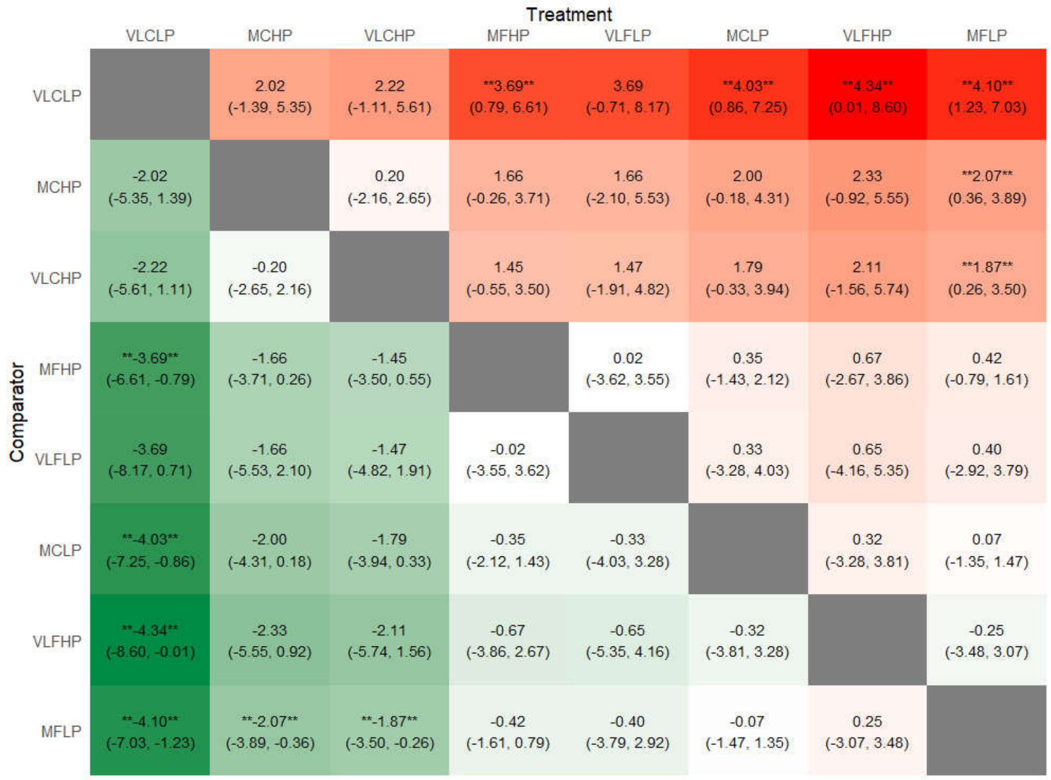

Figure S74 Comparative effects of different dietary groups on body weight in studies with low risk of bias (mean difference and 95% credible interval).

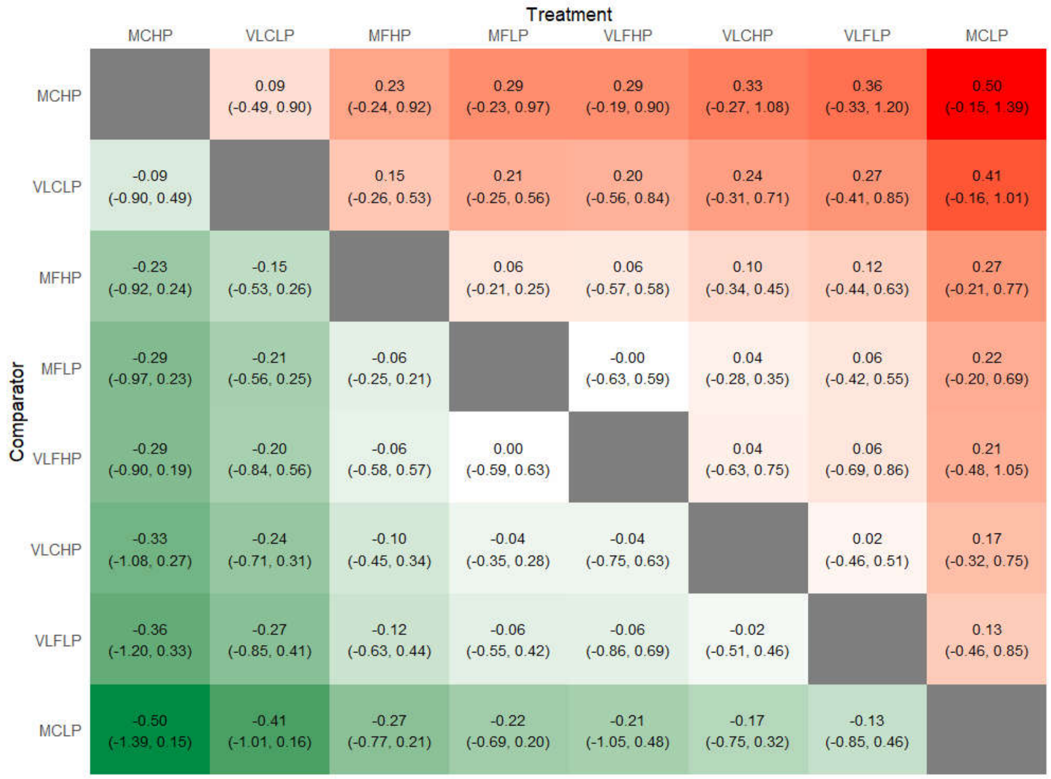

Figure S75 Comparative effects of different dietary groups on blood glucose in studies with low risk of bias (mean difference and 95% credible interval).

Comparator \ Treatment	MCHP	VLFHP	VLCHP	MFHP	MCLP	MFLP
MCHP		0.64 (-4.72, 4.84)	0.56 (-6.91, 6.70)	1.59 (-3.79, 5.03)	2.50 (-3.99, 7.54)	3.32 (-2.09, 7.23)
VLFHP	-0.64 (-4.84, 4.72)		0.08 (-7.51, 6.51)	1.01 (-4.32, 5.25)	1.98 (-4.60, 7.61)	2.80 (-2.93, 7.51)
VLCHP	-0.56 (-6.70, 6.91)	-0.08 (-6.51, 7.51)		0.83 (-4.42, 6.29)	1.86 (-3.99, 7.71)	2.64 (-1.97, 7.49)
MFHP	-1.59 (-5.03, 3.79)	-1.01 (-5.25, 4.32)	-0.83 (-6.29, 4.42)		1.02 (-3.09, 5.19)	1.82 (-0.68, 4.31)
MCLP	-2.50 (-7.54, 3.99)	-1.98 (-7.61, 4.60)	-1.86 (-7.71, 3.99)	-1.02 (-5.19, 3.09)		0.79 (-2.54, 4.10)
MFLP	-3.32 (-7.23, 2.09)	-2.80 (-7.51, 2.93)	-2.64 (-7.49, 1.97)	-1.82 (-4.31, 0.68)	-0.79 (-4.10, 2.54)	

Figure S76 Comparative effects of different dietary groups on systolic blood pressure in studies with low risk of bias (mean difference and 95% credible interval).

Comparator \ Treatment	MFHP	MCHP	VLFHP	VLCHP	MCLP	MFLP
MFHP		0.22 (-2.62, 3.56)	0.31 (-3.01, 3.91)	1.68 (-1.88, 4.97)	1.71 (-0.79, 4.28)	**1.93** (0.42, 3.49)
MCHP	-0.22 (-3.56, 2.62)		0.09 (-3.31, 3.32)	1.44 (-3.44, 5.64)	1.45 (-2.57, 5.12)	1.71 (-1.76, 4.83)
VLFHP	-0.31 (-3.91, 3.01)	-0.09 (-3.32, 3.31)		1.36 (-3.72, 5.86)	1.34 (-2.86, 5.50)	1.60 (-2.18, 5.28)
VLCHP	-1.68 (-4.97, 1.88)	-1.44 (-5.64, 3.44)	-1.36 (-5.86, 3.72)		0.01 (-3.51, 3.85)	0.29 (-2.72, 3.44)
MCLP	-1.71 (-4.28, 0.79)	-1.45 (-5.12, 2.57)	-1.34 (-5.50, 2.86)	-0.01 (-3.85, 3.51)		0.27 (-1.81, 2.22)
MFLP	**-1.93** (-3.49, -0.42)	-1.71 (-4.83, 1.76)	-1.60 (-5.28, 2.18)	-0.29 (-3.44, 2.72)	-0.27 (-2.22, 1.81)	

Figure S77 Comparative effects of different dietary groups on diastolic blood pressure in studies with low risk of bias (mean difference and 95% credible interval).

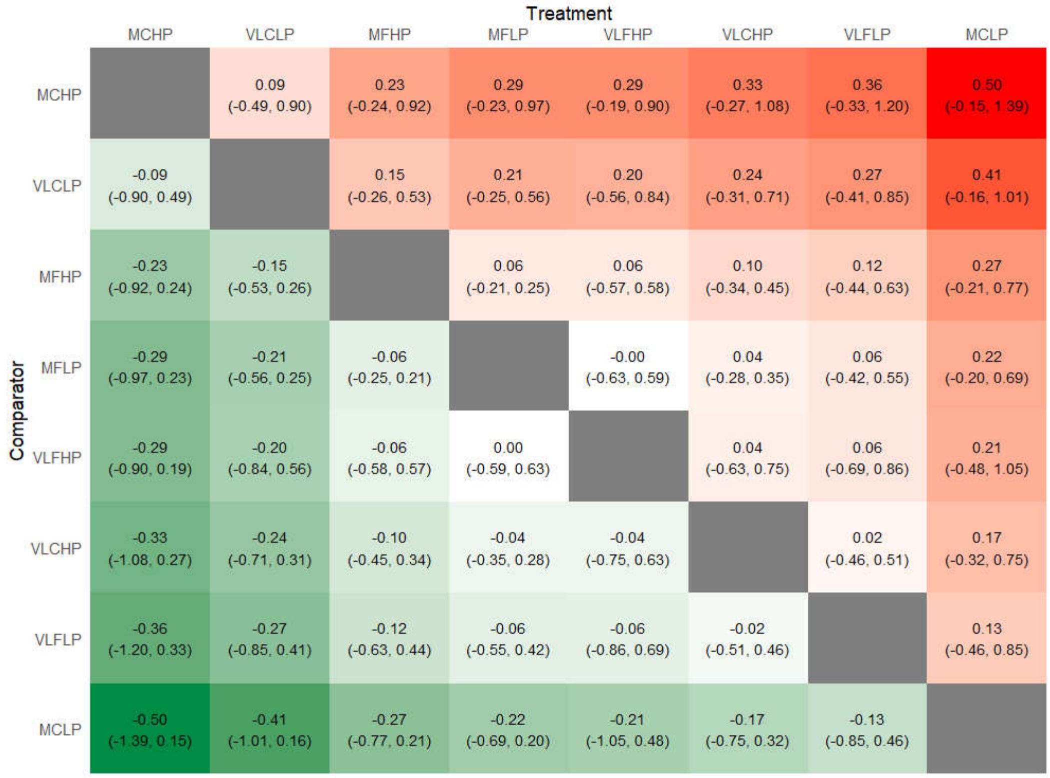

Figure S78 Comparative effects of different dietary groups on HDL cholesterol in studies with low risk of bias (mean difference and 95% credible interval).

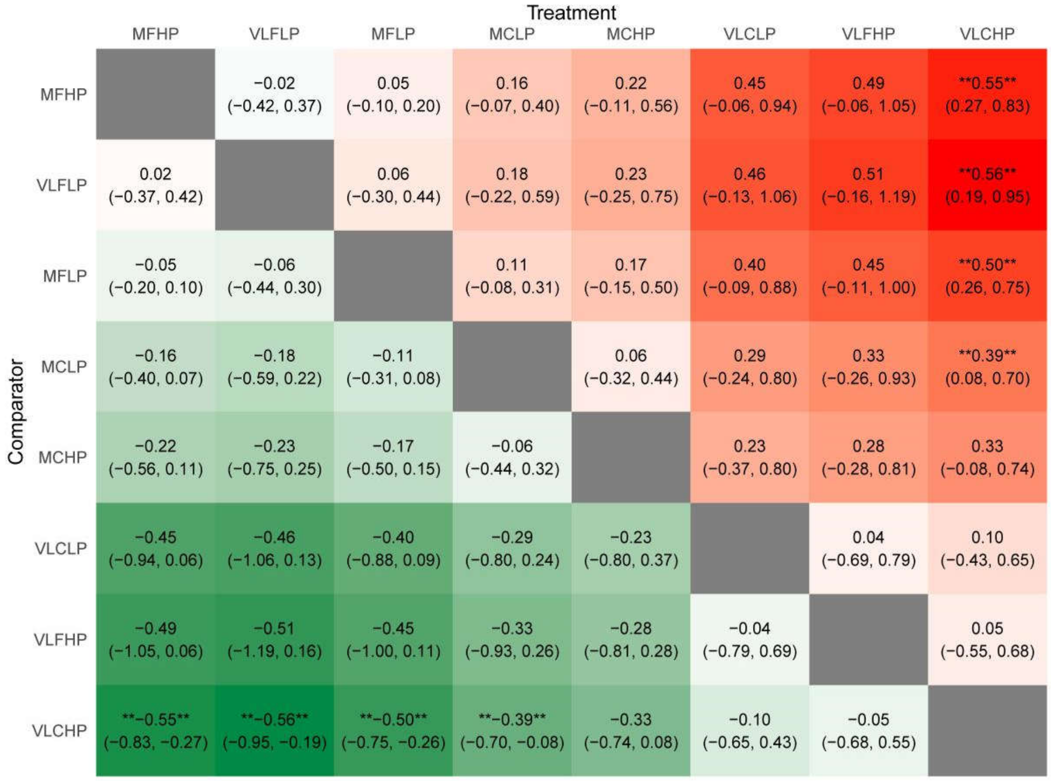

Figure S79 Comparative effects of different dietary groups on LDL cholesterol in studies with low risk of bias (mean difference and 95% credible interval).

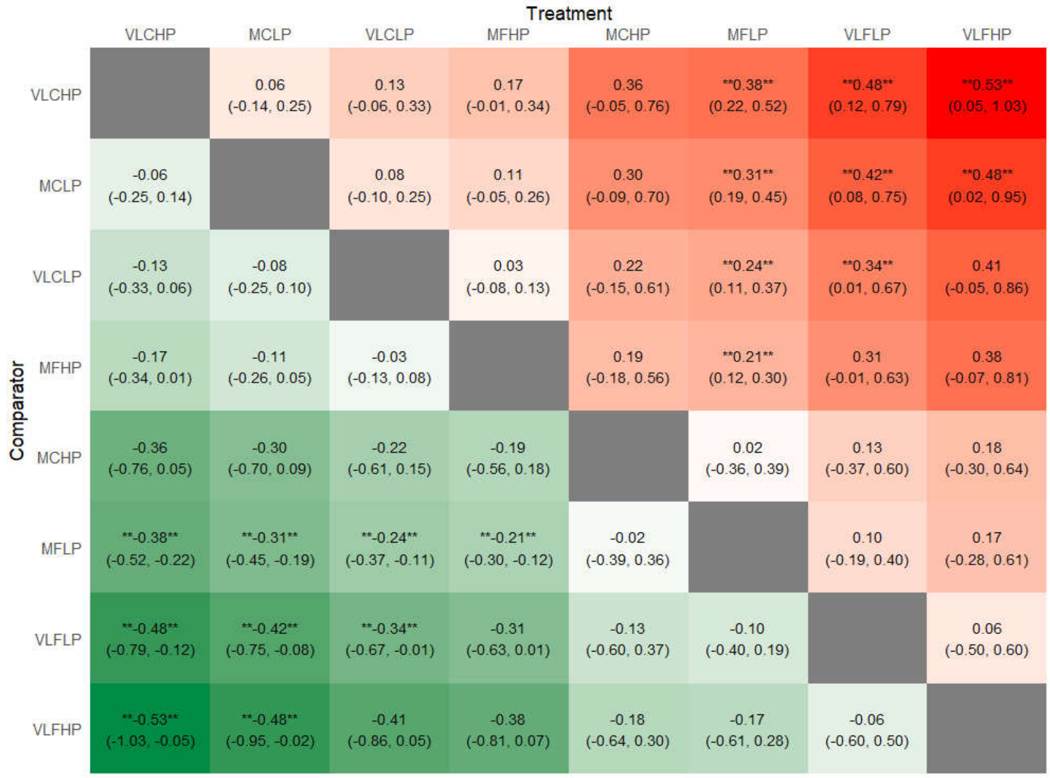

Figure S80 Comparative effects of different dietary groups on triglyceride in studies with low risk of bias (mean difference and 95% credible interval).

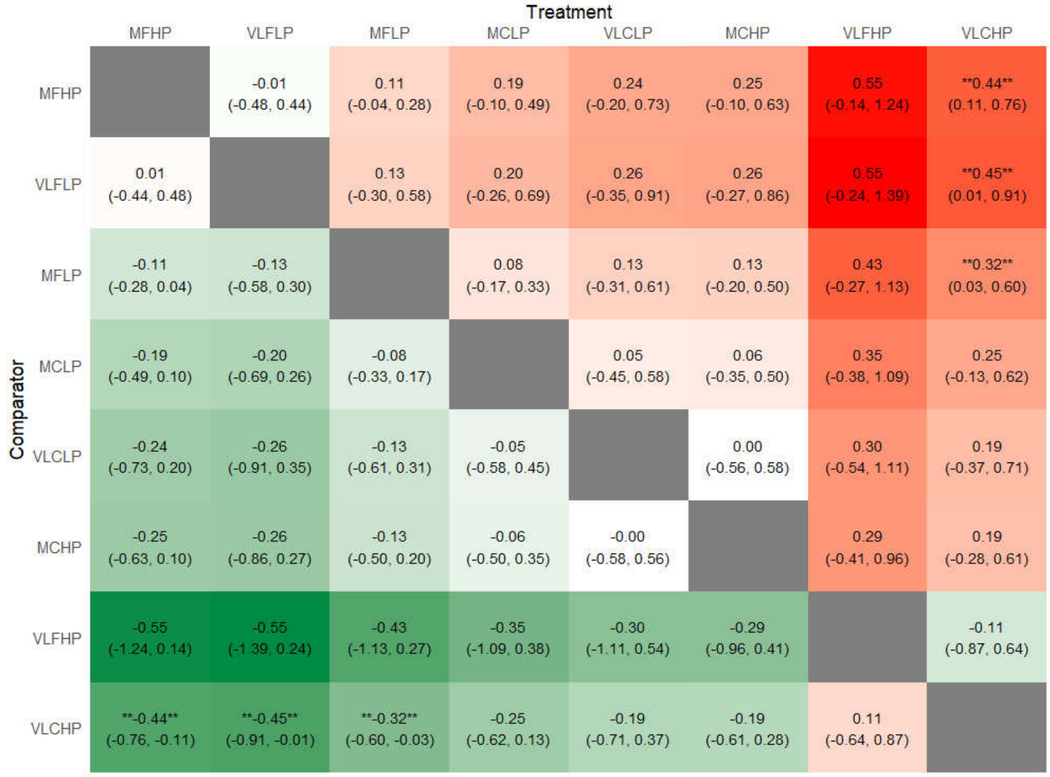

Figure S81 Comparative effects of different dietary groups on total cholesterol in studies with low risk of bias (mean difference and 95% credible interval).

Figure S82-85: Sensitivity analyses restricting the study to overweight and/or obese participants.

Treatment (columns) / Comparator (rows)

Comparator \ Treatment	VLCLP	MCHP	VLCHP	MFHP	MCLP	MFLP	VLFHP	VLFLP
VLCLP		2.58 (-0.39, 5.46)	2.78 (-0.10, 5.53)	**3.65** (1.02, 6.24)	**3.98** (1.14, 6.82)	**4.11** (1.50, 6.70)	**4.62** (0.78, 8.41)	**4.47** (1.18, 7.65)
MCHP	-2.58 (-5.46, 0.39)		0.19 (-1.61, 1.99)	1.07 (-0.44, 2.63)	1.40 (-0.38, 3.25)	**1.53** (0.17, 2.95)	2.04 (-0.74, 4.84)	1.88 (-0.52, 4.24)
VLCHP	-2.78 (-5.53, 0.10)	-0.19 (-1.99, 1.61)		0.88 (-0.46, 2.29)	1.22 (-0.40, 2.88)	**1.34** (0.21, 2.54)	1.85 (-1.15, 4.88)	1.70 (-0.24, 3.63)
MFHP	**-3.65** (-6.24, -1.02)	-1.07 (-2.63, 0.44)	-0.88 (-2.29, 0.46)		0.33 (-1.09, 1.75)	0.46 (-0.38, 1.33)	0.98 (-1.84, 3.72)	0.81 (-1.35, 2.87)
MCLP	**-3.98** (-6.82, -1.14)	-1.40 (-3.25, 0.38)	-1.22 (-2.88, 0.40)	-0.33 (-1.75, 1.09)		0.12 (-1.04, 1.31)	0.64 (-2.43, 3.65)	0.48 (-1.83, 2.69)
MFLP	**-4.11** (-6.70, -1.50)	**-1.53** (-2.95, -0.17)	**-1.34** (-2.54, -0.21)	-0.46 (-1.33, 0.38)	-0.12 (-1.31, 1.04)		0.52 (-2.35, 3.28)	0.36 (-1.66, 2.26)
VLFHP	**-4.62** (-8.41, -0.78)	-2.04 (-4.84, 0.74)	-1.85 (-4.88, 1.15)	-0.98 (-3.72, 1.84)	-0.64 (-3.65, 2.43)	-0.52 (-3.28, 2.35)		-0.17 (-3.61, 3.25)
VLFLP	**-4.47** (-7.65, -1.18)	-1.88 (-4.24, 0.52)	-1.70 (-3.63, 0.24)	-0.81 (-2.87, 1.35)	-0.48 (-2.69, 1.83)	-0.36 (-2.26, 1.66)	0.17 (-3.25, 3.61)	

Figure S82 Comparative effects of different dietary groups on body weight in participants with overweight and/or obesity (mean difference and 95% credible interval).

Treatment (columns) / Comparator (rows)

Comparator \ Treatment	MCLP	VLCLP	MCHP	MFLP	VLCHP	MFHP	VLFHP	VLFLP
MCLP		0.01 (-0.39, 0.39)	0.05 (-0.38, 0.46)	0.09 (-0.18, 0.34)	0.12 (-0.22, 0.42)	0.13 (-0.16, 0.40)	0.28 (-0.31, 0.84)	0.30 (-0.14, 0.71)
VLCLP	-0.01 (-0.39, 0.39)		0.04 (-0.41, 0.50)	0.08 (-0.23, 0.38)	0.11 (-0.25, 0.46)	0.12 (-0.19, 0.44)	0.27 (-0.32, 0.86)	0.29 (-0.16, 0.74)
MCHP	-0.05 (-0.46, 0.38)	-0.04 (-0.50, 0.41)		0.04 (-0.30, 0.38)	0.07 (-0.33, 0.45)	0.08 (-0.27, 0.43)	0.23 (-0.27, 0.72)	0.25 (-0.24, 0.73)
MFLP	-0.09 (-0.34, 0.18)	-0.08 (-0.38, 0.23)	-0.04 (-0.38, 0.30)		0.03 (-0.16, 0.22)	0.04 (-0.10, 0.19)	0.18 (-0.33, 0.70)	0.20 (-0.13, 0.56)
VLCHP	-0.12 (-0.42, 0.22)	-0.11 (-0.46, 0.25)	-0.07 (-0.45, 0.33)	-0.03 (-0.22, 0.16)		0.01 (-0.22, 0.25)	0.15 (-0.39, 0.71)	0.17 (-0.15, 0.52)
MFHP	-0.13 (-0.40, 0.16)	-0.12 (-0.44, 0.19)	-0.08 (-0.43, 0.27)	-0.04 (-0.19, 0.10)	-0.01 (-0.25, 0.22)		0.15 (-0.37, 0.65)	0.17 (-0.20, 0.53)
VLFHP	-0.28 (-0.84, 0.31)	-0.27 (-0.86, 0.32)	-0.23 (-0.72, 0.27)	-0.18 (-0.70, 0.33)	-0.15 (-0.71, 0.39)	-0.15 (-0.65, 0.37)		0.02 (-0.60, 0.64)
VLFLP	-0.30 (-0.71, 0.14)	-0.29 (-0.74, 0.16)	-0.25 (-0.73, 0.24)	-0.20 (-0.56, 0.13)	-0.17 (-0.52, 0.15)	-0.17 (-0.53, 0.20)	-0.02 (-0.64, 0.60)	

Figure S83 Comparative effects of different dietary groups on blood glucose in participants with overweight and/or obesity (mean difference and 95% credible interval).

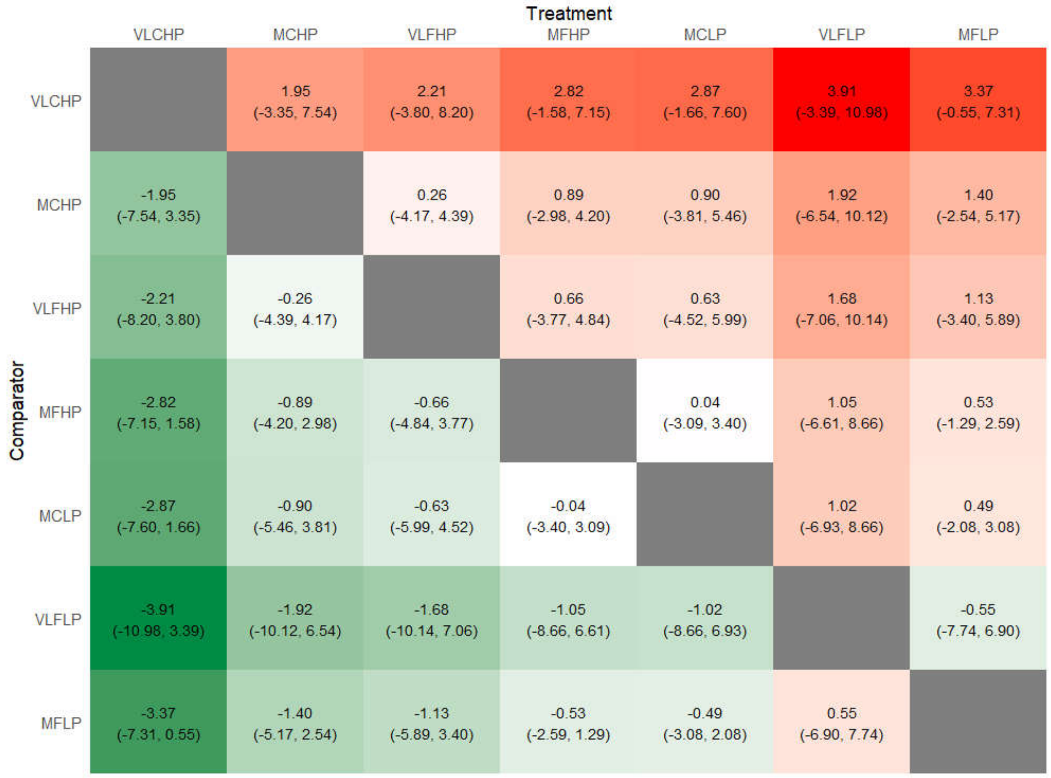

Fig.S84 Comparative effects of different dietary groups on systolic blood pressure in participants with overweight and/or obesity (mean difference and 95% credible interval).

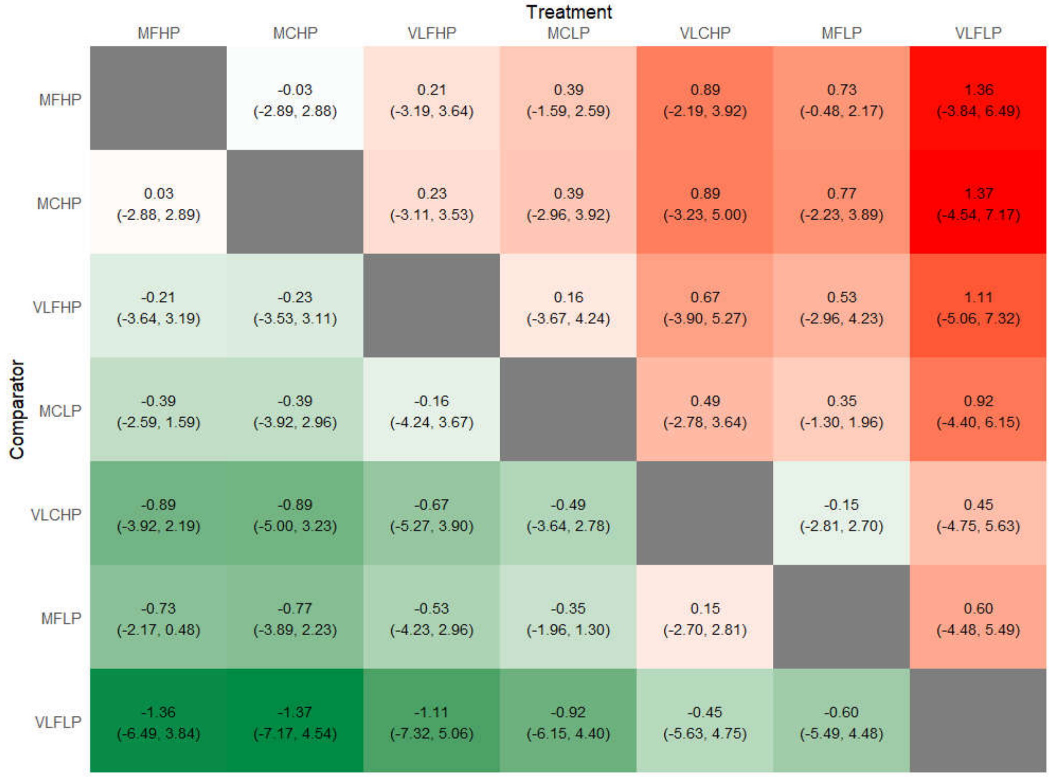

Figure S85 Comparative effects of different dietary groups on DBP in participants with overweight and/or obesity (mean difference and 95% credible interval).

References

1. Yancy WS Jr, Olsen MK, Guyton JR, Bakst RP, Westman EC. A low-carbohydrate, ketogenic diet versus a low-fat diet to treat obesity and hyperlipidemia: a randomized, controlled trial. *Ann Intern Med* 2004;140(10):769-77.

2. Hansen CD, Gram-Kampmann EM, Hansen JK, Hugger MB, Madsen BS, Jensen JM, Olesen S, Torp N, Rasmussen DN, Kjærgaard M, Johansen S, et al. Effect of Calorie-Unrestricted Low-Carbohydrate, High-Fat Diet Versus High-Carbohydrate, Low-Fat Diet on Type 2 Diabetes and Nonalcoholic Fatty Liver Disease: A Randomized Controlled Trial. *Ann Intern Med* 2023;176(1):10-21.

3. Tay J, Thompson CH, Luscombe-Marsh ND, Wycherley TP, Noakes M, Buckley JD, Wittert GA, Yancy WS Jr, Brinkworth GD. Effects of an energy-restricted low-carbohydrate, high unsaturated fat/low saturated fat diet versus a high-carbohydrate, low-fat diet in type 2 diabetes: A 2-year randomized clinical trial. *Diabetes Obes Metab* 2018;20(4):858-871.

4. Guo H, Wang L, Huang X, Shen F, Lu Y, Zhang P. Effects of low-carbohydrate vs low-fat diets on weight loss and metabolic risk factors in obese/overweight individuals with impaired glucose regulation: A randomized controlled trial. *Asia Pac J Clin Nutr* 2022;31(3):512-519.

5. Guldbrand H, Dizdar B, Bunjaku B, Lindström T, Bachrach-Lindström M, Fredrikson M, Ostgren CJ, Nystrom FH. In type 2 diabetes, randomisation to advice to follow a low-carbohydra te diet transiently improves glycaemic control compared with advice to follow a low-fat diet producing a similar weight loss. *Diabetologia* 2012;55(8):2118-27.

6. Lim SS, Noakes M, Keogh JB, Clifton PM. Long-term effects of a low carbohydrate, low fat or high unsaturated f at diet compared to a no-intervention control. *Nutr Metab Cardiovasc Dis*. 2010/10// 20(8):599-607.

7. Tay J, Thompson CH, Luscombe-Marsh ND, Noakes M, Buckley JD, Wittert GA, Brinkworth GD. Long-Term Effects of a Very Low Carbohydrate Compared With a High Carbohydrate Diet on Renal Function in Individuals With Type 2 Diabetes: A Randomized Trial. *Medicine*. Nov 2015;94(47):e2181.

8. Wycherley TP, Buckley JD, Noakes M, Clifton PM, Brinkworth GD. Long-term effects of a very low-carbohydrate weight loss diet on exercise capacity and tolerance in overweight and obese adults. *J Am Coll Nutr* 2014;33(4):267-73.

9. Brinkworth GD, Noakes M, Buckley JD, Keogh JB, Clifton PM. Long-term effects of a very-low-carbohydrate weight loss diet compared with an isocaloric low-fat diet after 12 mo. *Am J Clin Nutr* 2009;90(1):23-32.

10. Brinkworth GD, Luscombe-Marsh ND, Thompson CH, Noakes M, Buckley JD, Wittert G, Wilson CJ. Long-term effects of very low-carbohydrate and high-carbohydrate weight-loss diets on psychological health in obese adults with type 2 diabetes: randomized controlled trial. *J Intern Med* 2016;280(4):388-97.

11. Tay J, Luscombe-Marsh ND, Thompson CH, Noakes M, Buckley JD, Wittert GA, Yancy WS Jr, Brinkworth GD. A very low-carbohydrate, low-saturated fat diet for type 2 diabetes management: a randomized trial. *Diabetes Care* 2014;37(11):2909-18.

12. Wycherley TP, Brinkworth GD, Keogh JB, Noakes M, Buckley JD, Clifton PM. Long-term effects of weight loss with a very low carbohydrate and low fat diet on vascular function in overweight and obese patients. *J Intern Med* 2010;267(5):452-61.

13. Bradley U, Spence M, Courtney CH, McKinley MC, Ennis CN, McCance DR, McEneny J, Bell PM, Young IS, Hunter SJ. Low-fat versus low-carbohydrate weight reduction diets: effects on weight loss, insulin resistance, and cardiovascular risk: a randomized control trial. *Diabetes* 2009;58(12):2741-8.

14. Tay J, Brinkworth GD, Noakes M, Keogh J, Clifton PM. Metabolic effects of weight loss on a very-low-carbohydrate diet compared with an isocaloric high-carbohydrate diet in abdominally obese subjects. *J Am Coll Cardiol* 2008;51(1):59-67.

15. Guldbrand H, Lindström T, Dizdar B, Bunjaku B, Östgren CJ, Nystrom FH, Bachrach-Lindström M. Randomization to a low-carbohydrate diet advice improves health related quality of life compared with a low-fat diet at similar weight-loss in Type 2 diabetes mellitus. *Diabetes Res Clin Pract* 2014;106(2):221-7.

16. Soenen S, Bonomi AG, Lemmens SG, Scholte J, Thijssen MA, van Berkum F, Westerterp-Plantenga MS. Relatively high-protein or 'low-carb' energy-restricted diets for body weight loss and body weight maintenance? *Physiology & behavior* 2012;107(3):374-80.

17. Brinkworth GD, Buckley JD, Noakes M, Clifton PM. Renal function following long-term weight loss in individuals with abdominal obesity on a very-low-carbohydrate diet vs high-carbohydrate diet. *J Am Diet Assoc* 2010;110(4):633-8.

18. Stoernell CK, Tangney CC, Rockway SW. Short-term changes in lipoprotein subclasses and C-reactive protein levels of hypertriglyceridemic adults on low-carbohydrate and low-fat diets. *Nutr Res* 2008;28(7):443-9.

19. Golay A, Allaz AF, Morel Y, de Tonnac N, Tankova S, Reaven G. Similar weight loss with low- or high-carbohydrate diets. *The American journal of clinical nutrition* 1996;63(2):174-8.

20. Volek JS, Phinney SD, Forsythe CE, Quann EE, Wood RJ, Puglisi MJ, Kraemer WJ, Bibus DM, Fernandez ML, Feinman RD. Carbohydrate restriction has a more favorable impact on the metabolic syndrome than a low fat diet. *Lipids* 2009;44(4):297-309.

21. Struik NA, Brinkworth GD, Thompson CH, Buckley JD, Wittert G, Luscombe-Marsh ND. Very Low and Higher Carbohydrate Diets Promote Differential Appetite Responses in Adults with Type 2 Diabetes: A Randomized Trial. *The Journal of nutrition* 2020;150(4):800-805.

22. Mueller C, Masri B, Hogg J, Mastrogiacomo M, Chiu YL. Carbohydrate- vs fat-controlled diet effect on weight loss and coronary artery disease risk: a pilot feeding study. *Nutr Clin Pract* 2010;25(5):542-7.

23. Noakes M, Foster PR, Keogh JB, James AP, Mamo JC, Clifton PM. Comparison of isocaloric very low carbohydrate/high saturated fat and high carbohydrate/low saturated fat diets on body composition and cardiovascular risk. *Nutrition & metabolism* 2006;3:7.

24. Tay J, Luscombe-Marsh ND, Thompson CH, Noakes M, Buckley JD, Wittert GA, Yancy WS Jr, Brinkworth GD. Comparison of low- and high-carbohydrate diets for type 2 diabetes management: a randomized trial. *Am J Clin Nutr* 2015;102(4):780-90.

25. Ruth MR, Port AM, Shah M, Bourland AC, Istfan NW, Nelson KP, Gokce N, Apovian CM. Consuming a hypocaloric high fat low carbohydrate diet for 12 weeks lowers C-reactive protein, and raises serum adiponectin and high density lipoprotein-cholesterol in obese subjects. *Metabolism* 2013;62(12):1779-87.

26. Lim JJ, Liu Y, Lu LW, Barnett D, Sequeira IR, Poppitt SD. Does a Higher Protein Diet Promote Satiety and Weight Loss Independent of Carbohydrate Content? An 8-Week Low-Energy Diet (LED) Intervention. *Nutrients* 2022;14(3).

27. McAuley KA, Hopkins CM, Smith KJ, McLay RT, Williams SM, Taylor RW, Mann JI. Comparison of high-fat and high-protein diets with a high-carbohydrate diet in insulin-resistant obese women. *Diabetologia* 2005;48(1):8-16.

28. Jenkins DJ, Wong JM, Kendall CW, Esfahani A, Ng VW, Leong TC, Faulkner DA, Vidgen E, Paul G, Mukherjea R, et al. Effect of a 6-month vegan low-carbohydrate ('Eco-Atkins') diet on card iovascular risk factors and body weight in hyperlipidaemic adults: a r andomised controlled trial. *BMJ Open* 2014;4(2):e003505.

29. Morenga LT, Williams S, Brown R, Mann J. Effect of a relatively high-protein, high-fiber diet on body compositi on and metabolic risk factors in overweight women. *Eur J Clin Nutr* 2010;64(11):1323-31.

30. Brinkworth GD, Noakes M, Clifton PM, Buckley JD. Effects of a low carbohydrate weight loss diet on exercise capacity and tolerance in obese subjects. *Obesity* 2009;17(10):1916-23.

31. Brinkworth GD, Buckley JD, Noakes M, Clifton PM, Wilson CJ. Long-term effects of a very low-carbohydrate diet and a low-fat diet on mood and cognitive function. *Arch Intern Med* 2009;169(20):1873-80.

32. Halyburton AK, Brinkworth GD, Wilson CJ, Noakes M, Buckley JD, Keogh JB. Low- and high-carbohydrate weight-loss diets have similar effects on m ood but not cognitive performance. *Am J Clin Nutr* 2007;86(3):580-7.

33. Wycherley TP, Noakes M, Clifton PM, Cleanthous X, Keogh JB, Brinkworth GD. A high-protein diet with resistance exercise training improves weight loss and body composition in overweight and obese patients with type 2 diabetes. *Diabetes Care* 2010;33(5):969-76.

34. Luscombe-Marsh ND, Noakes M, Wittert GA, Keogh JB, Foster P, Clifton PM. Carbohydrate-restricted diets high in either monounsaturated fat or protein are equally effective at promoting fat loss and improving blood lipids. *Am J Clin Nutr* 2005;81(4):762-72.

35. Wycherley TP, Buckley JD, Noakes M, Clifton PM, Brinkworth GD. Correction to: Comparison of the effects of weight loss from a high-protein versus standard-protein energy-restricted diet on strength and aerobic capacity in overweight and obese men. *Eur J Nutr* 2013;59(8):3867.

36. Noakes M, Keogh JB, Foster PR, Clifton PM. Effect of an energy-restricted, high-protein, low-fat diet relative to a conventional high-carbohydrate, low-fat diet on weight loss, body composition, nutritional status, and markers of cardiovascular health in obese women. *Am J Clin Nutr* 2005;81(6):1298-306.

37. Marco-Benedí V, Pérez-Calahorra S, Bea AM, Lamiquiz-Moneo I, Baila-Rueda L, Cenarro A, Civeira F, Mateo-Gallego R. High-protein energy-restricted diets induce greater improvement in glucose homeostasis but not in adipokines comparing to standard-protein diets in early-onset diabetic adults with overweight or obesity. *Clin Nutr* 2020;39(5):1354-1363.

38. Johnston CS, Tjonn SL, Swan PD. High-protein, low-fat diets are effective for weight loss and favorably alter biomarkers in healthy adults. *J Nutr* 2004;134(3):586-91.

39. Krebs JD, Elley CR, Parry-Strong A, Lunt H, Drury PL, Bell DA, Robinson E, Moyes SA, Mann JI. The Diabetes Excess Weight Loss (DEWL) Trial: a randomised controlled trial of high-protein versus high-carbohydrate diets over 2 years in type 2 diabetes. *Diabetologia* 2012;55(4):905-14.

40. Sargrad KR, Homko C, Mozzoli M, Boden G. Effect of high protein vs high carbohydrate intake on insulin sensitivity, body weight, hemoglobin A1c, and blood pressure in patients with type 2 diabetes mellitus. *J Am Diet Assoc* 2005;105(4):573-80.

41. Kleiner RE, Hutchins AM, Johnston CS, Swan PD. Effects of an 8-week high-protein or high-carbohydrate diet in adults with hyperinsulinemia. *MedGenMed: Medscape general medicine* 2006;8(4):39.

42. Dalle Grave R, Calugi S, Gavasso I, El Ghoch M, Marchesini G. A randomized trial of energy-restricted high-protein versus high-carbohydrate, low-fat diet in morbid obesity. *Obesity* 2013;21(9):1774-81.

43. Griffin HJ, Cheng HL, O'Connor HT, Rooney KB, Petocz P, Steinbeck KS. Higher protein diet for weight management in young overweight women: a 12-month randomized controlled trial. *Diabetes Obes Metab* 2013;15(6):572-5.

44. Watson N, Dyer K, Buckley J, Brinkworth G, Coates A, Parfitt G, Howe P, Noakes M, Murphy K. Effects of Low-Fat Diets Differing in Protein and Carbohydrate Content on Cardiometabolic Risk Factors during Weight Loss and Weight Maintenance in Obese Adults with Type 2 Diabetes. *Nutrients* 2016;8(5).

45. Wycherley TP, Brinkworth GD, Clifton PM, Noakes M. Comparison of the effects of 52 weeks weight loss with either a high-protein or high-carbohydrate diet on body composition and cardiometabolic risk factors in overweight and obese males. *Nutr Diabetes* 2012;2(8):e40.

46. Gannon MC, Nuttall FQ. Effect of a high-protein, low-carbohydrate diet on blood glucose control in people with type 2 diabetes. *Diabetes* 2004;53(9):2375-82.

47. Elsahoryi NA, Alkurd RA, Subih H, Musharbash R. Effect of low-calorie ketogenic vs low-carbohydrate diets on body composition and other biomarkers of overweight/obese women: An 8 weeks randomised controlled trial. *Obesity Medicine* 2023;41:100496.

48. Brinkworth GD, Noakes M, Parker B, Foster P, Clifton PM. Long-term effects of advice to consume a high-protein, low-fat diet, rather than a conventional weight-loss diet, in obese adults with type 2 diabetes: one-year follow-up of a randomised trial. *Diabetologia* 2004;47(10):1677-86.

49. Calleja Fernández A, Vidal Casariego A, Cano Rodríguez I, Ballesteros Pomar MD. One-year effectiveness of two hypocaloric diets with different protein/carbohydrate ratios in weight loss and insulin resistance. *Nutr Hosp* 2012;27(6):2093-101.

50. Evangelista LS, Jose MM, Sallam H, Serag H, Golovko G, Khanipov K, Hamilton MA, Fonarow GC. High-protein vs. standard-protein diets in overweight and obese patients with heart failure and diabetes mellitus: findings of the Pro-HEART trial. *ESC Heart Fail* 2021;8(2):1342-1348.

51. Goss AM, Gower B, Soleymani T, Stewart M, Pendergrass M, Lockhart M, Krantz O, Dowla S, Bush N, Garr Barry V, et al. Effects of weight loss during a very low carbohydrate diet on specific adipose tissue depots and insulin sensitivity in older adults with obesity: a randomized clinical trial. *Nutr Metab* 2020;17:64.

52. Mateo-Gallego R, Marco-Benedí V, Perez-Calahorra S, Bea AM, Baila-Rueda L, Lamiquiz-Moneo I, de Castro-Orós I, Cenarro A, Civeira F. Energy-restricted, high-protein diets more effectively impact cardiometabolic profile in overweight and obese women than lower-protein diets. *Clin Nutr* 2017;36(2):371-379.

53. Nielsen JV, Jönsson E, Nilsson AK. Lasting improvement of hyperglycaemia and bodyweight: low-carbohydrate diet in type 2 diabetes--a brief report. *Ups J Med Sci* 2005;110(1):69-73.

54. Pedersen E, Jesudason DR, Clifton PM. High protein weight loss diets in obese subjects with type 2 diabetes mellitus. *Nutr Metab Cardiovasc Dis* 2014;24(5):554-62.

55. Segal-Isaacson CJ, Johnson S, Tomuta V, Cowell B, Stein DT. A randomized trial comparing low-fat and low-carbohydrate diets matched for energy and protein. *Obes Res* 2004;12 Suppl 2:130s-40s.

56. Brinkworth GD, Noakes M, Keogh JB, Luscombe ND, Wittert GA, Clifton PM. Long-term effects of a high-protein, low-carbohydrate diet on weight control and cardiovascular risk markers in obese hyperinsulinemic subjects. *Int J Obes Relat Metab Disord* 2004;28(5):661-70.

57. Wachsmuth NB, Aberer F, Haupt S, Schierbauer JR, Zimmer RT, Eckstein ML, Zunner B, Schmidt W, Niedrist T, Sourij H, et al. The Impact of a High-Carbohydrate/Low Fat vs. Low-

Carbohydrate Diet on Performance and Body Composition in Physically Active Adults: A Cross-Over Controlled Trial. *Nutrients* 2022;14(3).

58. Meckling KA, O'Sullivan C, Saari D. Comparison of a low-fat diet to a low-carbohydrate diet on weight loss, body composition, and risk factors for diabetes and cardiovascular disease in free-living, overweight men and women. *J Clin Endocrinol Metab* 2004;89(6):2717-23.

59. Volek JS, Sharman MJ, Gómez AL, DiPasquale C, Roti M, Pumerantz A, Kraemer WJ. Comparison of a very low-carbohydrate and low-fat diet on fasting lipids, LDL subclasses, insulin resistance, and postprandial lipemic responses in overweight women. *J Am Coll Nutr* 2004;23(2):177-84.

60. Te Morenga LA, Levers MT, Williams SM, Brown RC, Mann J. Comparison of high protein and high fiber weight-loss diets in women with risk factors for the metabolic syndrome: a randomized trial. *Nutr J* 2011;10:40.

61. Lewis SB, Wallin JD, Kane JP, Gerich JE. Effect of diet composition on metabolic adaptations to hypocaloric nutrition: comparison of high carbohydrate and high fat isocaloric diets. *Am J Clin Nutr* 1977;30(2):160-70.

62. Buga A, Welton GL, Scott KE, Atwell AD, Haley SJ, Esbenshade NJ, Abraham J, Buxton JD, Ault DL, Raabe AS, et al. The Effects of Carbohydrate versus Fat Restriction on Lipid Profiles in Highly Trained, Recreational Distance Runners: A Randomized, Cross-Over Trial. *Nutrients* 2022;14(6).

63. Abete I, Parra D, De Morentin BM, Alfredo Martinez J. Effects of two energy-restricted diets differing in the carbohydrate/protein ratio on weight loss and oxidative changes of obese men. *Int J Food Sci Nutr* 2009;60 Suppl 3:1-13.

64. Jansen LT, Yang N, Wong JMW, Mehta T, Allison DB, Ludwig DS, Ebbeling CB. Prolonged Glycemic Adaptation Following Transition From a Low- to High-Carbohydrate Diet: A Randomized Controlled Feeding Trial. *Diabetes Care* 2022;45(3):576-584.

65. Piatti PM, Monti F, Fermo I, Baruffaldi L, Nasser R, Santambrogio G, Librenti MC, Galli-Kienle M, Pontiroli AE, Pozza G. Hypocaloric high-protein diet improves glucose oxidation and spares lean body mass: comparison to hypocaloric high-carbohydrate diet. *Metabolism* 1994;43(12):1481-7.

66. Ebbeling CB, Knapp A, Johnson A, Wong JMW, Greco KF, Ma C, Mora S, Ludwig DS. Effects of a low-carbohydrate diet on insulin-resistant dyslipoproteinemia-a randomized controlled feeding trial. *Am J Clin Nutr* 2022;115(1):154-162.
